# Effect of cyclic substituents on the anti-cancer activity and DNA interaction of ruthenium(II) bis-phenanthroline dipyridoquinoline

**DOI:** 10.3389/fmolb.2023.1252285

**Published:** 2023-10-18

**Authors:** Etubonesi E. Nyong-Bassey, Andrew L. Hicks, Poppy Bergin, Eimer M. Tuite, Valery Kozhevnikov, Stephany Veuger

**Affiliations:** ^1^ Department of Applied Sciences, Faculty of Health and Life Sciences, Northumbria University, Newcastle upon Tyne, United Kingdom; ^2^ School of Natural and Environmental Science–Chemistry, Newcastle University, Newcastle upon Tyne, United Kingdom

**Keywords:** AFM, viscometry, intercalation, cytotoxicity, lipophilicity

## Abstract

**Introduction:** Ruthenium(II) complexes have emerged recently as candidates for anti-cancer therapy, where activity is related to lipohilicity, cellular localization, and specific interactions with biomolecules.

**Methods:** In this work, two novel complexes were synthesized and are reported based on the [Ru(phen)_2_(dipyrido[3,2-*f*:2′,3′-*h*]quinoxaline]^2+^ framework.

**Results:** Compared to the parent complex, annealing of cyclopenteno and cyclohexeno rings to the extended ligand substantially increased cytotoxicity towards a number of cancer cell lines, and induced apoptosis. The complexes localize in the nuclei of cancer cells and co-locate with DAPI on DNA. DNA binding studies show that both complexes bind strongly to DNA and one complex intercalates DNA like the parent, whilst the other appears to have multiple modes of interaction.

**Discussion:** It is likely that the increased lipophilicity of the novel complexes is a key factor for increasing their cytotoxicity, rather than their DNA binding mode.

## 1 Introduction

Since the discovery of cisplatin, the use of metallic complexes as anti-cancer agents has become a popular area of research. Metal chelation has made it possible to study a structure–activity relationship by not only modifying ligands but also the chelating metals. This has given rise to an array of interesting possibilities in the fight against cancer. Cisplatin-resistant cancer has posed a clinical challenge and has given rise to the development of other platinum-based complexes such as carboplatin and oxaliplatin (Dasari and Tchounwou, 2014). Although these complexes have proven effective, their use has been limited due to severe dose-limiting side effects and poor selectivity against normal cell lines. Studies on the cytotoxicity of ruthenium complexes as possible alternatives to platinum-based complexes have emerged recently. The development of ruthenium-based anti-cancer complexes has transcended from ruthenium (III) complexes such as NAMI and KP1019, which have undergone clinical trials, and ruthenium (II) arene complexes to polypyridyl ruthenium (II) complexes.

Furthermore, unlike cisplatin, which exerts its anti-cancer activity through ligand exchange, these complexes are coordinately saturated and substitutionally inert, thereby acting through a different mechanism. Although some researchers have reported that coordinately saturated polypyridyl complexes exert their cytotoxic activity through a non-covalent interaction with DNA (Hotze et al., 2005; Ma et al., 2007; Griffith et al., 2017), others have reported a role for other factors such as mitochondrial dysfunctional pathway activation (Pierroz et al., 2012), inhibition of topoisomerases I and II (Wang et al., 2014), and cell membrane structure and adhesion modification (Gill et al., 2013) in their anti-cancer properties. Many studies have also reported the link between the lipophilicity of polypyridyl ruthenium complexes and their cytotoxic activities in various cell lines. In most cases, the anti-cancer activities were enhanced by an increase in lipophilicity (Schafer et al., 2007; Schatzsneider et al., 2008; Notaro and Gasser, 2017). In this research, we used the structure–activity relationship (SAR) to study the cytotoxic activities of three novel polypyridyl ruthenium complexes. They comprise structurally related mono-metallic ruthenium complexes (VNK-754, VNK-572, and POW-12A). We sought to optimize the lead complex ruthenium (II) bis-phenanthroline dipyridoquinoline (VNK-754) by ligand modification through the annealing cyclopenteno (POW-12A) and cyclohexeno (VNK-572) fragments. We hypothesized that this would produce compounds with better cytotoxicity profiles, translating to selectively toxic anti-cancer agents.

**SCHEME 1:**
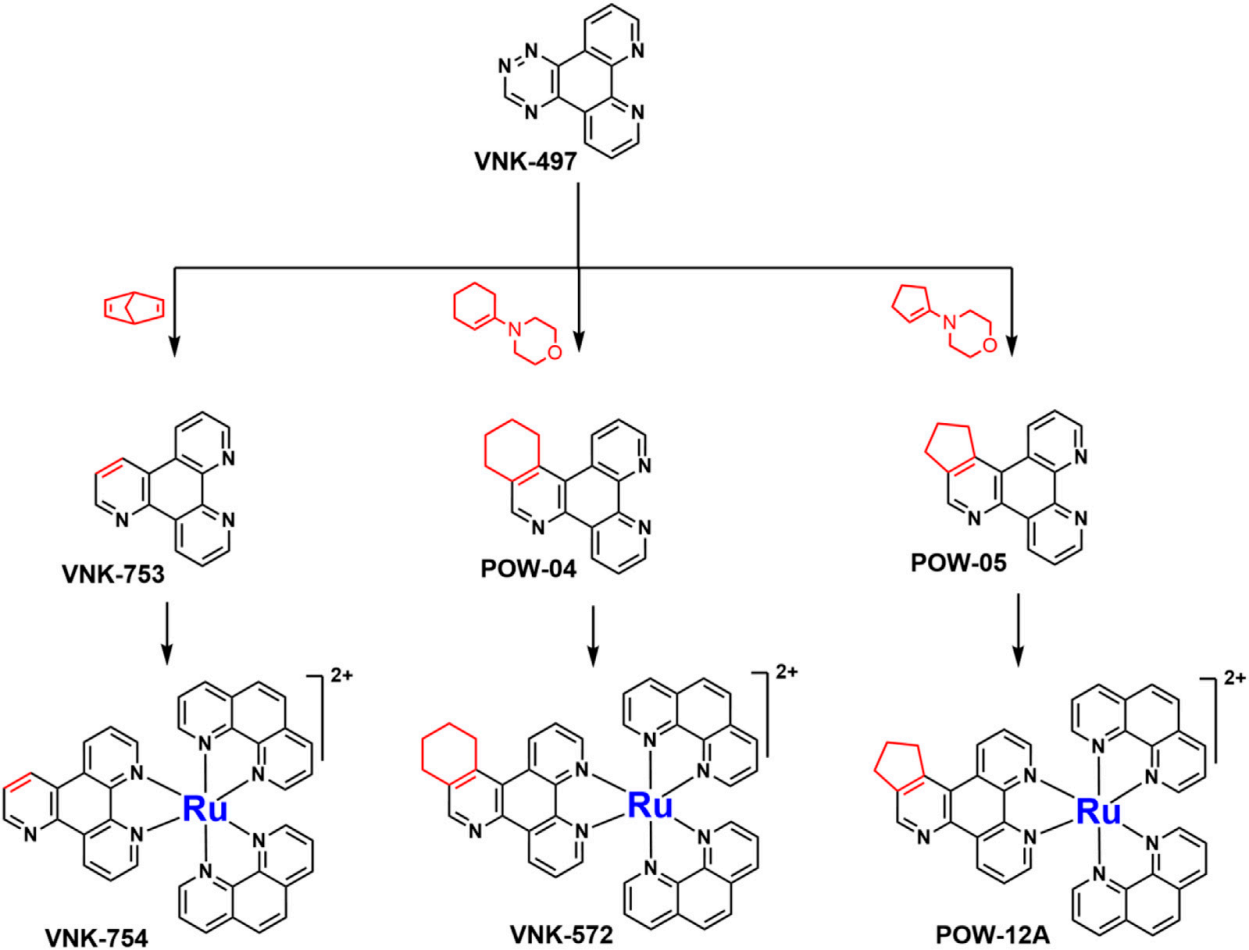
The use of 1,2,4-triazine methodology to prepare the ligands and the ruthenium (II) complexes described in this paper.

## 2 Results and discussion

### 2.1 Synthesis of ligands and complexes

Ligands determine lipophilicity, charge, and the size of the complex, all of which influence the cellular uptake, distribution, and localization of a complex within a cell. In this paper, we sought to prepare complexes with varying degrees of lipophilicity. The synthesis of polypyridine-type ligands via the intermediacy of 1,2,4-triazines is an attractive methodology for the functionalization of pyridine rings. The key step in this methodology is the inverse electron demand Diels–Alder reaction, where 1,2,4-triazine acts as an electron-poor diene. Dienophiles, such as 2,5-norbornadiene, and electron-rich enamines react with the 1,2,4-triazine ring to provide corresponding pyridines. For example, in their pioneering paper, Sauer and co-workers demonstrated that 1,2,4-triazine 4, which was prepared by the reaction of 1,10-phenanthroline-5,6-dione 3 with formamidrazone, can react with 2,5-norbornadiene to give a ligand VNK-753 (Scheme 1). To increase the lipophilicity of the ligands, we used 1-morpholinocyclohexene or 1-morpholinocyclopentene as dienophiles and accessed ligands POW-04 and POW-05 in good yields. The ligands were then used to synthesize the target complexes by reaction with Ru(phen)_2_Cl_2_, followed by treatment with ammonium hexafluorophosphate.


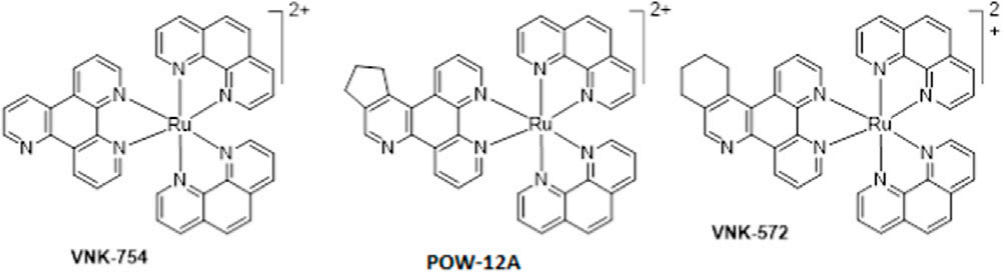
 
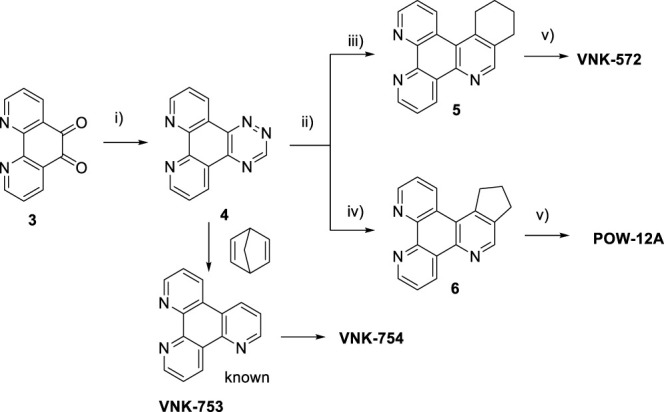


### 2.2 Cytotoxicity profiles

The cytotoxicity profile of the ruthenium complexes VNK-754, VNK-572, and POW-12A was assessed in various cancer cell lines, namely, human cervical cancer HeLa, prostate cancer cell line DU145, breast cancer cell line MDAMB231MB231, ovarian cancer cell line A2780, and cisplatin-resistant ovarian cancer cell line A2780-CP70, using the preferred screen of the National Cancer Institute, the sulphorhodamine (SRB) dye assay, and the trypan blue cell viability assay (Skehan et al., 1990; Houghton et al., 2007; Strober, 2001). To determine their selective toxicity profile, these complexes were also tested in a normal prostate cell line (PTN1A). From the similarity in chemical structures of the compounds, we deduced that a structure–activity relationship study will help elucidate key properties involved in the cytotoxic activity of this class of metal complexes.

Based on the results (Table 1), cancer cell lines showed various degrees of susceptibility to these complexes, with A2780 being the most susceptible and MDA-MB-231 (IC50 > 100 μM) being the least susceptible. VNK-572 and POW12A both showed significantly higher anti-cancer activity than VNK-754 in all the cancer cell lines tested. VNK-572 showed slightly higher but not significantly different anti-tumor activity than POW-12A in the same cell lines (*p* = 0.346 in HeLa; *p* = 0.183 in DU145). These results indicate that the addition of a six-membered or five-membered cyclic (cyclohexane or cyclopentane) substituent to ruthenium(II) bis-phenanthroline dipyridoquinoline (VNK-754), as seen in VNK-572 and POW-12A, respectively, enhances the cytotoxic activity of this complex to a comparable extent. For example, in the more susceptible cancer cell lines (A2780 and DU145), the structural modification produced a seven-fold difference between the anti-cancer activities of VNK-754 and VNK-572. Furthermore, although the IC_50_ values were typically an order of magnitude higher than those for cisplatin, crucially, they showed selective toxicity as, unlike cisplatin, they were less cytotoxic to normal prostate cells (PNT1A) than the prostate cancer cell line DU145; the cytotoxic activities of these complexes against the normal prostate cell line PTN1A were very low (all >100 μM) compared to cisplatin, which had an IC_50_ value of 3.8 μΜ. VNK-572 showed particular selectivity with a more than five-fold difference in sensitivity between PTN1A normal prostate cells (>100 μM) compared to the prostate cancer cell line DU145 (IC50 = 19 μM), thus displaying a favorable selective toxicity profile. This contrasted with cisplatin, which showed a very narrow window (fold difference = 0.6) between its cytotoxic activities against DU145 (IC50 = 2.4 μM) and the normal prostate cell line PNT1A (IC50 = 3.8 μM). Similar trends were seen for the trypan blue exclusion assays (Supplementary Material S2). Error bars showed some inter-assay variability that was more pronounced at lower doses for the trypan blue assays. This indicates a hormetic response, where low doses of a compound can elicit a stimulatory response. In addition, the compounds may vary in their cytotoxicity depending on the cell cycle stage. As the cells were not synchronized, this could also account for the inter-assay variability observed. The data were in line with other studies reporting Ru-complexes to have superior anti-cancer profiles, such as improved selectivity toward cancer cells. A2780-CP70 cells are cisplatin-resistant, showing a two-fold resistance factor (IC50 A2780-CP70/IC50 A2780). Similar differences in sensitivity were seen for all three ruthenium complexes. A2780-CP70 cells have been shown to be two-fold more efficient at effluxing drugs, leading to reduced total drug accumulation for equivalent micromolar drug exposure and being more efficient at repairing/tolerating lesions induced in the cellular DNA by cisplatin (Parker et al., 1991). This indicates that alterations in drug uptake/efflux or DNA repair may contribute to differences in the cellular cytotoxicity of these novel complexes.

**TABLE 1:** IC50 values of VNK-754, VNK-572, POW-12A, and cisplatin on various cell lines, as measured by the sulphorhodamine B assay.

Compound	HeLa cervical cancer	DU145 prostate cancer	PNT1A normal prostate	MDA-MB-231 (TNBC)	A2780 ovarian cancer	A280-CP70 cisplatin-resistant ovarian cancer
VNK-754	>100 μM	>100 μM	>100 μM	>100 μM	74 ± 14.43 μM	>100 μM
VNK-572	52 ± 6.35 μM	19 ± 2.31 μM	>100 μM	>100 μM	10 ± 4.04 μM	15 ± 2.89 μM
POW-12A	63 ± 6.35 μM	35 ± 6.93 μM	>100 μM	>100 μM	12 ± 1.73 μM	35 ± 3.47 μM
Cisplatin	6 ± 0.58 μM	2.4 ± 0.09 μM	3.8 ± 0.58 μM	37 ± 8.08 μM	2 ± 0.00 μM	4 ± 0.58 μM

Data are expressed as mean ± SEM.

Additionally, epifluorescence microscopy revealed that these complexes exhibited nuclear fragmentation, especially at higher doses (Supplementary Material S3, S4), while cisplatin displayed nuclear swelling at a concentration of 5 μM (Supplementary Material S3, S4). This may indicate a difference in the mode of cell death between these two classes of complexes, as nuclear swelling is one key characteristic of necrosis, while nuclear fragmentation characterizes apoptosis (Elmore, 2007).

### 2.3 Cellular localization

Ruthenium metal complexes have been reported to elicit their cytotoxicity through various sites. The major sites of activity include the nucleus, where they interact with DNA (Hotze et al., 2005; Ma et al., 2007; Griffith et al., 2017); the mitochondria, where they cause mitochondria dysfunctional pathway activation (Pierroz et al., 2012); and the cell membrane, where they cause changes in cell membrane structure and adhesion. We, therefore, decided to examine the cellular localization of our ruthenium complexes in order to assess their cellular distribution and possible site of action.

**FIGURE 1:**
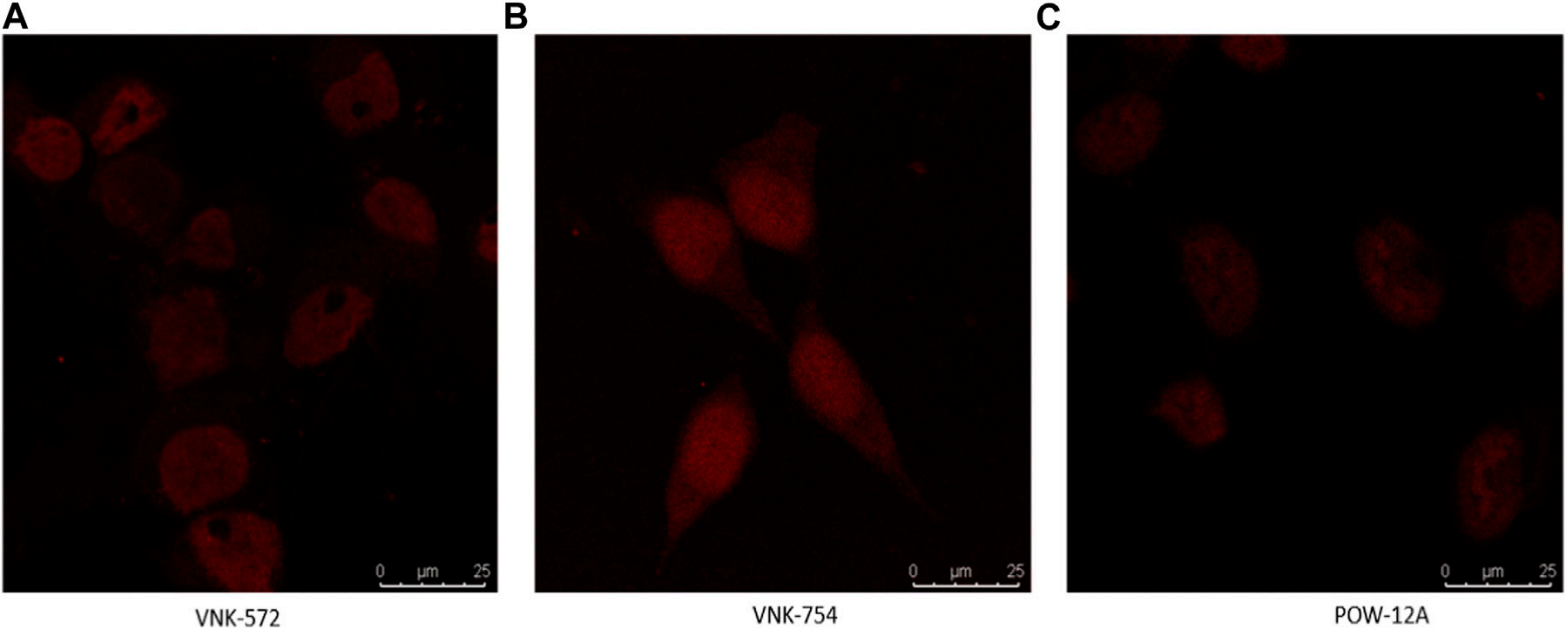
Confocal images of **(A)** VNK-572, **(B)** VNK-754, and **(C)** POW-12A nuclear localization in HeLa cells after respective treatments with the complexes alone. The complexes were viewed under a CLSM Leica SP microscope using the wavelength settings (excitation, 405 nm; emission, 570–630 nm).

Fortunately, like most Ru (II) polypyridyl complexes (Juris et al., 1988), these samples have fluorescence properties, making it possible to examine their location within the cell by fluorescence microscopy, as seen in Figure 1.

**FIGURE 2:**
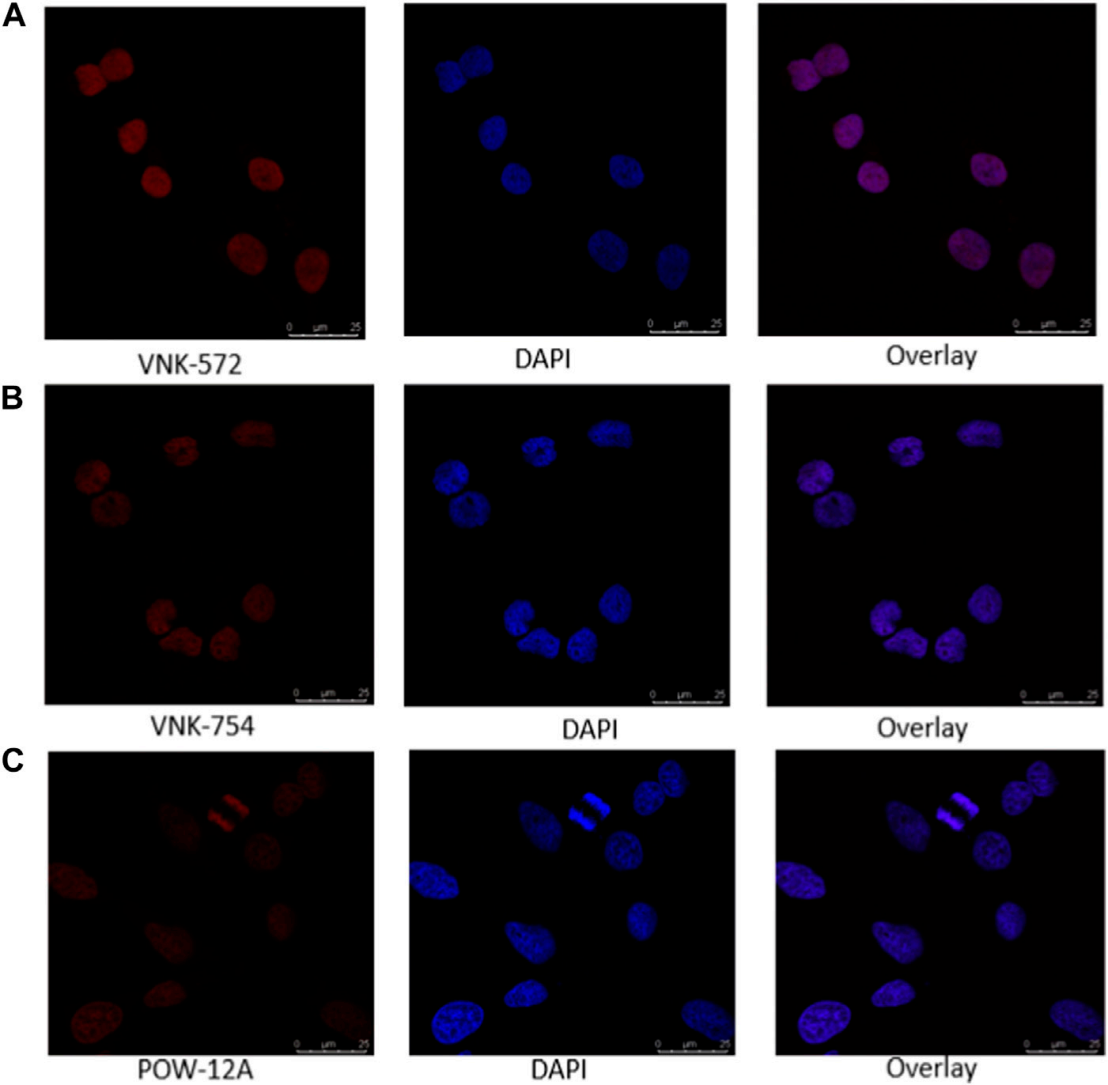
Confocal images of the nuclear localization of all three complexes in HeLa cells after incubating with **(A)** VNK-572 (50 μM), **(B)** VNK-754 (50 μM), and **(C)** POW-12A (50 μM). Images show the fluorescence of the compounds (red), DAPI (blue), and an overlay of each respective complex with DAPI after counter-staining. The complexes were viewed under the CLSM Leica SP microscope using the wavelength settings (excitation, 405 nm; emission, 570–630 nm), while DAPI was observed using the following settings (excitation, 405 nm; emission, 430–480 nm).

Confocal microscopy was used in this study, and the results are presented in Figures 1, 2. VNK-572, VNK-754, and POW-12A were shown to penetrate the nucleus of HeLa cells (Figure 1). This was further confirmed by the co-localization of these complexes with a nuclear counterstain (DAPI) (Figure 2). Additionally, images of all three complexes obtained in the prostate cancer cell line DU145 showed the same localization (Supplementary Material S7B).

This indicates that the cytotoxic activities of these complexes are likely linked to the nuclear penetration of these compounds and their interaction with nuclear DNA.

### 2.4 Partition coefficient (LogP)

The role of lipophilicity in the activity of ruthenium (II) polypyridyl complexes is one that has been extensively reported (Notaro and Gasser, 2017). Increasing the lipophilicity of these kinds of complexes in order to improve their cellular uptake across the semi-permeable membrane has been shown to enhance the cytotoxic effect of such compounds (Schafer et al., 2007; Schatzsneider et al., 2008). Based on this, we carried out a partition coefficient (log P) test using a shake-flask method to determine whether the marginal structural differences between VNK-754, VNK-572, and POW-12A affect their lipophilicity and to examine whether such a difference in lipophilicity confers any advantage to their anti-cancer activity. The log P results, as presented in Table 2, clearly show VNK-572 (0.014) and POW-12A (0.077) to be more lipophilic than VNK-754 (−0.874). The LogP values of POW-12A and VNK-572 were comparable with those of POW12A, which is slightly more lipophilic, although this is not statistically significant (*p* = 0.302). Changes to the structure of the parent complex have, therefore, resulted in a dramatic change in lipophilicity. For example, the fold difference between the logP value of VNK-572 and that of VNK-754 was 7.9. Thus, along with varied DNA intercalating properties, this explains, in part, why there are appreciable differences in the cytotoxic effects of VNK572 and POW12A compared to VNK-754, even though they are generally similar in structure. For example, VNK-572 with an additional benzene ring can penetrate the bi-lipid-layered cell membrane better and achieve higher cellular and nuclear concentrations than VNK-754, producing a greater anti-tumor effect.

**TABLE 2:** LogP values of VNK-754, VNK-572, and POW-12A, as determined using the shake-flask method using phosphate buffered saline (pH 7.4) as the aqueous phase and 1-octanol as the organic phase.

Compound	LogP
VNK-754	−0.874
VNK-572	0.014
POW-12A	0.077

### 2.5 Mechanism of action—cell cycle analysis

**FIGURE 3:**
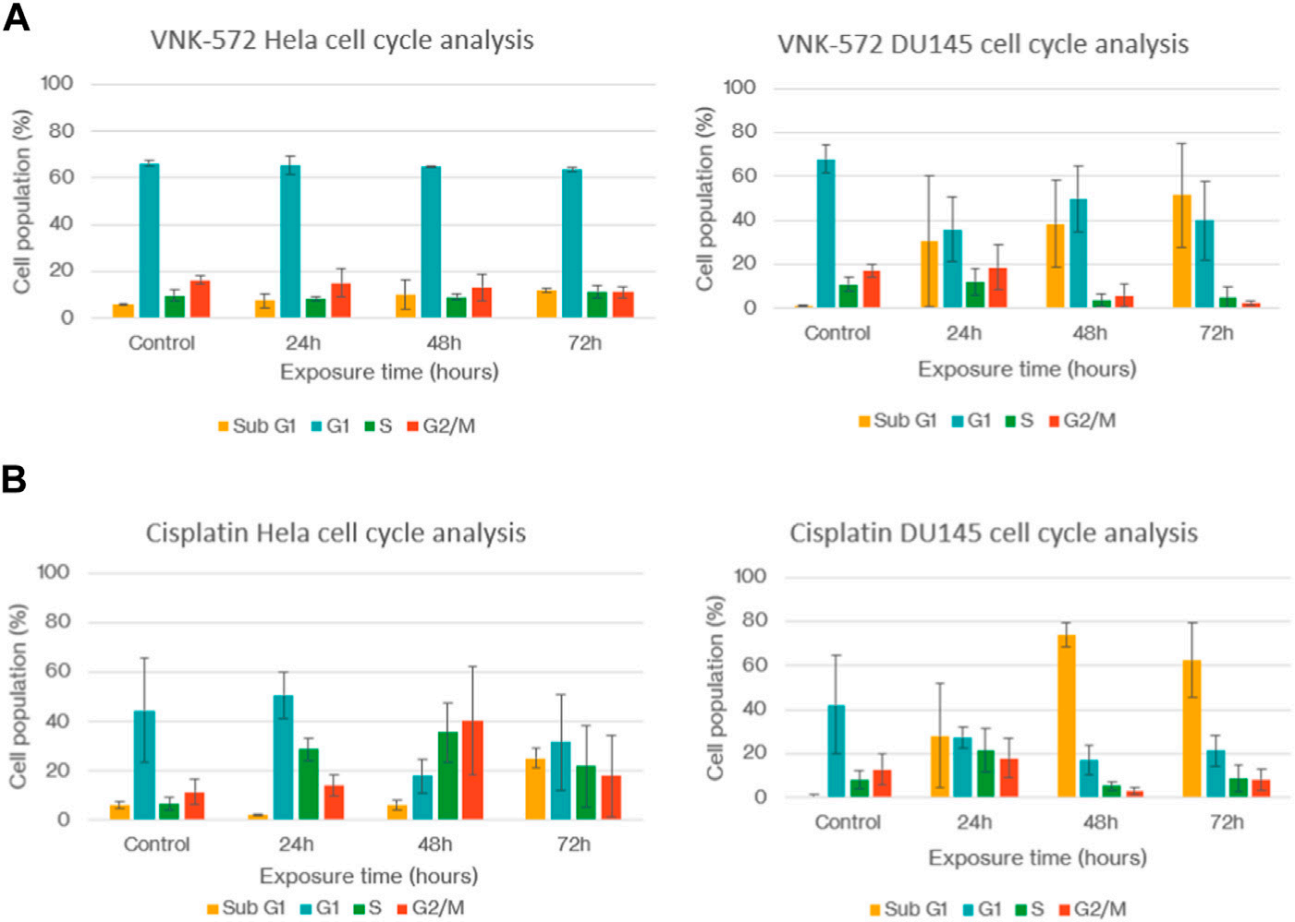
Graphs of **(A)** HeLa and DU145 cell cycles after treatment with VNK-572. **(B)** HeLa and DU145 cell cycles after treatment with cisplatin. Data are expressed as mean ± SEM.

The cell cycle of cervical HeLa and prostate DU145 cancer cells was studied after treatment with VNK-572 and cisplatin, respectively (Figure 3). VNK-572 and cisplatin both reduced the G2 population after 72 h compared to control (e.g., 16%–11% in Hela (*p* = 0.06) and from 17% to 2% in the DU145 cell line (*p* = 0.003) for VNK-572) (Figure 3A). VNK-572 saw only slight changes to the G1 or S phases in the HeLa cell line. The DU145 cells saw a decrease in both phases following 72 h of treatment—from 68% to 40% and from 11% to 5% for the G1 and S phases, respectively (Figure 3A). Cisplatin showed an increase in S-phase arrest, but only in the HeLa cells (Figure 3B). Importantly, the results showed that all cells were arrested in cell cycle progression, accumulating in the sub-G1 phase accompanied by a drop in G1. The sub-G1 population was elevated after 72 h of treatment with either VNK-572 or cisplatin in both cell lines compared to control (e.g., in the HeLa cell line, 5% to 12% for VNK572 (*p* = 0.03) and 6% to 25% for cisplatin (*p* = 0.02). Both compounds observed greater changes in sub-G1 levels in the DU145 prostate cancer cells, i.e., VNK-572 increased sub-G1 population from 1% to 51% after 72 h (*p* = 0.05), while cisplatin increased sub-G1 cells from 1% to 62% after 72 h (*p* = 0.03) (Figure 3B). The time-dependent accumulation of cells in sub-G1 indicates that the cells are undergoing apoptosis/necrosis.

### 2.6 Mechanism of action—apoptosis assay

To further analyze the cytotoxicity mechanisms, annexin V was used to determine whether VNK-572/cisplatin exerts an anti-cancer activity through apoptosis.

**FIGURE 4:**
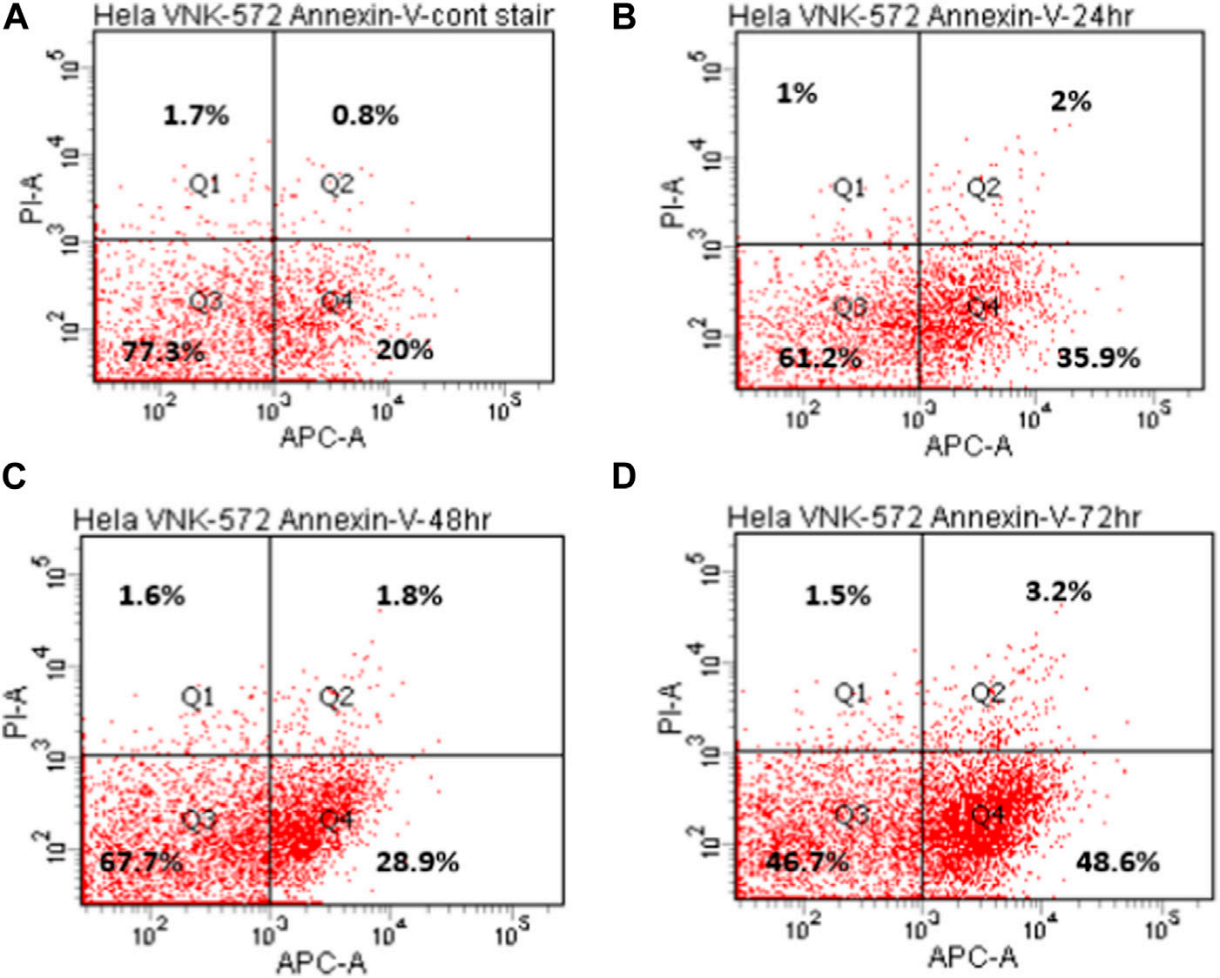
Representative annexin V scatter plots of HeLa cells after treatment with VNK-572 (50 μM). **(A)** 72 h (control), **(B)** 24 h, **(C)** 48 h, and **(D)** 72 h. Q1 and Q2 are cells stained with propidium iodide, indicating necrosis or late apoptosis; Q3 indicates normal cells; and Q4 are cells stained with annexin V- APC dye, and represent early apoptotic cells.

**FIGURE 5:**
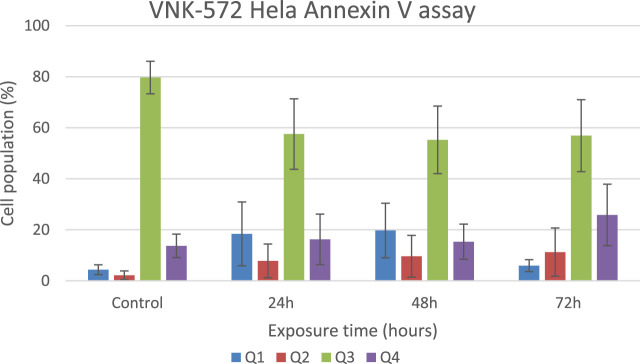
Bar chart of HeLa cell populations in different quarters of the plot after treatment with VNK-572 (50 μM) for control, 24, 48, and 72 h. Data are expressed as mean ± SEM.

The results (Figures 4, 5, and Supplementary Material S6) show that the majority of cells (>70%) are in Q3 (live) before treatment with either compound. An increase in Q4 (annexin V APC), demonstrating early apoptosis, was seen for both VNK-572 and cisplatin following 72 h of treatment (14% vs. 26% for VNK-572% and 13% vs. 39% for cisplatin). The same progressive pattern was shown for Q2 (propidium iodide and annexin V APC-stained cells), representing late-stage apoptosis; a comparable increase was seen with VNK-572 and cisplatin-treated cells following 72 h of treatment (2% vs. 11% for VNK 572% and 2% vs. 9% for cisplatin). It was observed that <6% of cells were in Q1 (which represents necrosis) after 72 h of treatment with either VNK-572 or cisplatin.

These data support the finding of sub-G1 accumulation in Figure 3, which is due to cells entering either apoptosis or necrosis.

### 2.7 DNA binding

**FIGURE 6:**
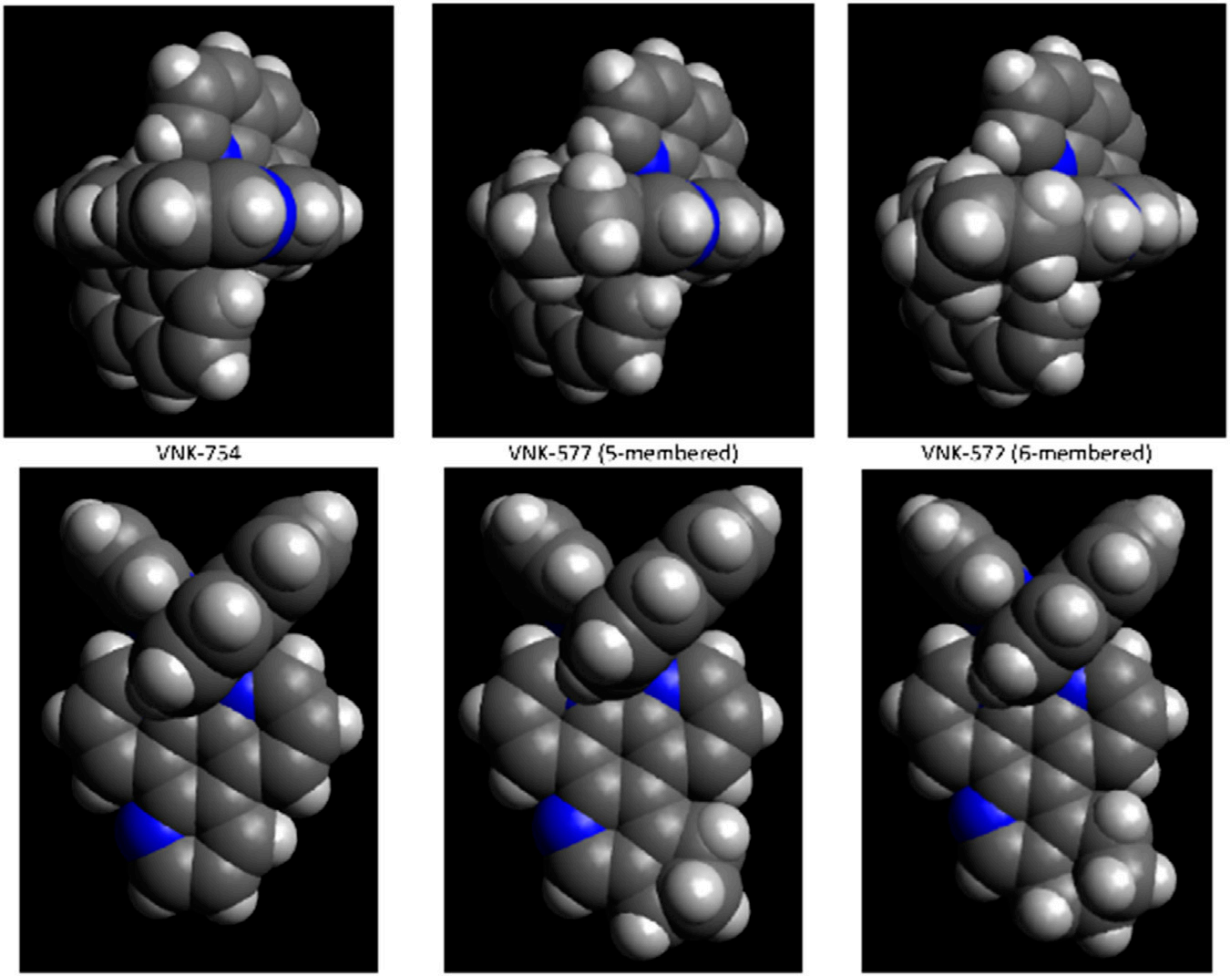
Energy-minimized structures for VNK-754, POW12A, and VNK-572, calculated in Avogadro.

**VNK-754** is a structural analog of [Ru(phen)_2_dpq]^2+^, a complex that intercalates the dpq (dpq = dipyrido[3,2-*f*:2′,3′-*h*]quinoxaline) ligand from the minor groove (Greguric et al., 1997). The dpq ligand presents a sufficiently large planar surface to allow classical intercalation, and it might be expected that extending the surface with substituents would enhance such binding. However, while this is observed with the addition of aromatic rings, as in the case of [Ru(phen)_2_dppz]^2+^, this may not be true for the addition of a saturated ring that distorts the planarity of the extended ring system (Figure 6). Studies were thus undertaken to establish the binding modes of POW-12A and VNK-572. All complexes bound strongly to DNA, and binding constants and binding site sizes were determined from emission titrations to be 1.91 × 10^6^ M^–1^ and 0.38 Ru/base for POW-12A, and 1.46 × 10^6^ M^–1^ and 0.24 Ru/base for VNK-572 (Table 3). VNK-572 showed a binding site size of 4.16 bases; this is consistent with binding via intercalation. The smallest binding site size a pure intercalator can have is four bases. The binding site size of POW-12A is smaller than four bases; this is not consistent with binding via pure intercalation. This is in agreement with data from viscometry (Figure 9) and LD experiments (Figure 8) that suggest the binding mode of POW-12A differs from that of the other compounds, all of which appear to intercalate.

**TABLE 3:** Direct fitting to raw data from fluorescence titration experiments (titrating concentrated HS DNA into 5 μM solutions of ruthenium complex) was used to give the binding constant (KB) and binding site size (n′).

Compound	K_B_ (×10^6^ M^-1^)	n′
VNK-572	1.46	4.16
POW-12A	1.91	2.63

This table shows the binding constant (K_B_) and binding site size (n′- in bases per compound).

Intercalation is typically characterized by the orientation of the extended ligand parallel to the base pairs, duplex unwinding, and lengthening of the DNA. Linear dichroism spectroscopy, supercoiled DNA unwinding assays, and viscometry have been applied to explore whether the cyclopentane and cyclohexane substituents affect the intercalative ability of the complex.

**FIGURE 7:**
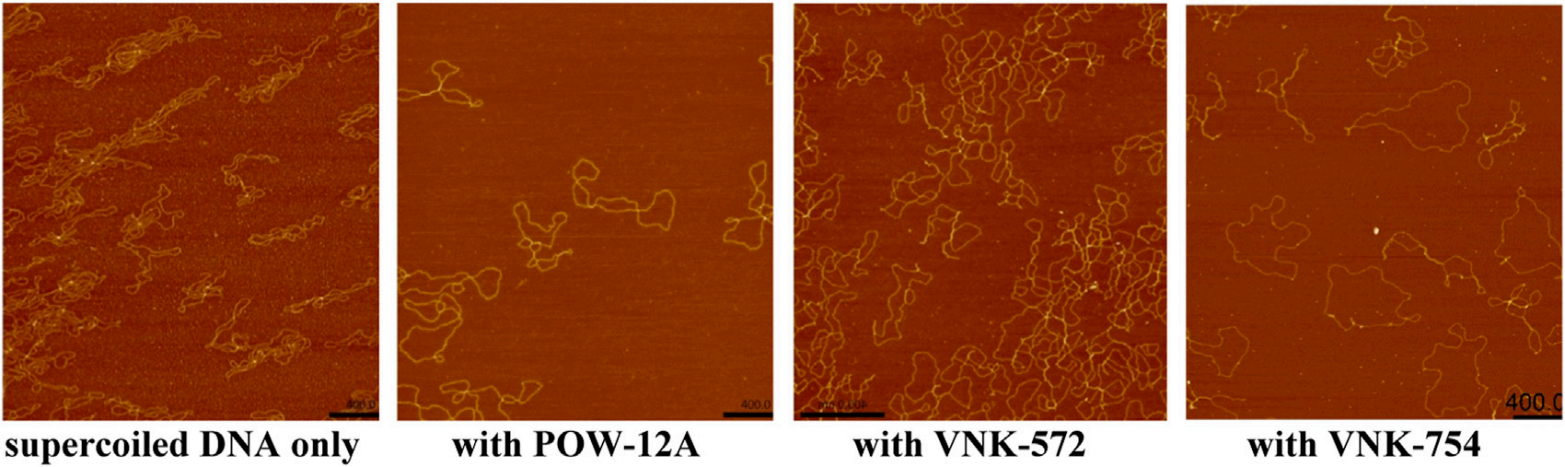
AFM height images RF I φX174 on a magnesium-soaked mica surface over an area of 2 µm. Samples with the ruthenium complex had solution mixing ratios of P/D = 14 for POW-12A and VNK-572; and of P/D = 5 VNK-754.

Figure 7 shows that supercoiled circular DNA is unwound by all complexes: **POW-12A**, **VNK-572,** and **VNK-754**. **VNK-754** under these conditions shows a mixture of open and supercoiled circles, with around half fully open circles. Fewer cross-over points in the circles were observed for VNK-572 and POW-12A, although fully open circles were not produced in these conditions. Gel mobility assays showed that all three complexes led to open circular forms at lower P/D ratios.

**FIGURE 8:**
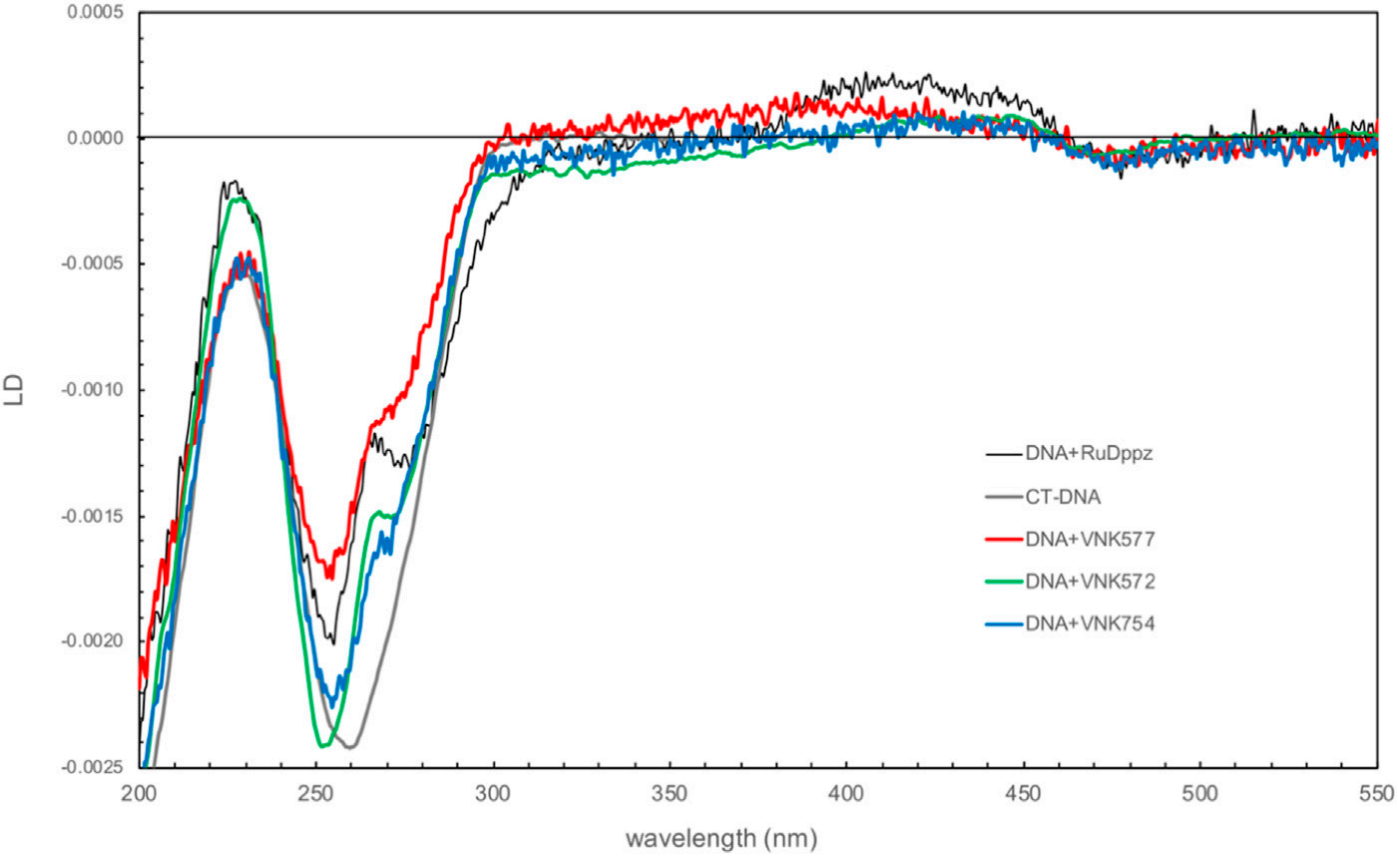
Linear dichroism spectra of VNK-754, POW-12A (VNK-577), and VNK-572 bound to CT-DNA. The spectrum of the strong intercalator [Ru(phen)_2_dppz]^2+^ is shown under the same conditions for comparison. [DNA] = 250 mM; [Ru] = 25 mM; P/D = 10.

Figure 8 shows the comparison of the linear dichroism (LD) spectra of the novel complexes bound to CT-DNA with that of the related compound [Ru(phen)_2_dppz]^2+^, a strongly intercalating complex for which the LD spectrum has been fully characterized in terms of orientation angles with respect to the helix axis (Lincoln et al., 1996). The magnitude of the LD signal in the DNA absorption region reflects how well DNA is aligned in shear flow. The complexes have different effects, with POW-12A causing the greatest reduction in intensity, suggesting that it reduces DNA stiffness or induces structural changes that make DNA more difficult to align. The spectra share features of negative LD at ∼470 nm and positive LD in the 400–450 nm region, and a positive contribution at 265 nm on top of the negative DNA LD. These features are consistent with the dihedral angle between the phen ligands being oriented along the minor groove. In all the spectra except that of **POW-12A**, LD is negative in the 300–350 nm region, where the absorbing transition dipole moments are oriented in the plane of the extended ligand, and analysis indicates that these ligands lie approximately parallel to the base pairs, consistent with intercalation. For the extended ligand of **POW-12A**, the zero-to-positive contribution in this region indicates an average angle of approximately ≤54.7° (the magic angle) with respect to the helix axis. There are two possible explanations: either no complexes are intercalated, or there is a distribution of some intercalated complexes and some that bind the DNA without intercalation. The latter situation would also lead to fewer phen ligands being aligned along the groove, which could also be an implication of the less positive contribution of **POW-12A** at 265 nm. Similar spectra have been observed with [poly(dA-dT)]_2_ and [poly(dG-dC)]_2_, indicating that these are not sequence-selective results.

**FIGURE 9:**
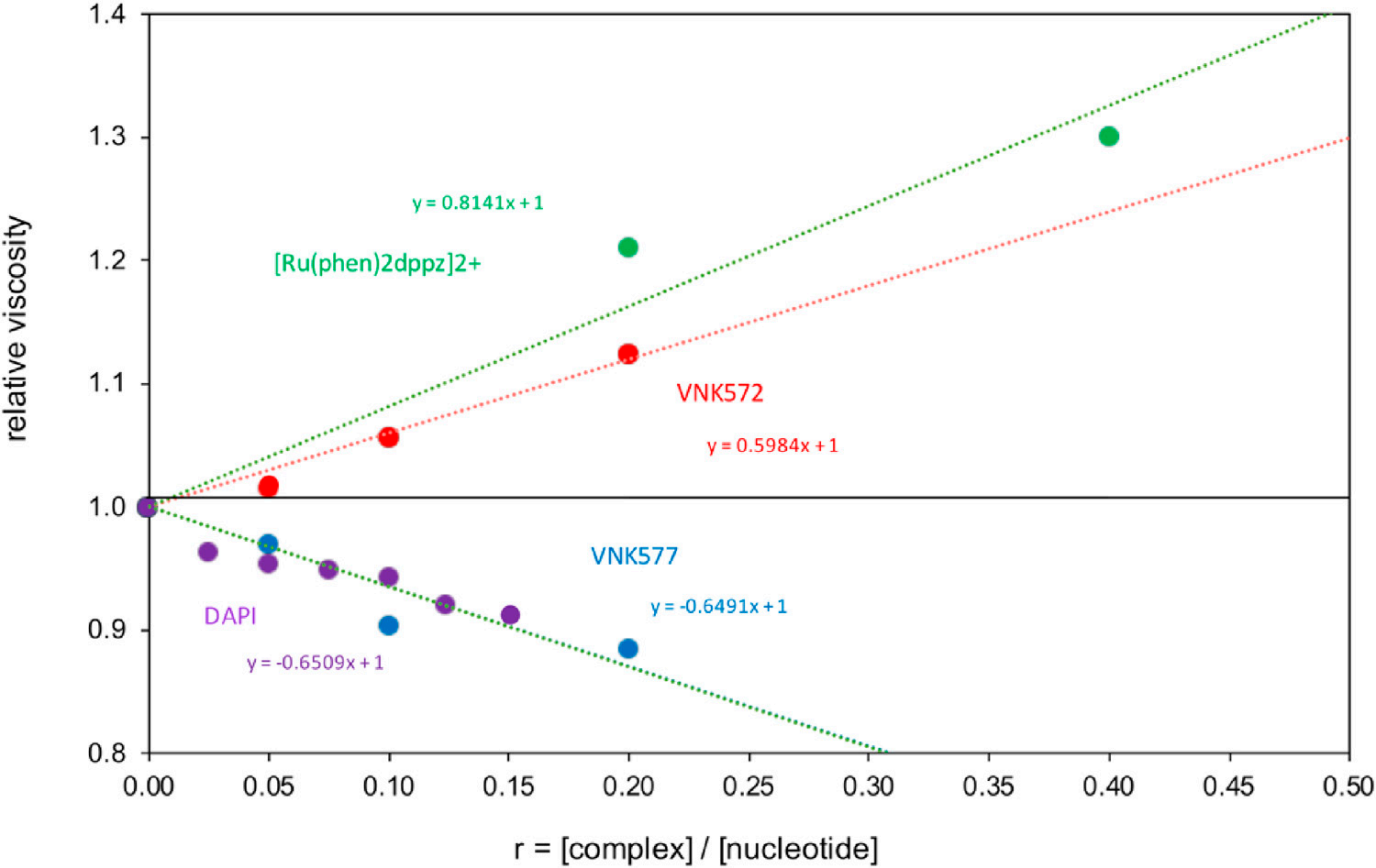
Viscometry of POW12A (VNK-577) and VNK-572 bound to CT-DNA, compared with the standards [Ru(phen)_2_dppz]^2+^ bound to DNA, and DAPI bound to [poly(dA-dT)]_2_.

Viscometry results (Figure 9) show that VNK-572 elongates DNA-like [Ru(phen)_2_dppz]^2+^, consistent with intercalation. POW-12A, by contrast, reduces the viscosity of DNA in a manner similar to the changes observed for the minor groove binder DAPI bound to [poly(dA-dT)]_2_. This reduction reflects increased flexibility of the DNA due to electrostatic and steric factors, which is consistent with the LD results for POW-12A but can also occur if the DNA becomes kinked or bent upon binding a small molecule. Since POW-12A was observed to unwind supercoiled DNA, which does not occur with minor groove binders like DAPI, the similarity of viscosity slopes is likely a coincidence.

Although it seems unlikely from the space-filling models, VNK-572 intercalates DNA better than POW-12A. It is not straightforward to interpret the data in a holistic manner. POW-12A might have a distribution of binding modes, with some molecules interacting by intercalation and others binding in the DNA groove.

## 3 Conclusion

This investigation has demonstrated the cytotoxic effect of the novel ruthenium(II) complexes VNK-572 and POW-12A against cervical, prostate, ovarian, and platinum-resistant ovarian cancer cells. Platinum-based anti-cancer therapies have been limited due to severe dose-limiting side effects and poor selectivity against normal cell lines. The cytotoxic effects of the three ruthenium compounds ranged from the highest in the A2780 ovarian cancer cell line to the lowest in the MDA-MB-231 breast cancer cells. The IC_50_ values were typically an order of magnitude higher than those for cisplatin but crucially showed selective toxicity as, unlike cisplatin, they were less cytotoxic to normal prostate cells (PNT1A) compared to the prostate cancer cell line DU145. This is in line with other studies reporting Ru-complexes to have superior anti-cancer profiles, such as improved selectivity toward cancer cells. Notably, we recorded a seven-fold difference between the cytotoxic activities of VNK-572 and VNK-754 in prostate and ovarian cancer cells. POW-12A displayed similar activity to VNK-572 against all cell lines. Studies showed that nuclear fragmentation and apoptosis were part of the mechanism of action of these complexes, while cisplatin also exhibited necrosis, a much less favorable mode of cell death.

Despite these complexes differing marginally in structure from their parent [Ru(phen)2dpq]2+ (VNK-754), having additional cyclopentane and cyclohexane rings on the extended ligand, a 7.9-fold difference was obtained between the logP of VNK-572 and VNK-754. This increased lipophilicity correlates with the cytotoxicity results and may be a key factor in the observed differences.

All three complexes studied were found to co-localize with DAPI in cell nuclei, suggesting that DNA interactions are important for their action. Cell-free binding studies showed that the complexes bind with micromolar equilibrium constants at physiologically relevant ionic strengths, and the binding mode of **POW-12A** was intercalation, as previously reported for **VNK-754**. **VNK-572**, by contrast, was not a clear intercalator: different biophysical methods showed different behaviors associated with intercalation (supercoiled DNA unwinding) or with minor groove binding (reduced viscosity) or an average of both (in linear dichroism spectra). These results showed no correlation of the cell-free DNA binding mode with the cytotoxicity profile, suggesting that the complexes affect the nuclear machinery, possibly DNA–protein complexes, in different ways. This could arise from their differing lipophilicities or be a result of their different structures causing distortions in a ternary complex.

## 4 Materials and methods

### 4.1 Synthesis

All solvents and reagents were purchased from commercial suppliers and used without further purification. NMR spectra were recorded on a JEOL ECS400FT Delta spectrometer (399.78 MHz for ^1^H NMR). Chemical shifts are reported in parts per million (ppm) relative to a tetramethylsilane internal standard or an NMR solvent peak. Compounds **VNK-497** and **VNK-753** were prepared as described previously (Pabst, G. R.; Pfüller, O. C.; Sauer, J., The new and simple “LEGO” system: synthesis and reactions of ruthenium(II) complexes. Tetrahedron 1999, 55 (26), 8045–8064). Copies of all NMR spectra are available in ESI.

#### 4.1.1 Ligand POW-04


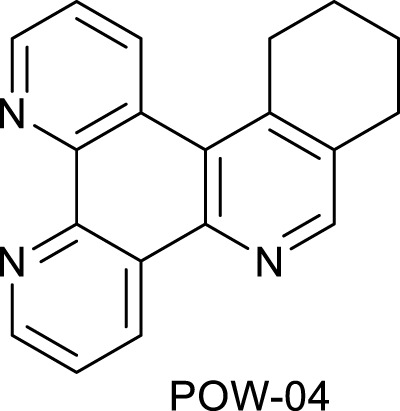


A mixture of the triazine VNK-497 (1.071 g, 4.59 mmol) and 1-morpholinocyclohexene (1.862 g, 10.92 mmol) was stirred at 170°C for 4 h under argon. The mixture was allowed to cool to room temperature. Diethyl ether (15 mL) was added, and the mixture was stirred at RT for 1 h. The formed solid was filtered off and washed with diethyl ether. The product was then purified by column chromatography on silica gel using the dichloromethane/methanol (10:1v/v) mixture as an eluent. The fractions containing the product were combined and allowed to evaporate. The residue was treated with diethyl ether (10 mL). The solid was filtered off and washed with diethyl ether to give the desired product. Yield (0.69 g, 53%). 1H NMR (400 MHz, CDCl3) δ: 9.55 (dd, J = 8.2, 1.8 Hz, 1H), 9.20 (dd, J = 4.6, 1.8 Hz, 1H), 9.17 (dd, J = 4.1, 1.4, 1H), 8.97 (br.d, J = 8.6 Hz, 1H), 8.71 (s, 1H), 7.72 (dd, J = 8.2, 4.6 Hz, 1H), 7.63 (dd, J = 8.5, 4.1 Hz, 1H), 3.45 (t, J = 6.3 Hz, 2H), 3.10 (t, J = 6.3 Hz, 2H), 2.06–1.99 (m, 2H), and 1.93–1.86 (m, 2H).

#### 4.1.2 Ligand POW-05


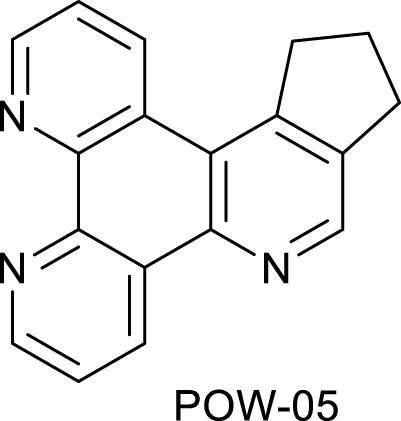


A mixture of triazine **VNK-497** (0.532 g, 2.28 mmol) and 1-morpholinocyclopentene (1.115 g, 7.28 mmol) was stirred at 160°C for 3.5 h. The product was applied to a silica gel column. The column was eluted with the dichloromethane/methanol solution (10:1) mixture. Fractions containing the product were combined and evaporated to dryness. Diethyl ether (5 mL) was added, and the mixture was stirred at room temperature for 1 h and filtered. The solid on the filter was washed with diethyl ether and dried. Yield (0.240 g, 40%).1H NMR (400 MHz, CDCl_3_) δ: 9.6 (dd, J = 8.2, 1.8 Hz, 1H), 9.22 (dd, J = 4.6, 1.8 Hz, 1H), 9.20 (dd, J = 4.6, 1.4 Hz, 1H), 8.96 (s, 1H), 7.74 (dd, J = 8.2, 4.6 Hz, 1H), 7.69 (dd, J = 8.2, 4.6 Hz, 1H), 3.71 (t, J = 7.3 Hz, 2H), 3.21 (t, J = 7.3 Hz, 2H), and 2.36 (quint, J = 7.3 Hz, 2H).

#### 4.1.3 Complex VNK-754


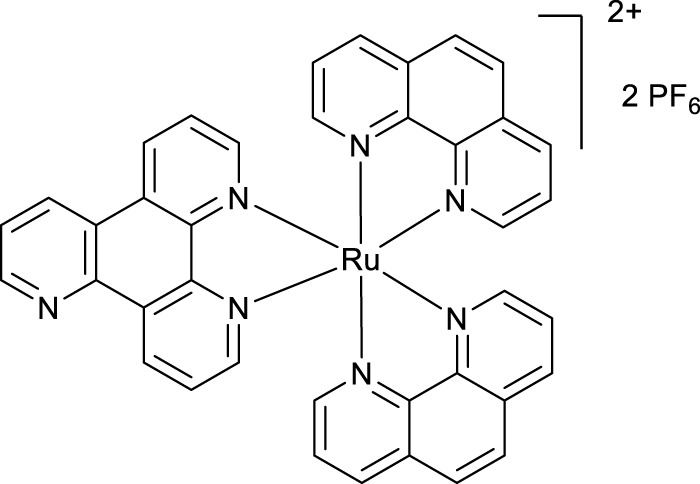


A mixture of phen_2_RuCl_2_ (266 mg, 0.5 mmol) and the ligand **VNK-753** (139 mg, 0.6 mmol) in ethanol/water, 1/1, v/v mixture (20 mL), was heated under reflux for 16 h. The mixture was gravity-filtered and diluted with water (20 mL). The solution was extracted with chloroform (3 × 20 mL) and then with DCM (3 × 20 mL). To the aqueous layer, a solution of sodium hexafluorophosphate (815 mg, 5 mmol) in water (15 mL) was added, and the mixture was again extracted with DCM (3 × 20 mL). The organic layer was dried over MgSO_4_ and gravity-filtered into a round-bottomed flask. Ethanol (20 mL) was added, and the mixture was concentrated to a volume of approximately 10 mL. The formed bright-red solid was filtered off and washed on a filter with 10 mL of ethanol. Yield 325 mg. (66%) 1H NMR (400 MHz, acetonitrile-D3) δ: 9.54 (d, J = 8.2 Hz, 1H), 9.20–9.10 (m, 3H), 8.55 (br.d, J = 8.2 Hz, 4H), 8.21 (s, 4H), 8.10–7.85 (m, 7H), and 7.69–7.56 (m, 6H).

#### 4.1.4 Complex VNK-572


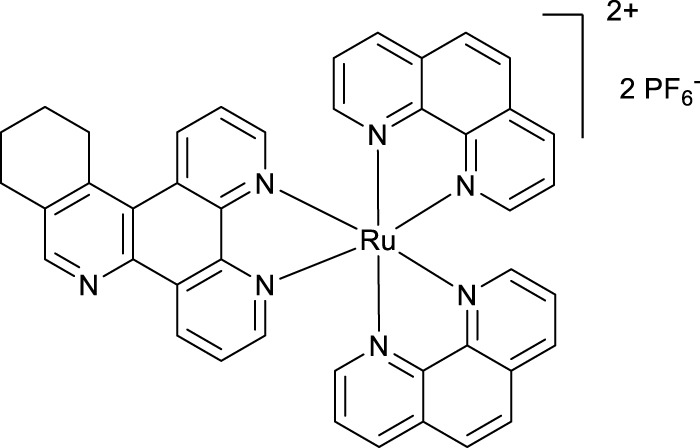


A mixture of phen_2_RuCl_2_ (133 mg, 0.25 mmol) and the ligand VNK-321–2 (73 mg, 0.25 mmol) in the ethanol/water 1/1 vv mixture (8 mL) was irradiated by microwave (200 W) for 20 min, maintaining the temperature at 120°C. The mixture was extracted with DCM (2 × 20 mL) in a separating funnel. To the aqueous layer, a 10% solution of potassium hexafluorophosphate (10 mL) was added, and the mixture was again extracted with DCM (3 × 20 mL). The organic layer was dried over MgSO_4_ and gravity-filtered. Ethanol (10 mL) was added, and the mixture was concentrated to a volume of approximately 5 mL. The red solid was filtered off and washed with ethanol to give the desired product after drying in an oven at 100°C for 30 min. Yield 153 mg (59%). 1H NMR (400 MHz, methanol-D4) δ: 9.64 (d, J = 8.0 Hz, 1H), 9.43 (d, J = 8.0 Hz, 1H), 8.90 (s, 1H), 8.68 (br.d, J = 8.0 Hz, 4H), 8.31 (s, 4H), 8.25–8.08 (m, 6H), 7.79–7.69 (m, 6H), 3.70–3.50 (m, 2H), 3.18 (br.t, J = 7.6 Hz, 2H), and 2.1–1.9 (m, 4H).

#### 4.1.5 Complex POW-12A


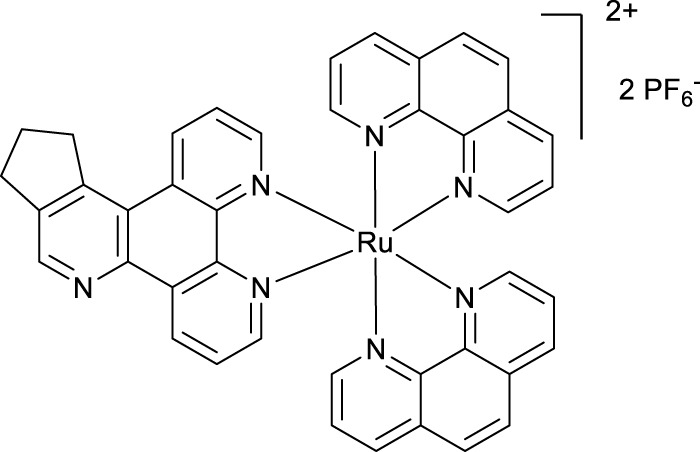


A mixture of phen_2_RuCl_2_ (98 mg, 0.18 mmol) and the ligand **POW-05** (55 mg, 0.20 mmol) in the ethanol/water 1/1 vv mixture (6 mL) was irradiated by microwave (200 W) for 20 min, maintaining the temperature at 120°C. A saturated aqueous solution of potassium hexafluorophosphate (10 mL) was added, resulting in the formation of a fine solid. The solid was filtered off and washed with water (20 mL) to give the desired product after drying in an oven at 100°C for 4 h. Yield 154 mg. 1H NMR (400 MHz, Methanol-D4) δ: 9.58 (d, J = 8.0 Hz, 1H), 9.18 (d, J = 8.0 Hz, 1H), 9.05 (s, 1H), 8.57 (br.d, J = 8.0 Hz, 4H), 8.23 (s, 4H), 7.68–7.57 (m, 6H), 3.82–3.69 (m, 2H), 3.24 (t, J = 7.8 Hz, 2H), and 2.42–2,32 (m, 2H).

### 4.2 *In vitro* cytotoxicity assay

The sulphorhodamine B (SRB) assay was used to evaluate the cytotoxicity of each cancer line after treating them with the metal complexes in line with the previously established protocol (Skehan et al., 1990). A cell density of 3 × 10⁴ cells/mL was treated with various concentrations (3, 10, 30, 50, and 100 μΜ) of each ruthenium complex by 1:200 dilutions of stock solutions (in DMSO) in culture media, using DMSO as a control. Then, 100 μL of the cell–drug suspension was added to the respective wells of a 96-well plate and incubated at 37°C for 72 h in a CO_2_-containing incubator. Following this, the cells were fixed using 25 ul of Carnoy’s solution and left in the fridge (4°C) for 1 h. The plates were then rinsed with running water and tapped on paper to remove dead cells. The cells were then stained with 100 μL of sulphorhodamine B dye and left for 30 min, after which the dye was washed off using 1% acetic acid, and the plate was left to dry in the oven (60°C). The stained cells were then treated with 100 μL of Tris base (10 mM, pH 10) to solubilize the dye stain. Finally, the plates were analyzed using an Omega plate reader. Each assay was conducted in triplicate.

$$\%\mathrm{Cell response}=\left(\mathrm{Average of treated cells}/\mathrm{Average of control cells}\right)\;x\;100.$$

### 4.3 Cell viability assay

This test was carried out using a previously published method (Strober, 2001). A cell density of 3 × 10⁴ cells/mL was treated with varied concentrations of each complex (final concentrations—3, 10, 30, 50, and 100 μΜ). These were then transferred to a 6-well plate, each well containing 2 mL of the cell–drug suspension. The plates were then incubated at 37°C for 72 h. Thereafter, the cell suspension in each well was discarded into 1% virkon, and the wells were rinsed with 500 μL of phosphate saline buffer (PBS), after which the cells were trypsinized with 500 μL of trypsin. Afterward, 500 μL of culture media was used to neutralize the effect of trypsin. Using a ratio of 1:1, 10 μL of cell suspension was mixed with 10 μL of trypan blue. The mixture was pipetted under a cover slip mounted on a hemocytometer, and the viable cells were counted using a light microscope at a magnification of ×100. The four boxes of the hemocytometer were counted, and the value was divided by 4. This was then multiplied by 2, which was used to determine the trypan blue dilution factor. This assay was also conducted in triplicate.

$$\%\mathrm{Cell viability}=\left(\mathrm{Average of treated cells}/\mathrm{Average of control cells}\right)\;x\;100.$$

### 4.4 Epi-fluorescence nuclear staining

To assess the nuclear morphological changes caused by ruthenium complexes on the cell lines, the cells were stained using 4′,6-diamidino-2-phenylindole DAPI dye and analyzed under an EVOS epi-fluorescence microscope at a magnification of ×60 (Lewandowska et al., 2013). The protocol involves treating the cells with the complexes, culturing them in 6-well plates, and incubating them at 37°C for 72 h. The cells were then rinsed with PBS, trypsinized, and neutralized using culture media. The cells were then spun in a centrifuge at 2500 RPM for 3 min, and the supernatant was discarded. The cell pellet was then fixed with 15 μL of Carnoy’s solution, resuspended, and spread on a microscope slide. When the cells were dried, 2–3 drops of DAPI were dropped on the smear in the dark and covered with a slip. The slides were then viewed under the epi-fluorescence microscope, and the nuclei were characterized qualitatively. A total of 30 cells per slide were counted, and the number of cells with various nuclear morphologies was recorded. Cells were then classed as normal, chromatin-condensed, swollen, nuclear-condensed, and nuclear-fragmented cells based on their nuclear morphology.

### 4.5 UV absorption spectroscopy

The absorption spectra of the complexes were obtained using a Varian UV-VIS or a Shimadzu UV-1800 spectrophotometer in quartz cuvettes with path lengths from 1 to 10 mm, with a buffer baseline. All absorbance spectra are scaled to a 1-cm path length. Concentrations of stock solutions were determined spectrophotometrically using known extinction coefficients.

### 4.6 Emission spectroscopy

The emission spectra were recorded using a FluoroMax-2 or FluoroMax-4 spectrofluorometer (Horiba Jobin Yvon, Inc., France) with excitation at 450 nm. The slit width was set at 2 mm. Spectra are corrected for lamp intensity and detector response.

### 4.7 *In vitro* fluorescence evaluation

Fluorescence microscopy was used to study cellular localization. HeLa cells were seeded on autoclaved coverslips in the 6-well plate and incubated at 37°C for 48 h, after which the culture media were replaced with media containing VNK-572 (50 μM) and VNK-754 (50 μM), respectively, and further incubation was performed for 2 h. Following this, the cells were fixed with Carnoy’s solution and washed twice with PBS thereafter. Finally, the HeLa cells were counterstained with DAPI, and the seeded coverslips were inverted and placed on slides for examination. A Leica confocal microscope was used for the observation of the samples using a magnification of ×63.

### 4.8 Shake-flask method

To study the lipophilicity of our complexes, we used the shake-flask method at 25.0ºC ± 0.1°C in line with the previously reported protocol (Dubey et al., 2017). Equal volumes of the aqueous phase (phosphate buffered saline, pH 7.4) and the 1-octanol phase were pre-saturated with each other overnight. A known amount of the complexes (respectively) was added to the aqueous phase, ensuring our final concentration was within the range of UV spectroscopic analysis; then, an equal volume of the pre-saturated ethanol phase was added. Both phases were then mixed in a conical flask and left shaking on a thermostatic shaker at a speed of 100 rpm overnight at room temperature. The mixture was then left to stand for 24 h to allow for phase separation. Considering that lipophilic compounds will partition into the octanol phase, the concentration of the complexes left in the aqueous phase was analyzed, respectively, by UV spectroscopy; then, the Log P value was calculated: Logp = Log10 ([solute] un-ionized octanol/[solute] un-ionized water).

### 4.9 Annexin V assay

The Annexin V assay was performed following the established procedure. The steps included treating the cells with the complex for 24, 48, and 72 h in 6-well plates and incubating at 37°C. The cells were then rinsed with PBS, trypsinized, and neutralized using culture media. The cells were then spun in the centrifuge at 2500 RPM for 3 min, and the supernatant was discarded. The cell pellet was then washed first with PBS and then with ×1 binding buffer. The cells were then suspended in approximately 1 mL of binding buffer (1–5 million cells), and approximately 100 ul of this suspension was stained with 5 μL of annexin V-APC dye for 15 min. The cells were then washed with binding buffer and resuspended in 200 μL of binding buffer before being stained with 5 ul of propidium iodide. The cells were then analyzed using a flow cytometer within 4 h.

### 4.10 Materials for binding studies

PBS buffer was prepared with NaCl (137 mM), KCl (2.70 mM), Na_2_HPO_4_ (10.1 mM), and KH_2_PO_4_ (1.77 mM) in nanopure water. Using HCl, the pH was adjusted to 7.40. PBS buffer was used for all photophysical experiments. For linear dichroism experiments, a PBS buffer without potassium was used. Solutions of open-circle and supercoiled φX174 DNA were obtained from New England Biolabs. Sodium salts of high-molecular-weight herring sperm (HS-DNA) and calf thymus (CT-DNA) from Sigma were dissolved in PBS buffer, and concentrations were determined spectrophotometrically using the extinction coefficient ε_260 nm_ = 6600 M^−1^ cm^−1^ for nucleobase concentration. The alternating polynucleotide polydeoxyadenylic-thymidylic acid ([poly(dA-dT)]_2_) and polydeoxyguanylic-cytidylic acid ([poly(dG-dC)]_2_) from NUNABIO (Whitfield et al., 2015) were dissolved in PBS buffer, and their concentrations were determined using the extinction coefficients ε_262 nm_ = 6700 M^−1^ cm^−1^ and ε_254 nm_ = 8400 M^−1^ cm^−1^, respectively. Direct fitting to raw data from fluorescence titration experiments (titrating concentrated HS DNA into 5 μM solutions of ruthenium complex) was used to give the binding constant (K_B_) and binding site size (n′). The following equation is derived from the Scatchard equation: $\frac{\propto^{2}∙K_{B}}{PD}-\left(\frac{K_{B}}{PD}+\frac{1}{PD\left[D_{t}\right]}+K_{B}∙n^{\prime }\right)+K_{B}∙n^{\prime }$ = 0, where $\frac{\left[D_{b}\right]}{\left[D_{t}\right]}=\frac{I-I_{0}}{I_{\infty }-I_{0}}$ , PD is the phosphate: dye ratio, K_B_ is the binding constant (M^−1^), and n′ is the binding site size (bases per compound). The equation is in the form of a quadratic equation, where a = K_B_[D_t_]/PD, b = −(K_B_/PD+1/PD[D_t_ ] +K_B_∙n′), and c = K_B_n′. The positive solution to this quadratic equation gives values for α, from which theoretical intensities at each PD are calculated. The least squares regression method was used, minimizing the squared residuals of the theoretical and experimental intensities and floating the values of K_B_, I_0_, I_∞_, and n′.

### 4.11 Atomic force microscopy

Samples for atomic force microscopy (AFM) were prepared on mica crystals cut into squares of approximately 1 cm^2^. These squares were stuck with double-sided tape to a 15-mm diameter metal disc. Before sample preparation, Cellotape was used to remove the top layer of the mica crystal, giving a freshly cleaved surface layer on which the sample is to be deposited. Mica was soaked in a 0.1 M solution of MgCl_2_ for 5 min and blown dry, and Mg^2+^ was used to help DNA (negatively charged) adhere to the negatively charged surface of the mica. For solutions containing DNA and POW-12A or VNK-572, a P/D value of 14 was used. For the solution containing DNA and VNK-754, a P/D value of 5 was used. Then, 2 μL of the solution was pipetted onto the mica surface and left for 10 min until the solvent had evaporated, leaving the DNA adhered to the surface. To remove excess salt, each sample was washed three times with 10 µL nanopure water, drying with nitrogen after each wash. AFM was conducted using a Bruker Multi-mode 2 atomic force microscope operated with a NanoScope V controller. ScanAsyst-AIR AFM tips were purchased from Bruker. Scans were run in tapping mode using ScanAsyst, measuring height, peak force error, and phase. The spring constant was adjusted to the spring constant of the tip, which is 0.4 N m^−1^ for ScanAsyst-AIR tips; feedback parameters were set automatically by ScanAsyst Auto. Scans were run over 2 μm^2^ areas with 512 lines. Images were prepared using NanoScope analysis software. Raw data were processed using first-order flattening and third-order plane fitting. If the background required smoothening due to large molecules on the surface, first-order flattening with a threshold of Z < 0.05 nm was used to improve the appearance of the background. Horizontal streaking was removed using the erase function to remove individual lines, or the clean image function with streak removal was implemented. A brown color scale between −3 and +3 nm was used on all images.

### 4.12 Linear dichroism

LD experiments (Nordén et al., 2010) were run using a Jasco J-810 Circular Dichroism SpectroPolarimeter fitted with a CF-573 Couette flow cell unit. The cell used has a pathlength of 1 mm (2 × 0.5 mm) and a 100 μL volume; the Couette cell consisted of an outer quartz cylinder and an inner quartz rod. A shear gradient was generated by the rotation of the inner quartz glass rod, and the DNA alignment is at a tangent to the direction of flow. The LD spectra of all samples were measured at 4,080 rpm to give the maximum LD signal and also at 0 rpm to give a background. All samples were subsequently zeroed and baselined by subtracting LD at 0 rpm from that at 4,080 rpm. A scan speed of 100 nm/min, response time of 0.5 s, 0.5 nm step, and 1 nm bandwidth were used.

### 4.13 Viscometry

An uncalibrated Cannon-Manning extra-low charge semi-micro viscometer (size 75) was used to measure the relative intrinsic viscosity of dilute DNA solutions, according to the manufacturer’s instructions (Cannon Instrument Company) and as described previously (MoradpourHafshejani et al., 2013). The viscometer was immersed in a water bath thermostated at 25°C. The DNA concentration and viscometer volume (300 mL) were kept constant for a series of added dye concentrations. The flow time for water was ∼177 s, and that for DNA solutions was >245 s. Measurements were carried out in triplicate and provided standard deviations of <±1 s. The flow times are related to the relative intrinsic viscosity according to Eq. 1, where [η] is the intrinsic viscosity in the presence of dye and [η]_0_ is the intrinsic viscosity of free DNA. t_0_, t_d_, and t_x_ represent the flow times for buffer only, naked DNA, and dye-bound DNA, respectively. For long DNA in a random coil conformation, if the persistence length does not change on intercalation (i.e., at low binding ratios), a plot of the cube root of the relative intrinsic viscosity against the binding ratio (*r* = [complex]/[DNA]) yields a slope of 1.4. If the persistence length is reduced or the DNA is bent (dynamically or statically), the slope will be lowered.

$$\sqrt[{3}]{\frac{t_{x}-t_{t}}{t_{d}-t_{t}}}=\sqrt[{3}]{\frac{\left[\eta \right]}{{\left[\eta \right]}_{0}}}=\frac{L}{L_{0}}=1+\alpha r.$$ (1)

## Data Availability

The original contributions presented in the study are included in the article/Supplementary Material; further inquiries can be directed to the corresponding authors.
