# Supplementary material for: Effect of cyclic substituents on the anti-cancer activity and DNA interaction of ruthenium(II) bis-phenanthroline dipyridoquinoline
Source: Front Mol Biosci. 2023 Oct 18;10:1252285. doi: 10.3389/fmolb.2023.1252285 (PMC10619691; doi:10.3389/fmolb.2023.1252285)
Supplement: Supplementary file 1 [file DataSheet1.pdf]

Supplementary Material

Effect of cyclic substituents on the anti-cancer activity and DNA interaction of ruthenium(II) bis-phenanthroline dipyridoquinoline

Etubonesi E Nyong-Bassey,[1] Andrew L. Hicks,[2] Poppy Bergin,[1] Eimer M. Tuite,[2*] Valery Kozhevnikov,[1*] Stephany Veuger[1*]

*** Correspondence:**
Corresponding Author
s.veuger@northumbria.ac.uk; eimer.tuite@ncl.ac.uk; valery.kozhevnikov@northumbria.ac.uk

Cytotoxicity activities of VNK-572, VNK-754 and POW-12A

S1: S1A, S1B, S1C and S1D

S2: S2A, S2B, S2C and S2D

S3: S3A, S3B, S3C and S3D

S4: S4A, S4B, S4C and S4D

S5: S5A1, S5A2, S5A3, S5B1, S5A2, S5B3, S5C1, S5C2 and S5C3

S6: S6A and S6B

S7: S7A and S7B

Synthesis

NMR

S1: Sulphorhadamine B (SRB) assay (Cytotoxicity assay)

S1A.

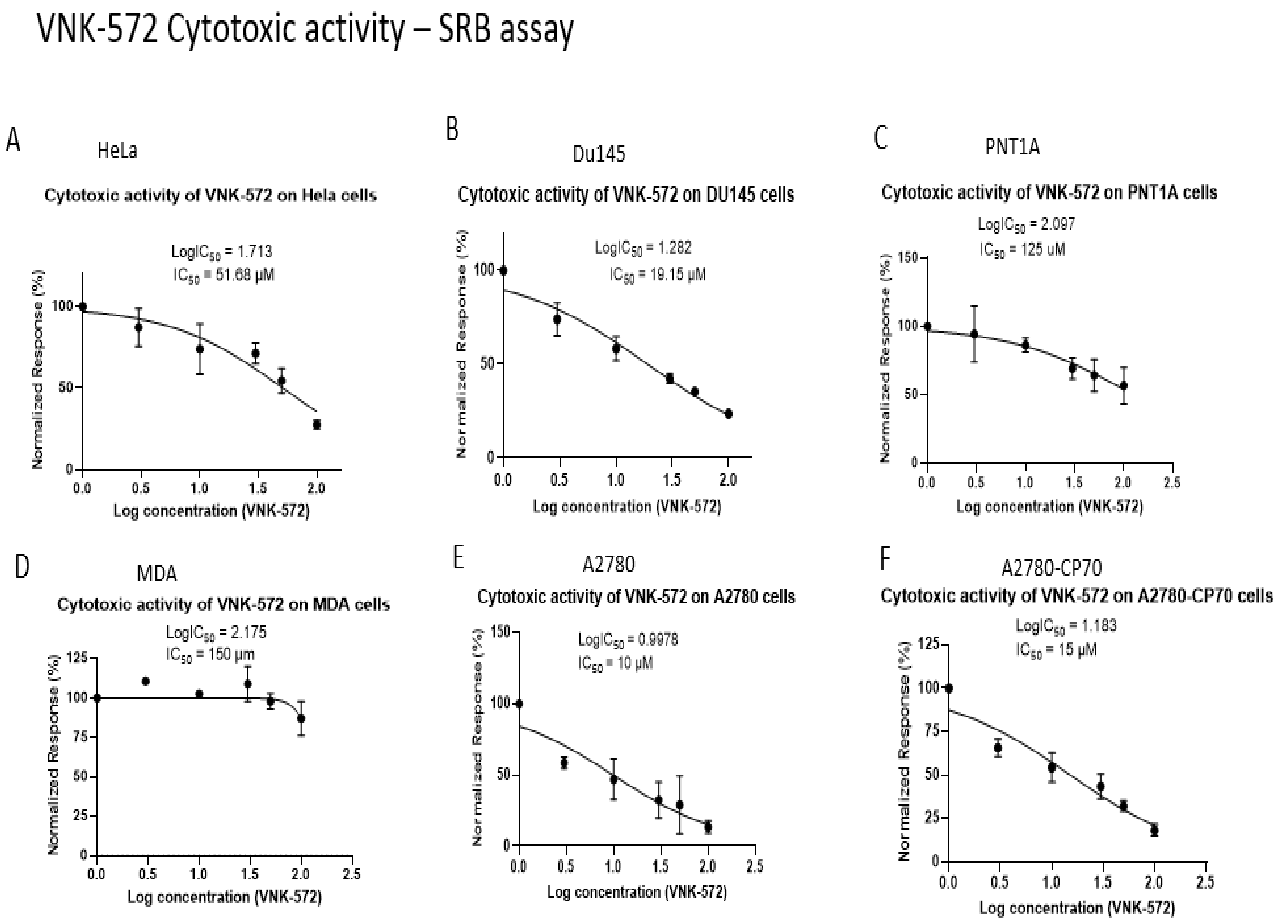

Figure S1A: SRB assay results for VNK-572 on various cell lines

S1B

VNK-754 Cytotoxic activity – SRB assay

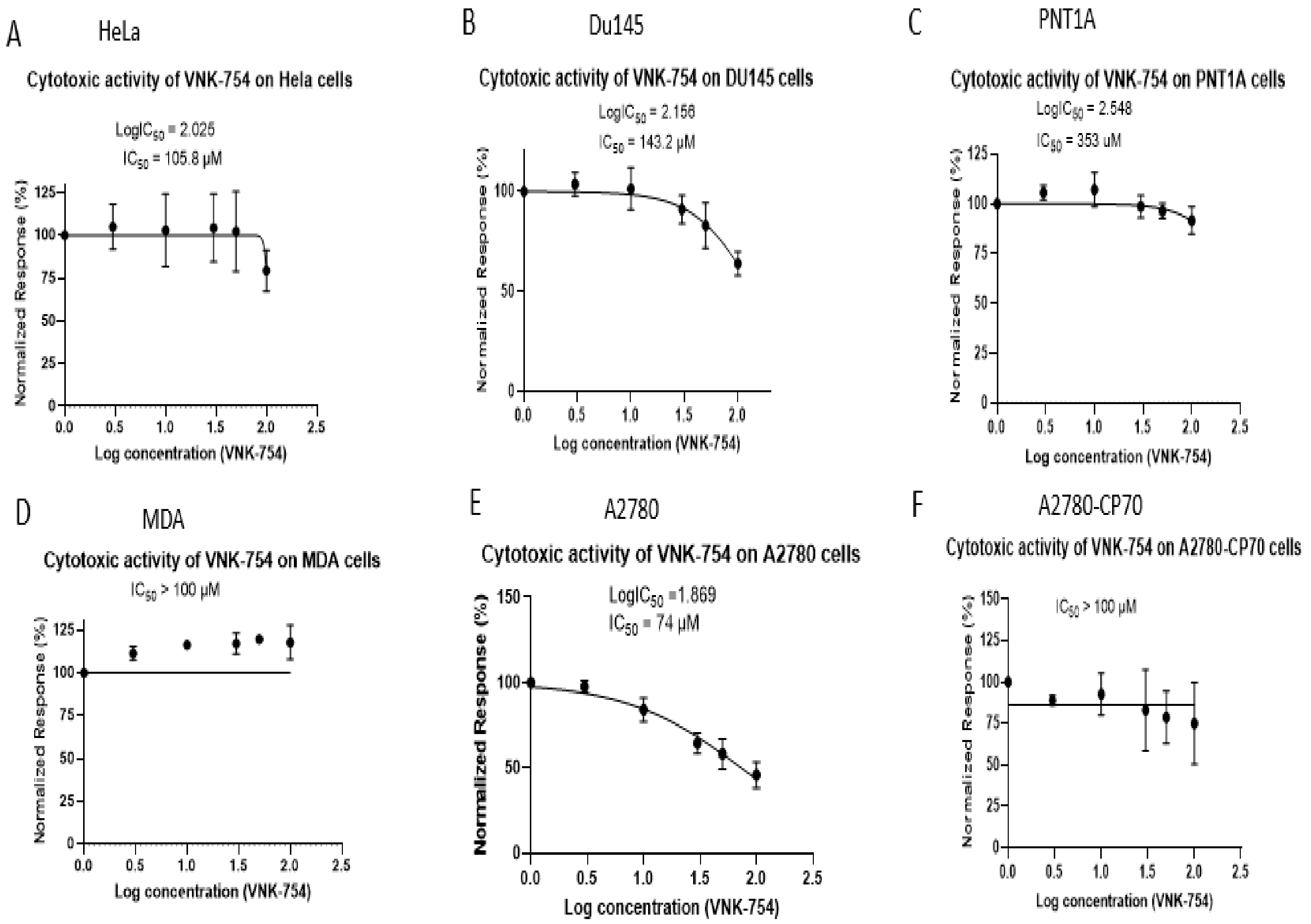

Figure S1B: SRB assay results for VNK-752 on various cell lines.

S1C

POW-12A Cytotoxic activity – SRB assay

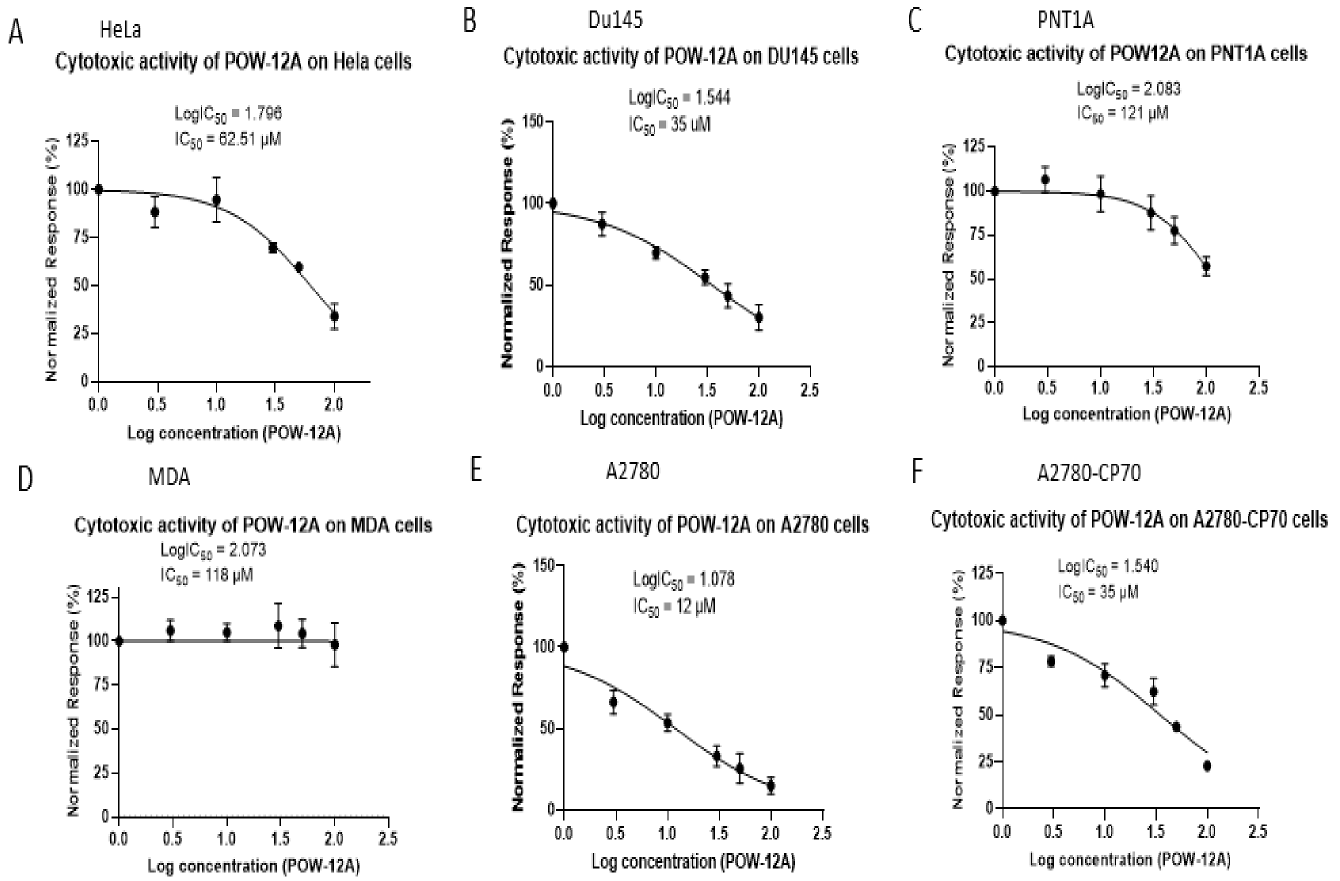

Figure S1C: SRB assay results for POW-12A on various cell lines

S1D

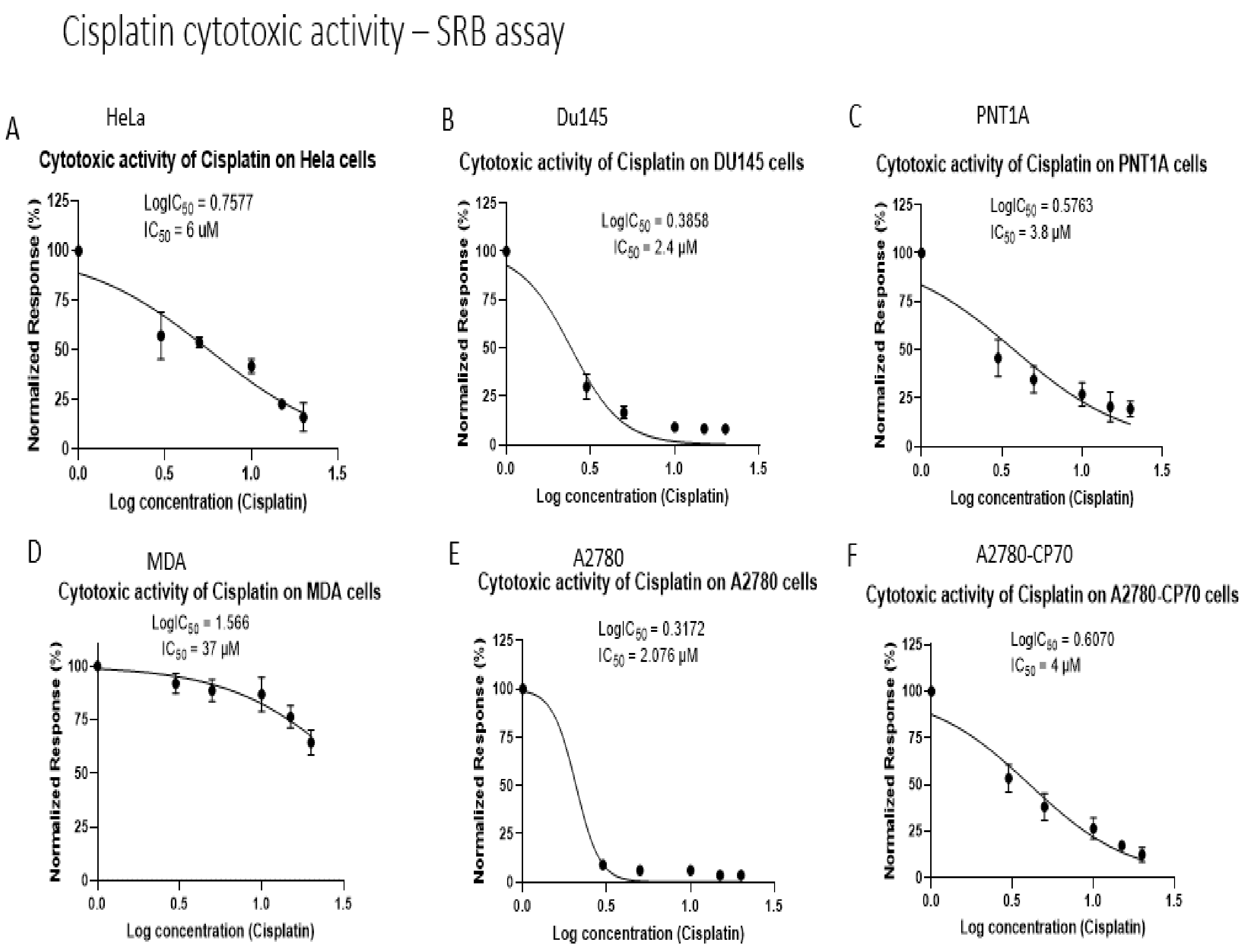

Figure S1D: SRB assay results for Cisplatin on various cell lines

S2 : Trypan blue assay (Cell viability)

S2A

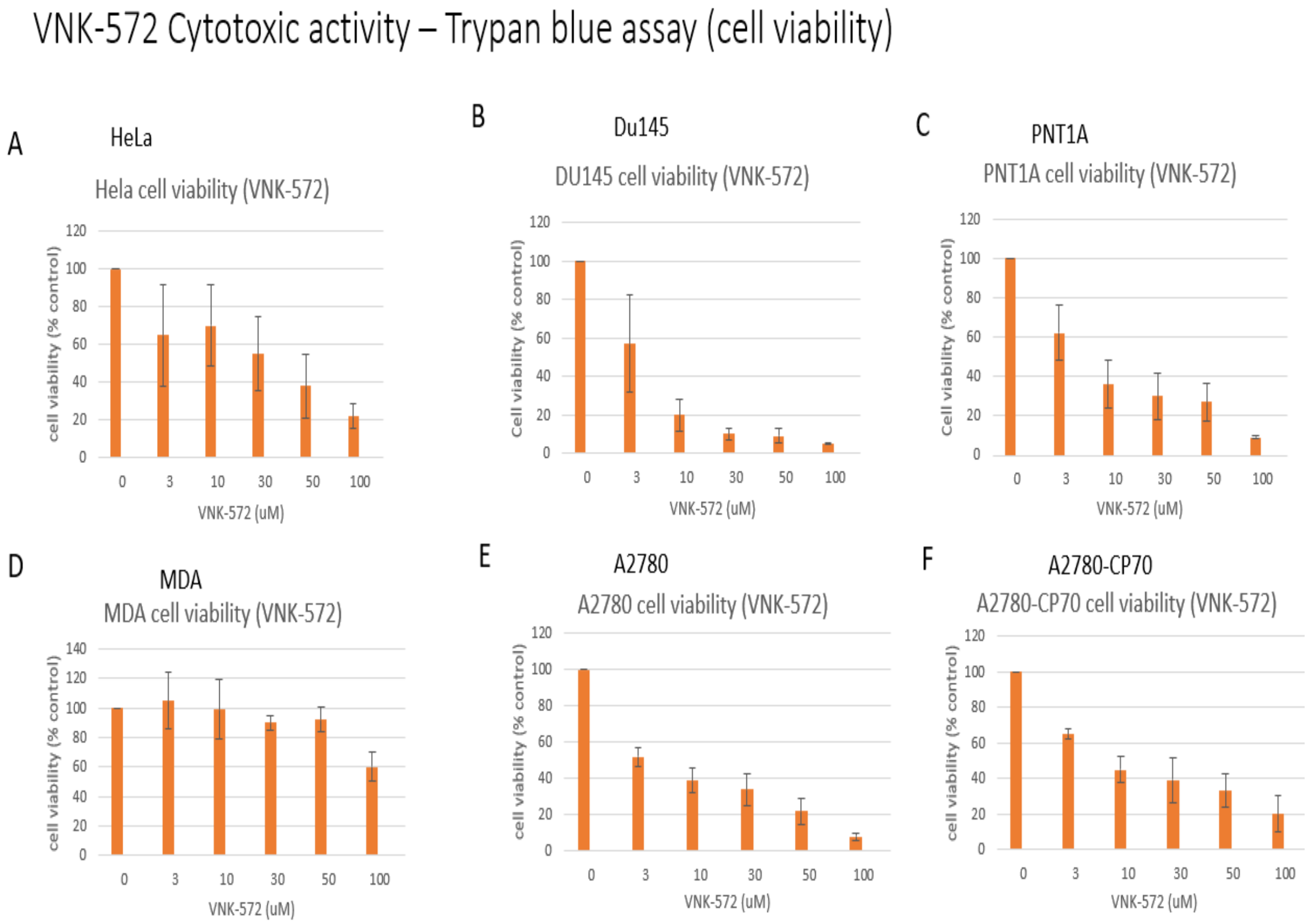

Figure S2A: Trypan blue assay results for VNK-572 treatment on various cell lines. Error was calculated using Standard error of the mean (SEM).

S2B

VNK-754 Cytotoxic activity – Trypan blue assay (Cell viability)

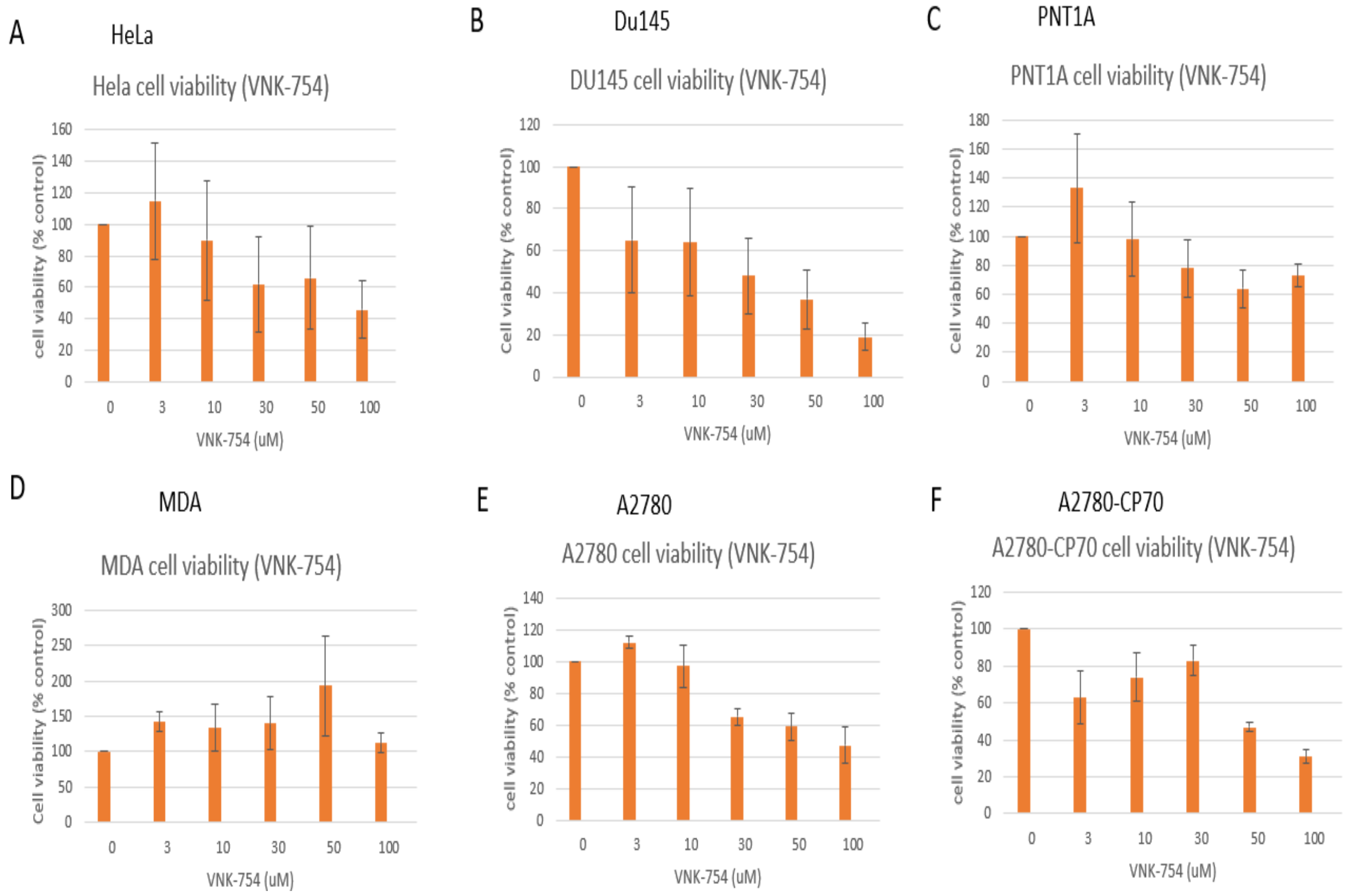

Figure S2B: Trypan blue assay results for VNK-754 treatment on various cell lines. Error was calculated using Standard error of the mean (SEM).

S2C

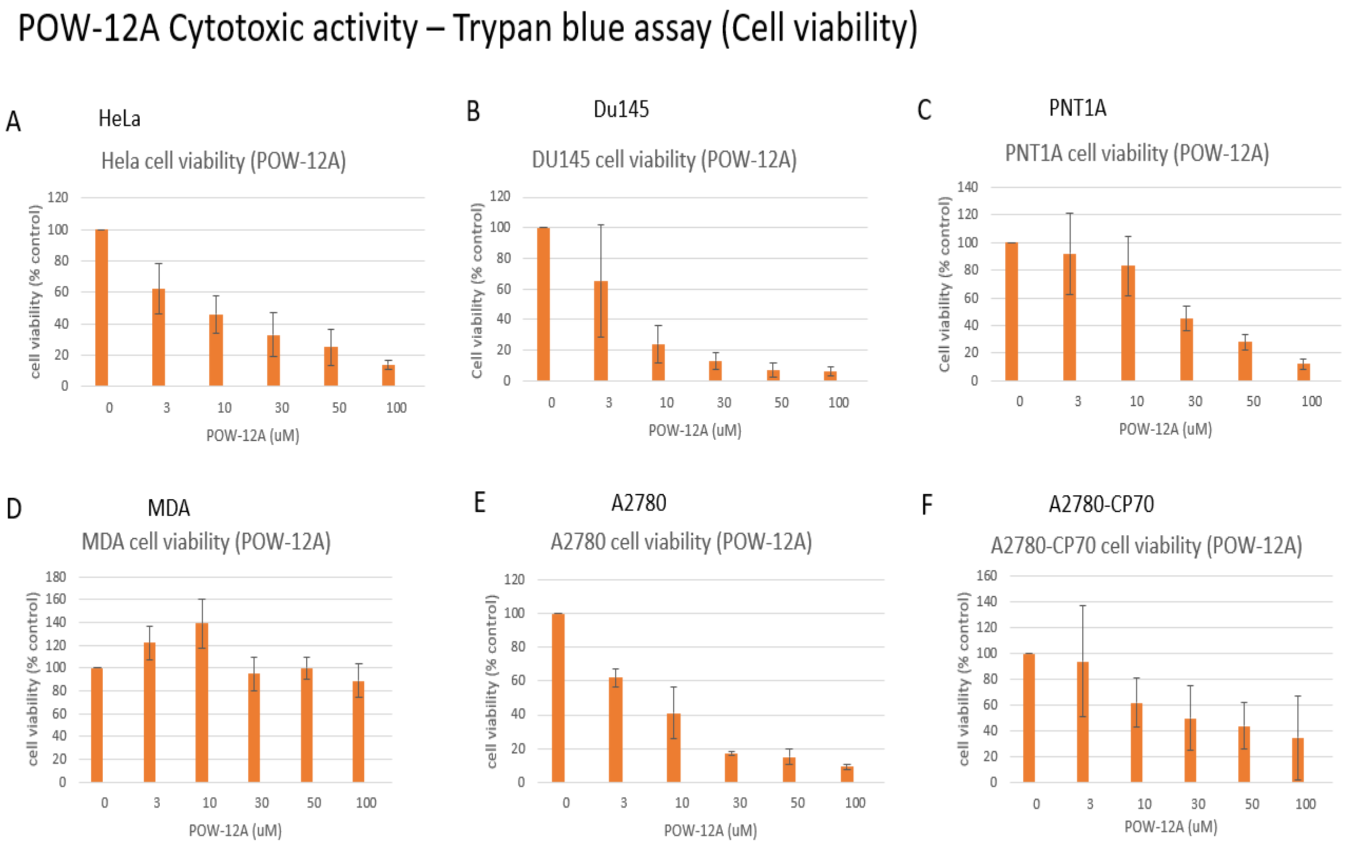

Figure S2C: Trypan blue assay results for POW-12A treatment on various cell lines. Error was calculated using Standard error of the mean (SEM).

S2D

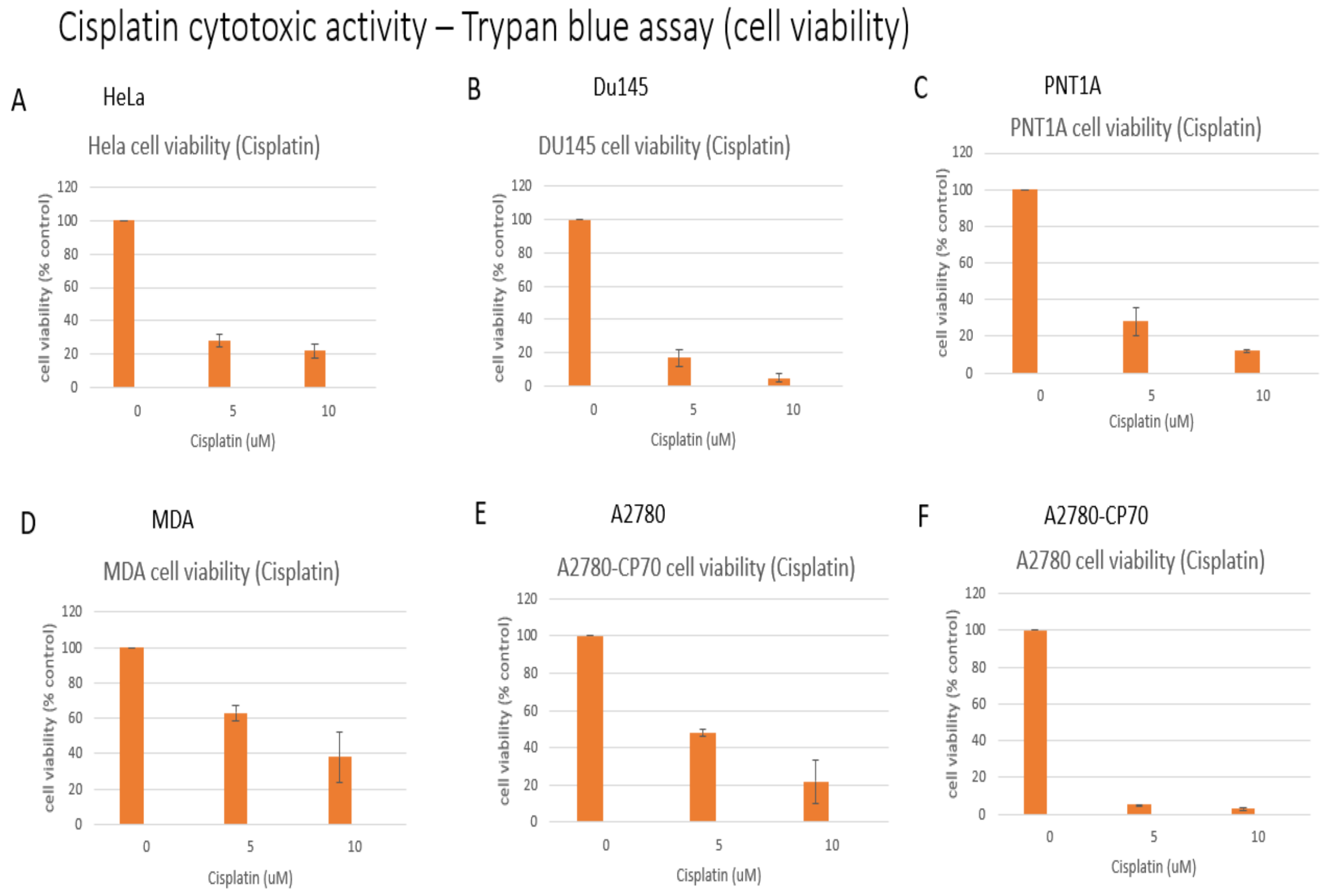

Figure S2D: Trypan blue assay results for cisplatin treatment on various cell lines. Error was calculated using Standard error of the mean (SEM).

S3: Epifluorescense microscopy (Nuclear morphology)

S3A:

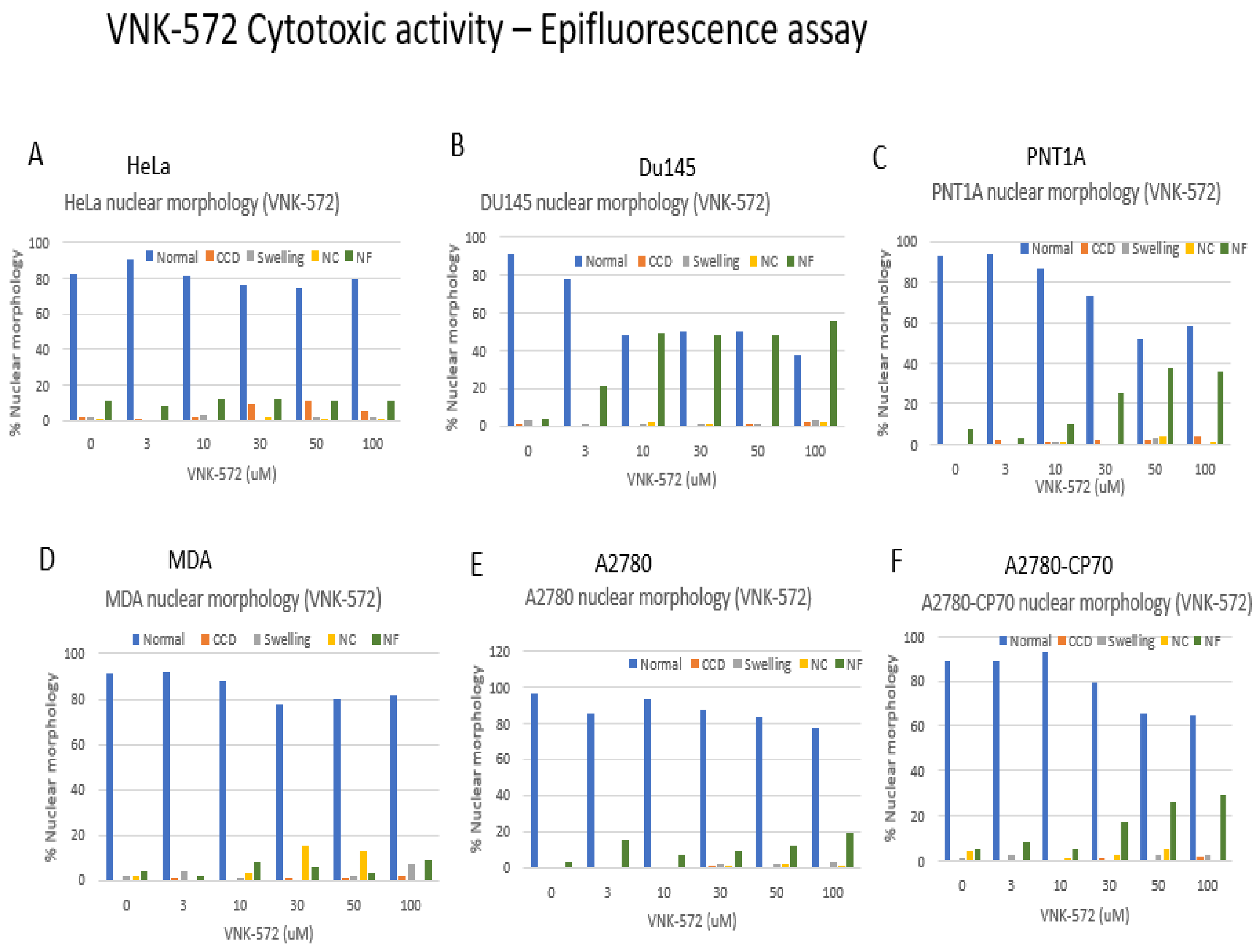

Figure S3A: Epifluorescense microscopic image results for VNK-572 treatment on various cell lines.

S3B

VNK-754 Cytotoxic activity – Epifluorescence assay

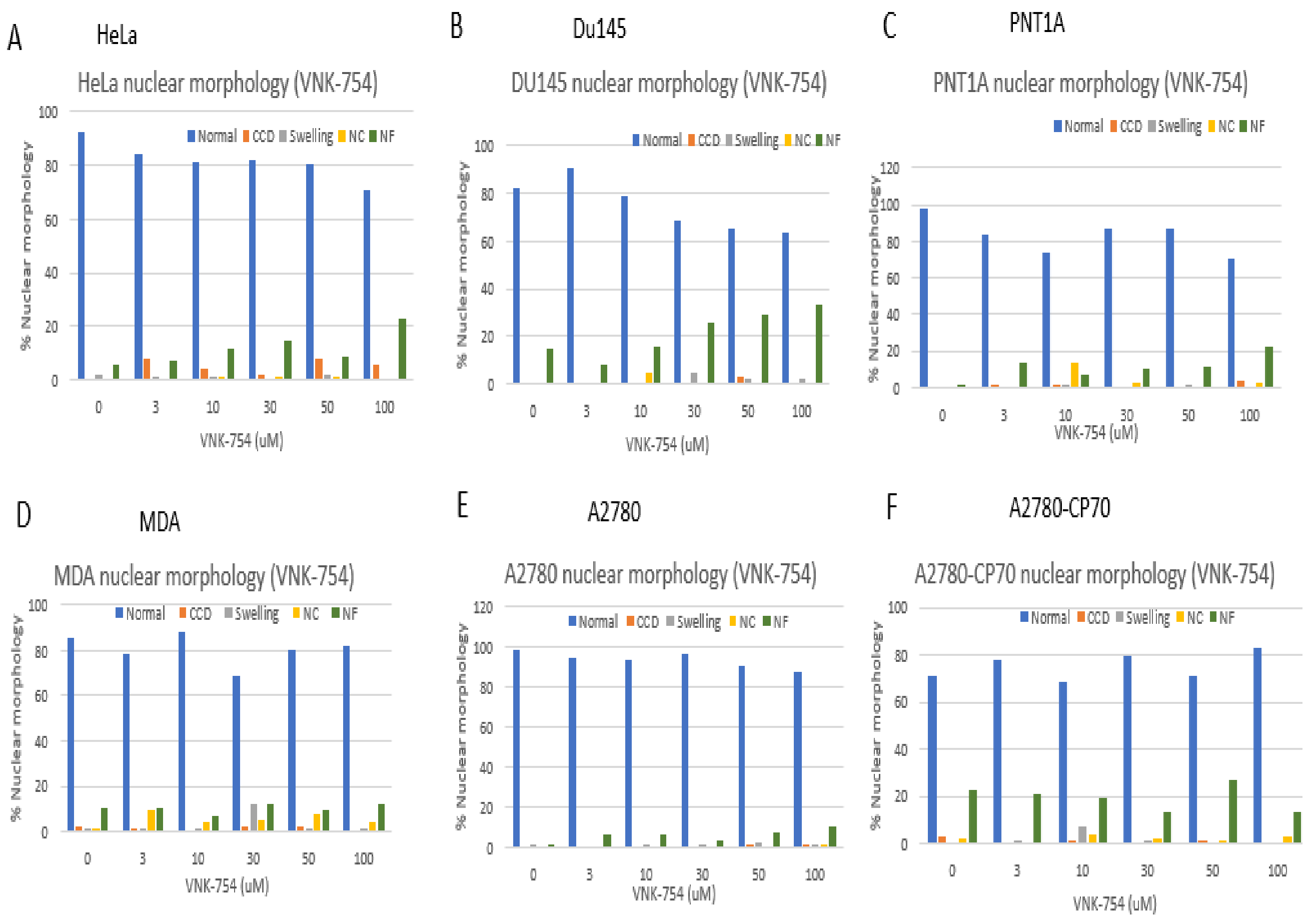

Figure S3B: Epifluorescense microscopic image results for VNK-754 treatment on various cell lines.

S3C

POW-12A Cytotoxic activity – Epifluorescence assay

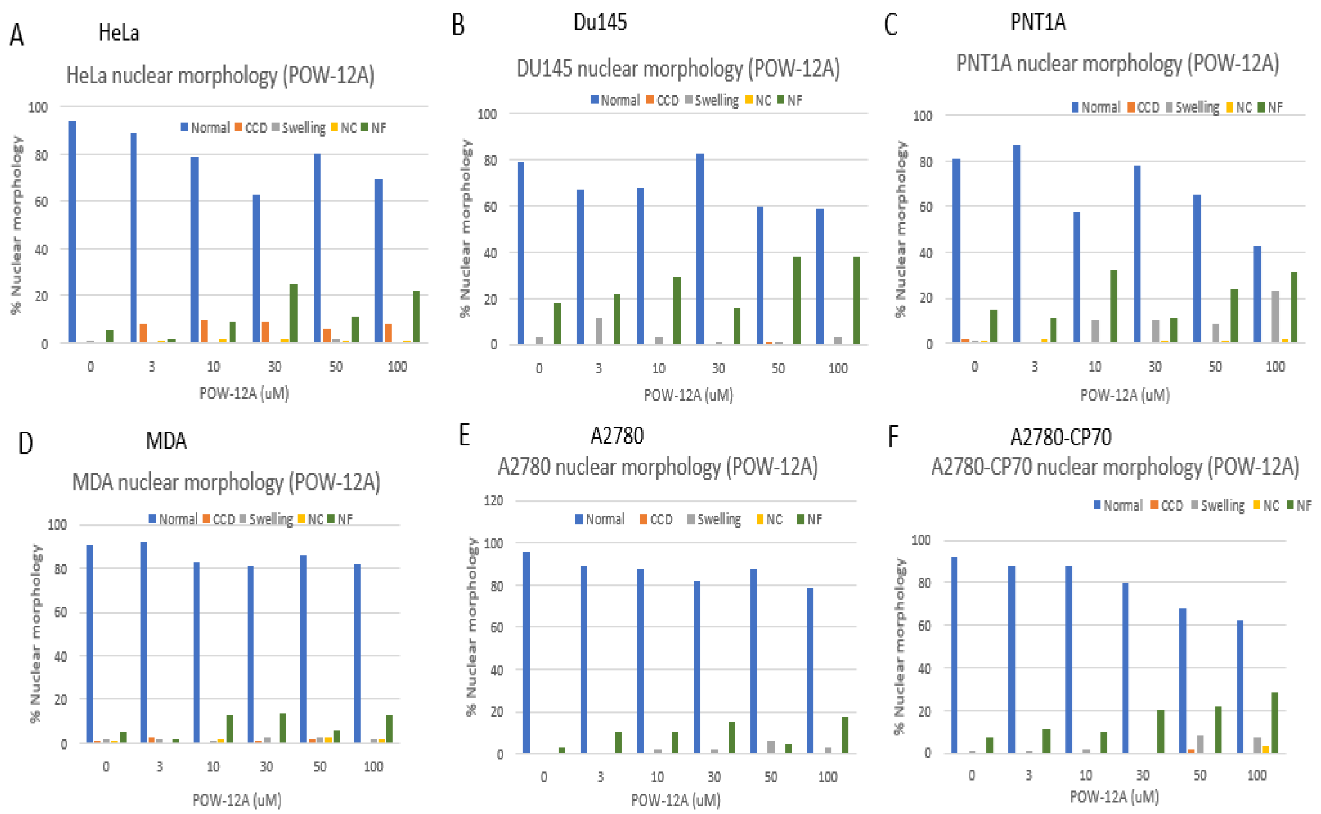

Figure S3C: Epifluorescence microscopic image results for VNK-754 treatment on various cell lines.

S3D

Cisplatin cytotoxic activity – Epifluorescence assay

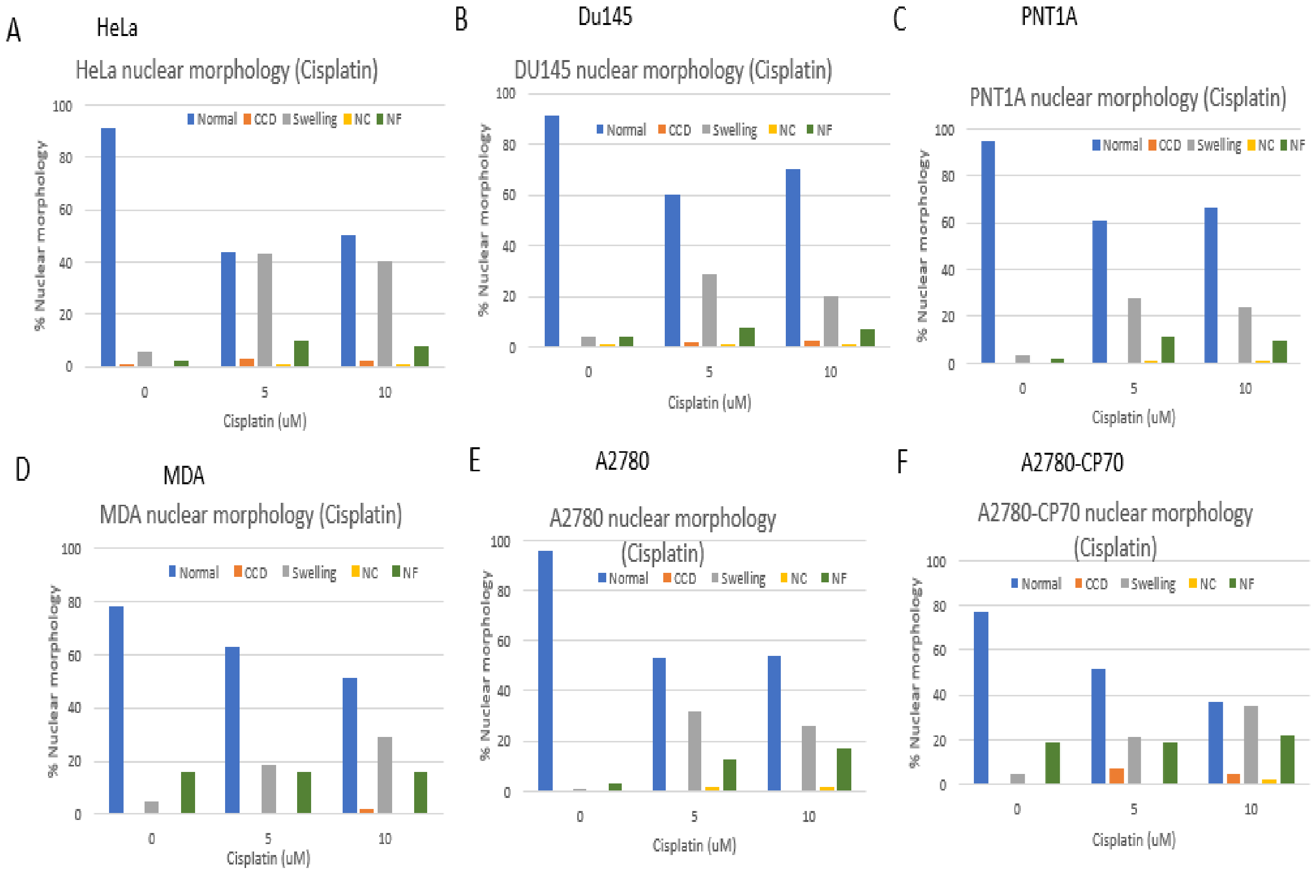

Figure S3D: Epifluorescence microscopic image results for Cisplatin treatment on various cell lines.

S4: Microscopic images (Cell and nucleus)

S4A

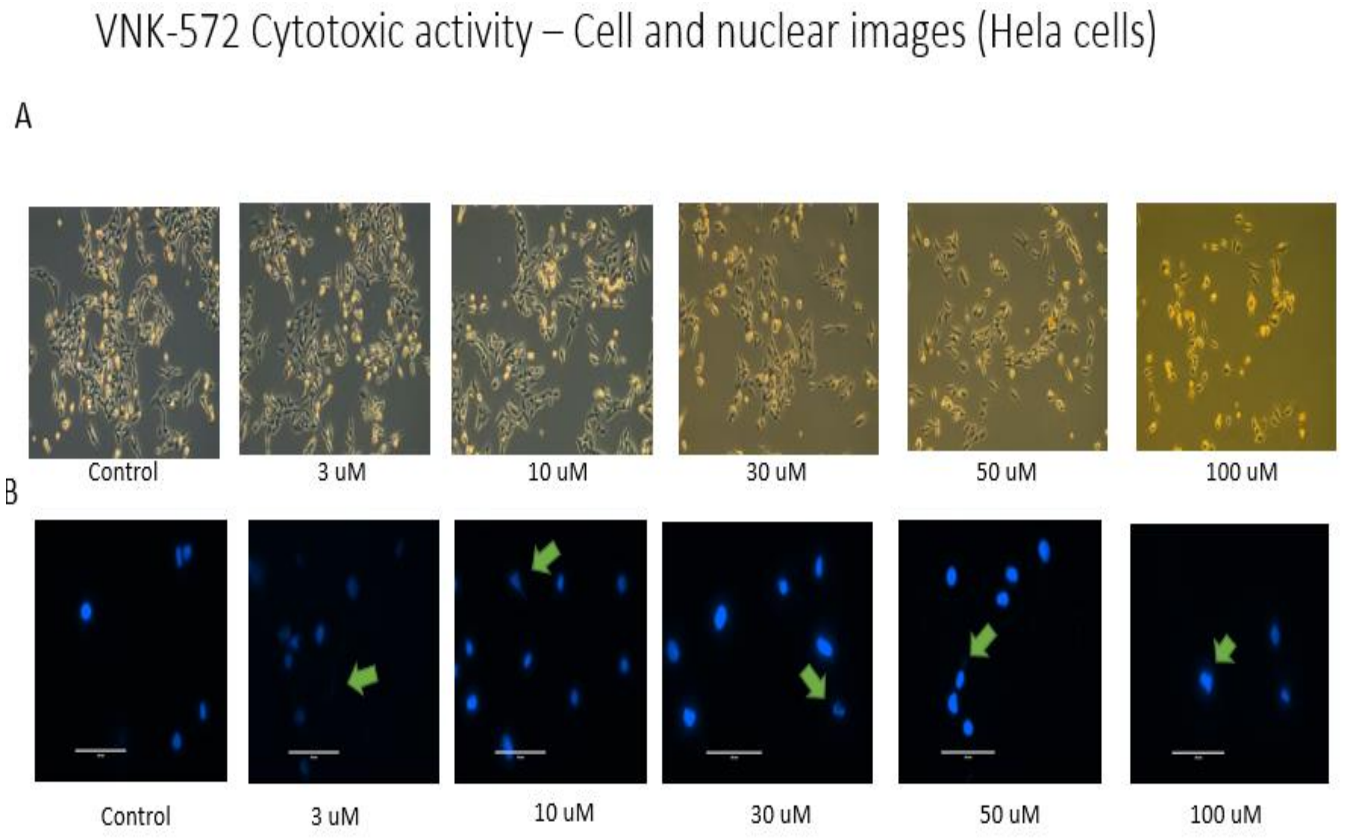

Figure S4A: (A) Light microscope images of Hela cell densities after treatment with different concentrations of VNK-572 at 37°C for 72h. (B) Epifluorescence microscope images of Hela nuclear morphology after VNK-572 treatment. Green arrows indicate nuclear fragmentation.

S4B

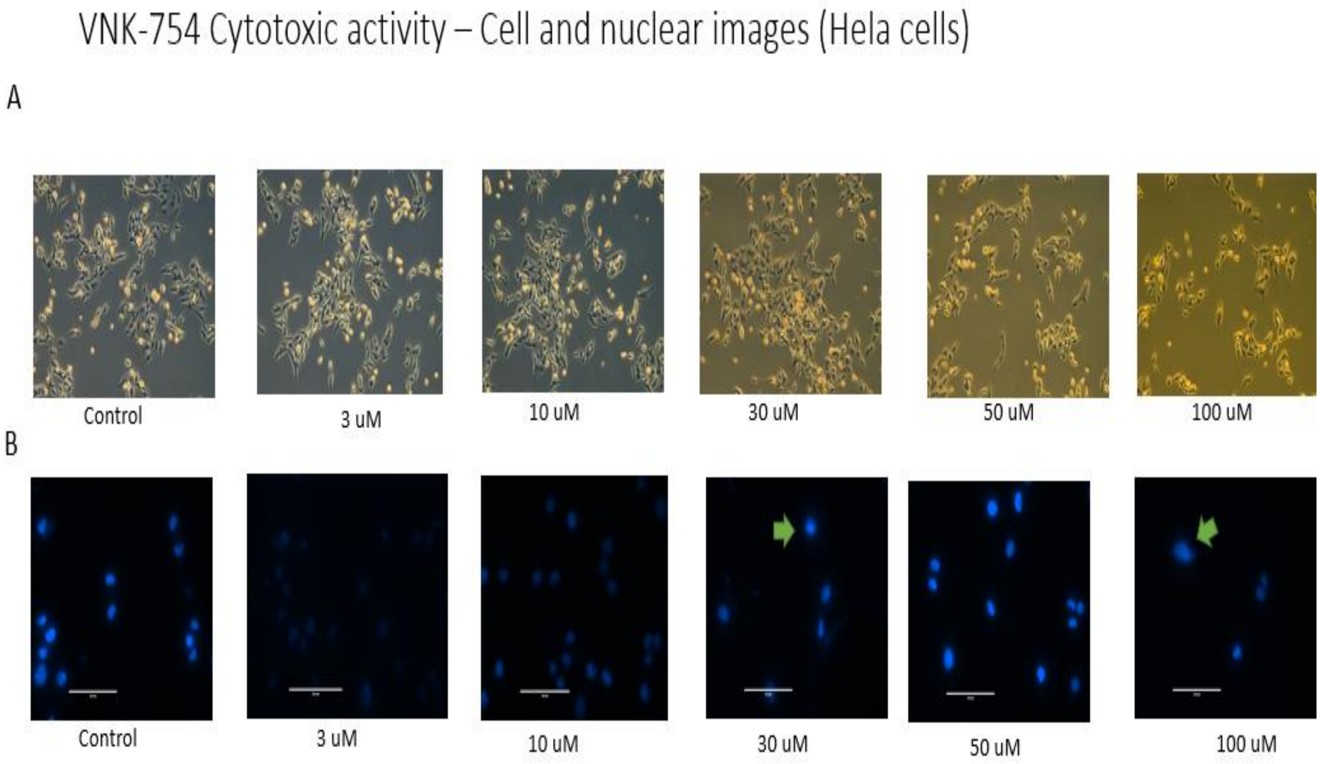

Figure S4B: (A) Light microscope images of Hela cell densities after treatment with different concentrations of VNK-754 at 37℃ for 72h. (B) Epifluorescence microscope images of Hela nuclear morphology after VNK-754 treatment. Green arrows indicate nuclear fragmentation.

S4C

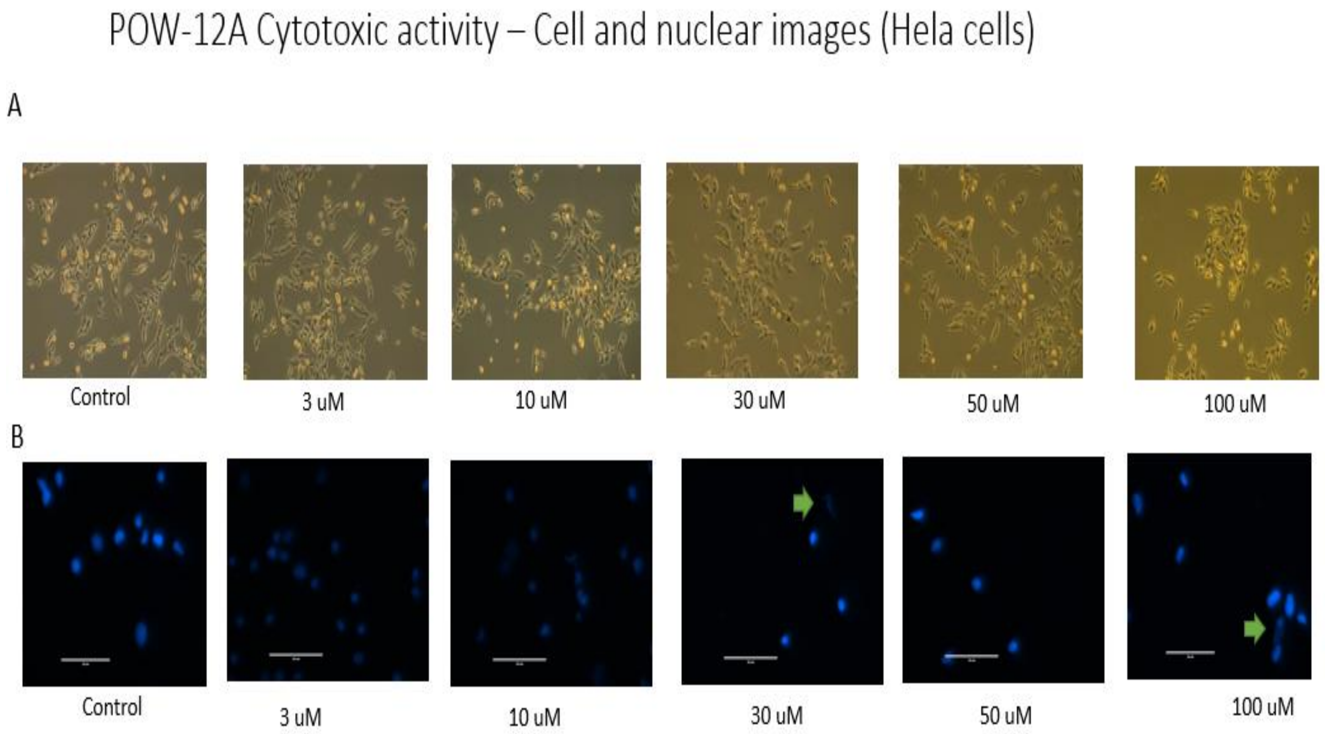

Figure S4C: (A) Light microscope images of Hela cell densities after treatment with different concentrations of POW-12A at 37℃ for 72h. (B) Epifluorescence microscope images of Hela nuclear morphology after POW-12A treatment. Green arrows indicate nuclear fragmentation.

S4D

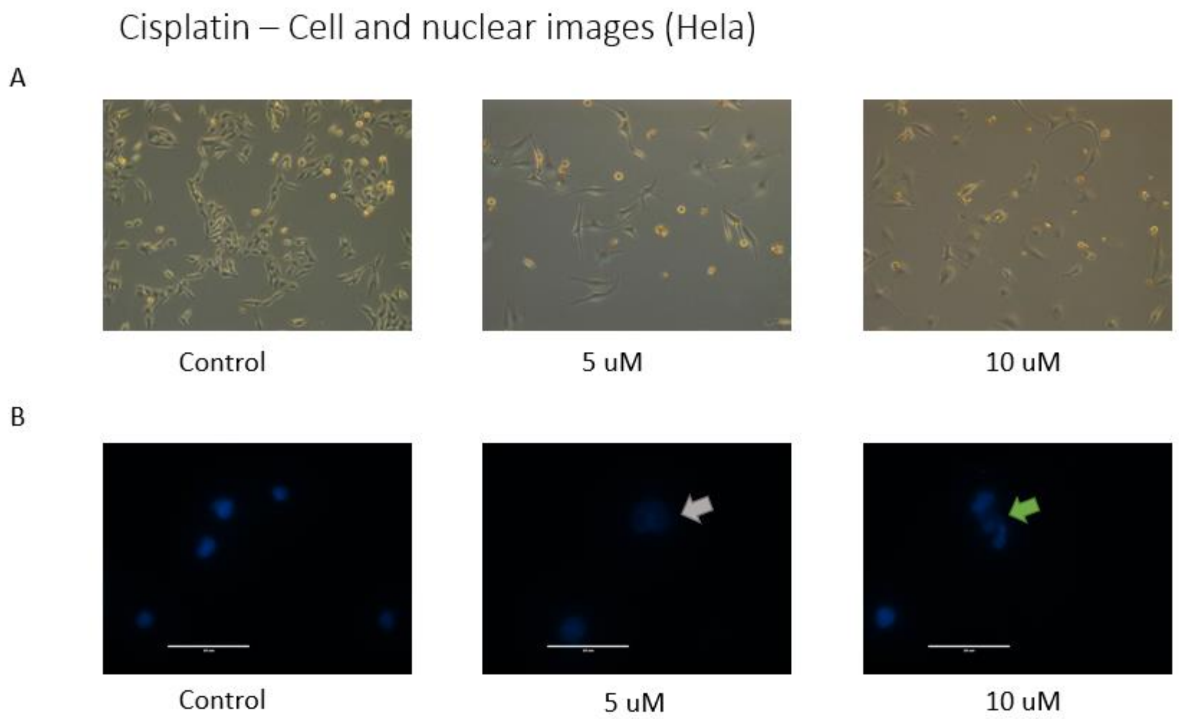

Figure S4D: (A) Light microscope images of Hela cell densities after treatment with different concentrations Cisplatin at 37℃ for 72h. (B) Epifluorescence microscope images of Hela nuclear morphology after Cisplatin treatment. Green arrow indicate nuclear fragmentation, while grey arrow indicates nuclear swelling.

S5: Log P

S5A: VNK-572

S5A1

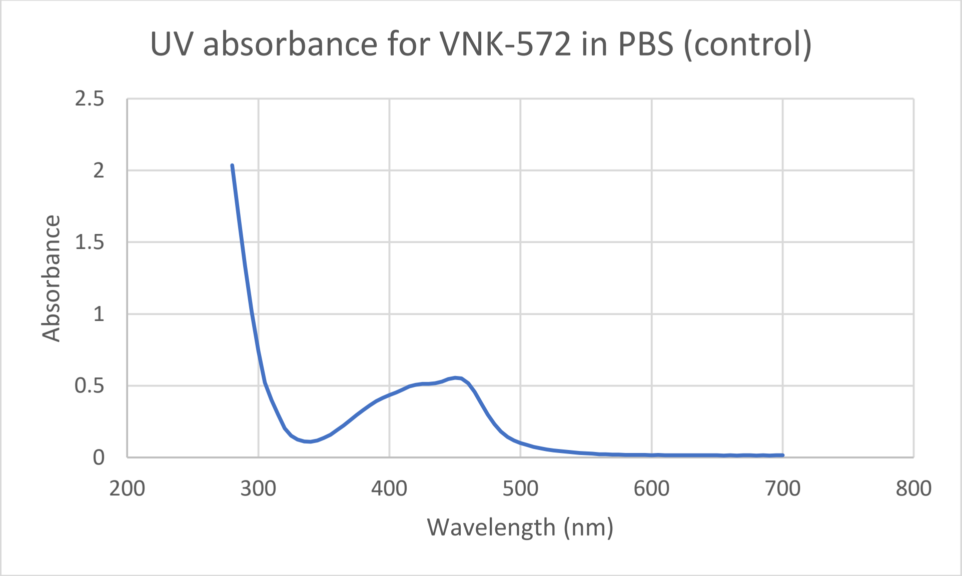

Figure S5A1: Graph of UV absorbance of VNK-572 in PBS.

S5A2

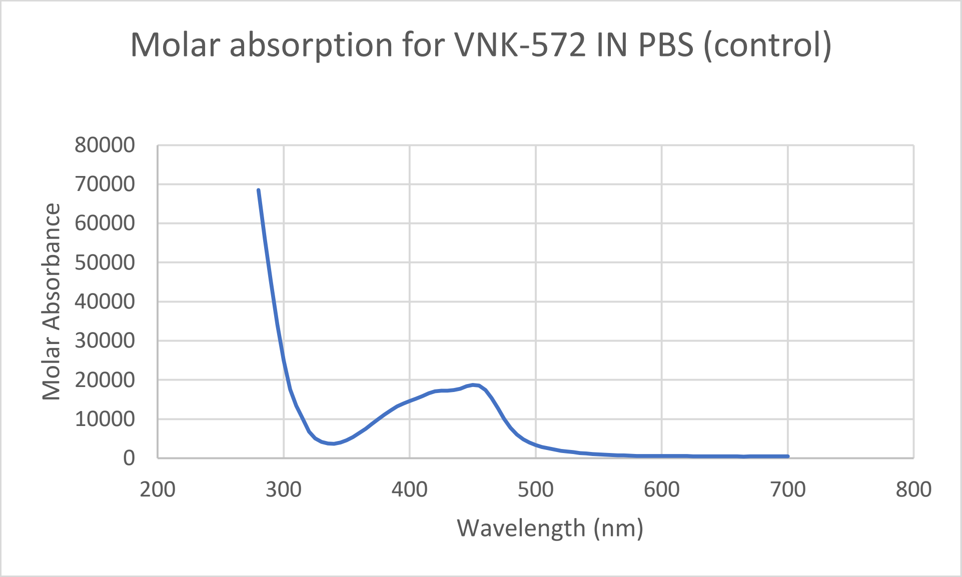

Figure S5A2: Graph of molar absorbance of VNK-572 in PBS.

S5A3

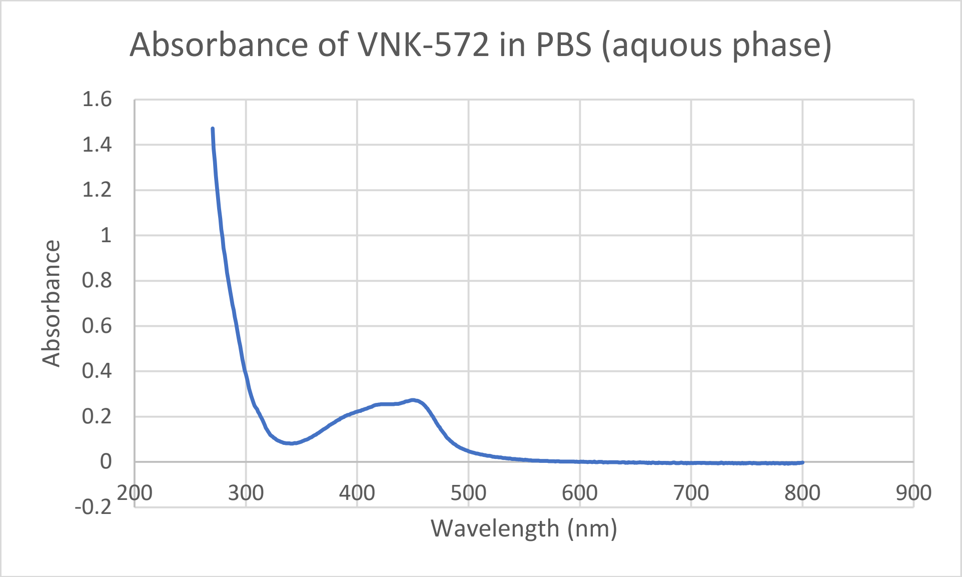

Figure S5A3: Graph of UV absorbance of VNK-572 in aqueous phase (PBS) after shake-flask procedure.

S5B: VNK-754

S5B1

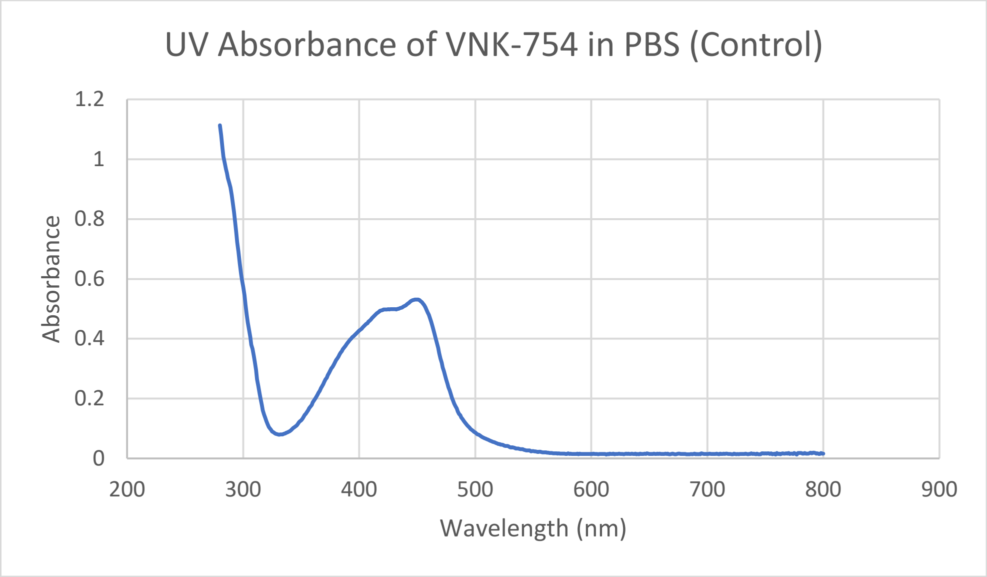

Figure S5B1: UV absorbance spectrum of VNK-754 in PBS.

S5B2

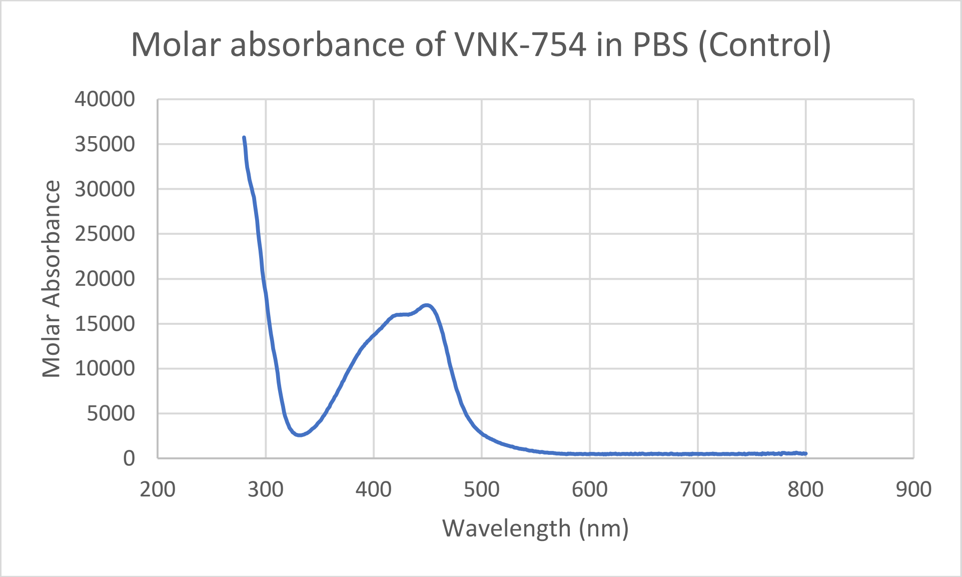

Figure S5B2: Graph of molar absorbance of VNK-754 in PBS.

S5B3

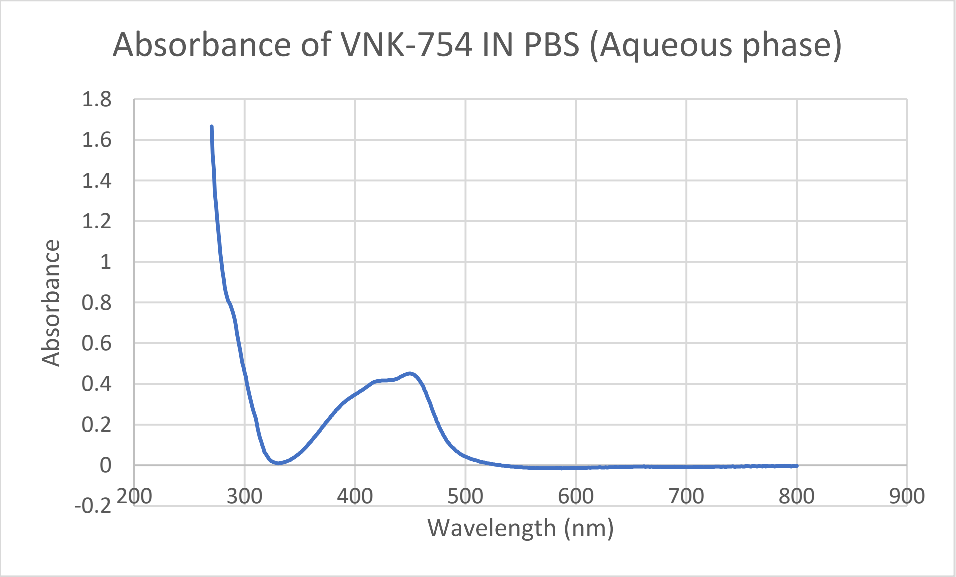

Figure S5B3: Graph of UV absorbance of VNK-754 in aqueous phase (PBS) after shake-flask procedure.

S5C: POW-12A

S5C1:

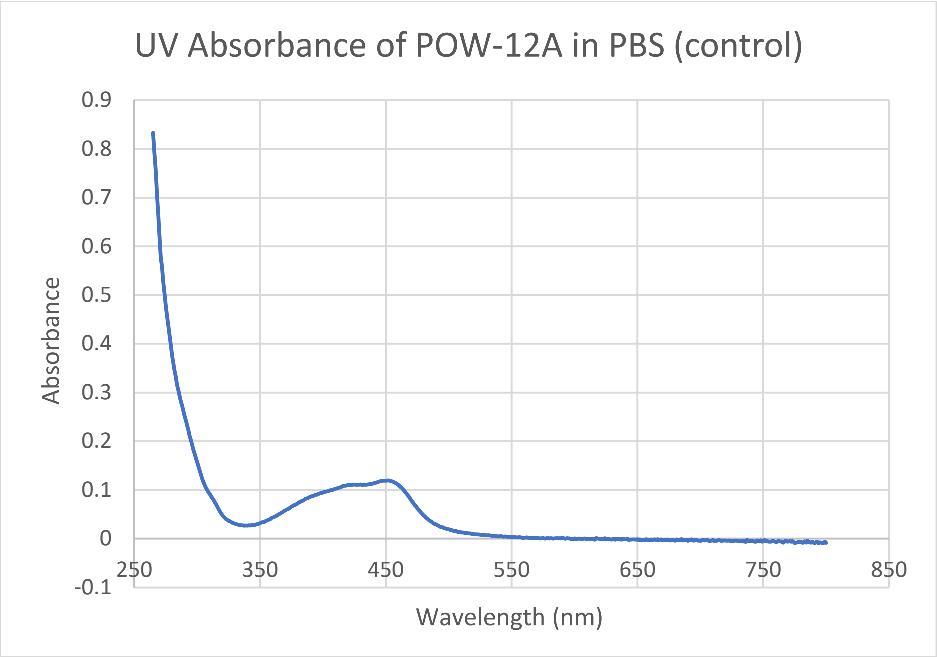

Figure S5C1: UV absorbance spectrum of POW-12A in PBS.

S5C2:

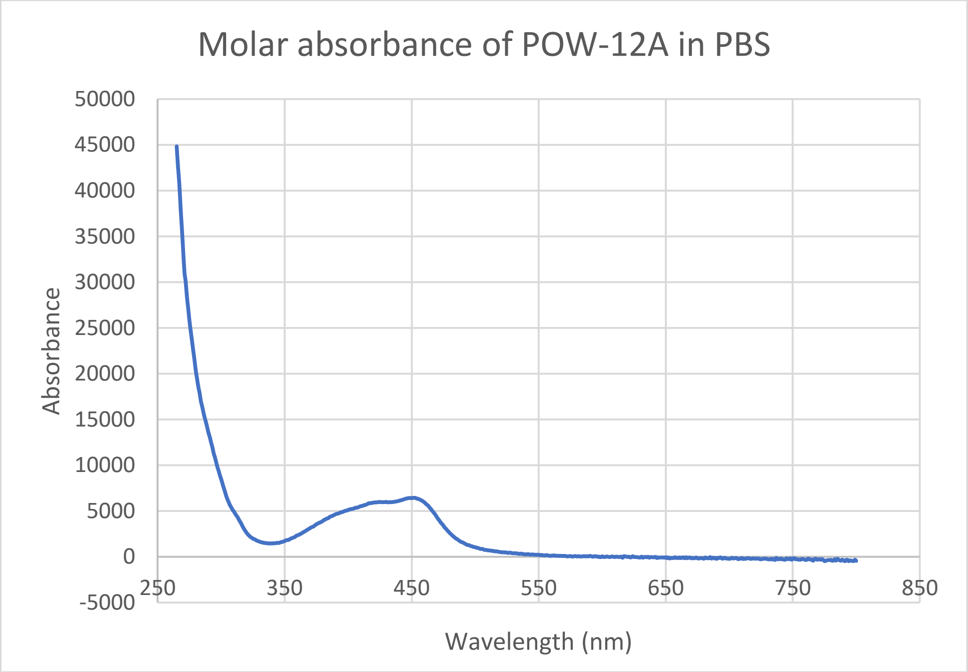

Figure S5C2: Graph of molar absorbance of POW-12A in PBS.

S5C3:

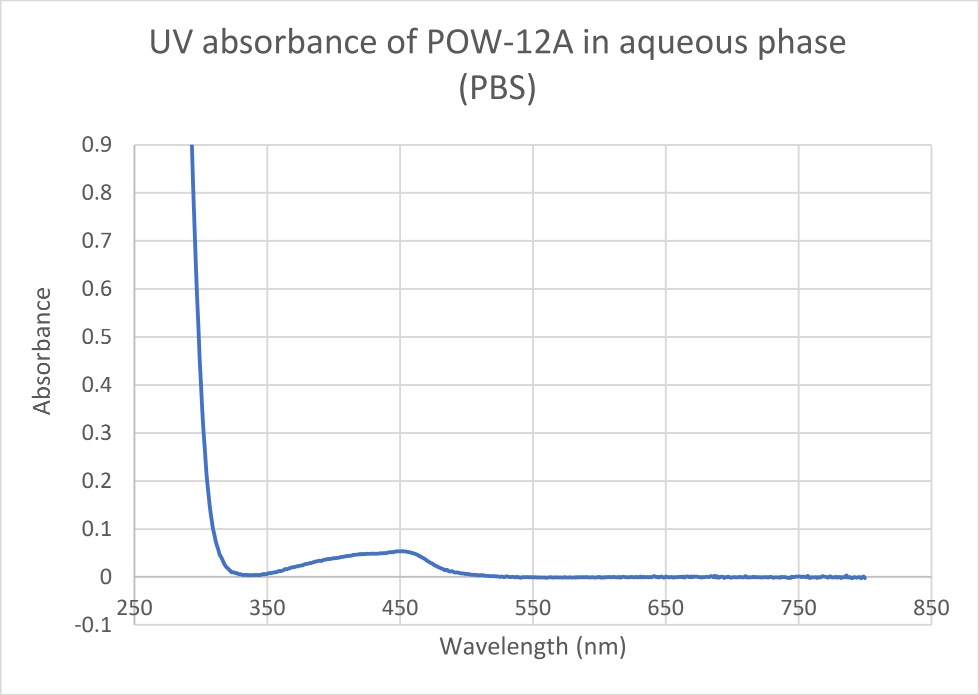

Figure S5B3: Graph of UV absorbance of POW-12A in aqueous phase (PBS) after shake-flask procedure.

S6: Annexin-V assay (Cisplatin)

S6A

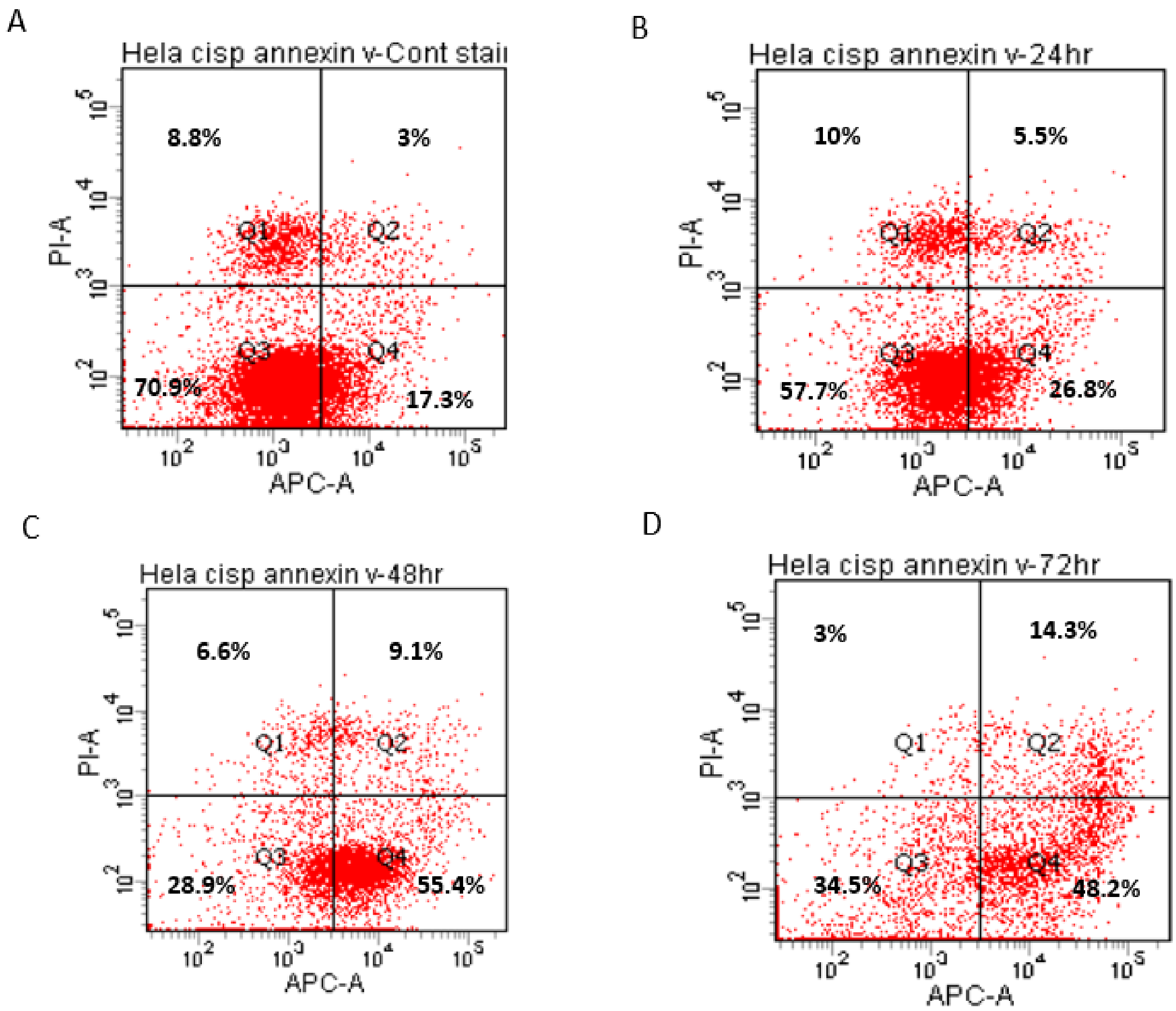

Figure S6A: Annexin-V scatter plots of Hela cells after treatment with cisplatin for **A**. Control **B**. 24h **C**. 48h and **D**. 72h, respectively. **Q1** and **Q2** represent cells undergoing necrosis and late apoptosis, **Q3** are normal cells, while **Q4** represent cells undergoing early apoptosis.

S6B

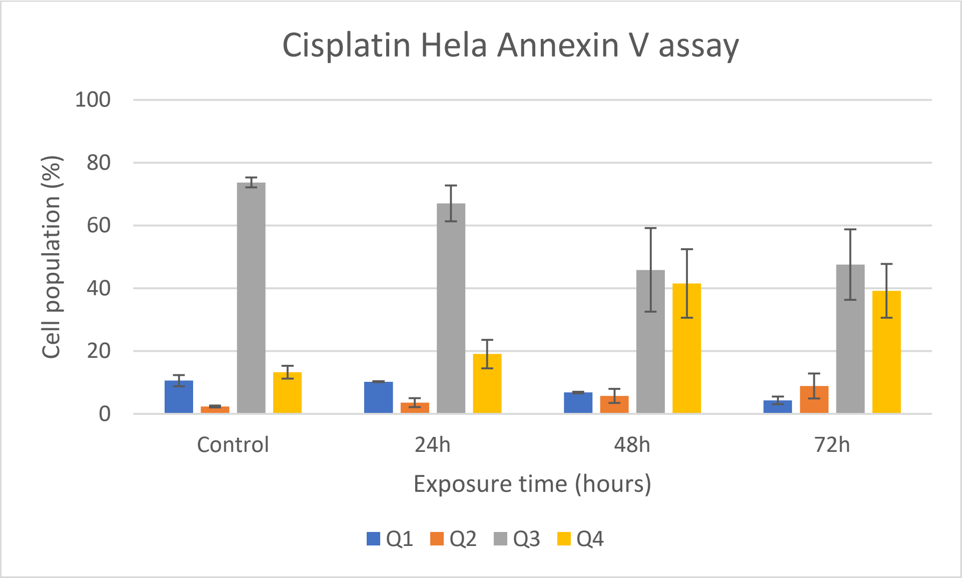

Figure S6B: Histogram distribution of percentages of Hela cells in Q1, Q2, Q3 and Q4 after treatment with cisplatin for 24h, 48h and 72h, respectively. Q1 and Q2 represent cells undergoing necrosis, Q3 are normal cells, while Q4 represent cells undergoing apoptosis.

S7: Confocal microscopy in DU145

S7A: Complexes alone

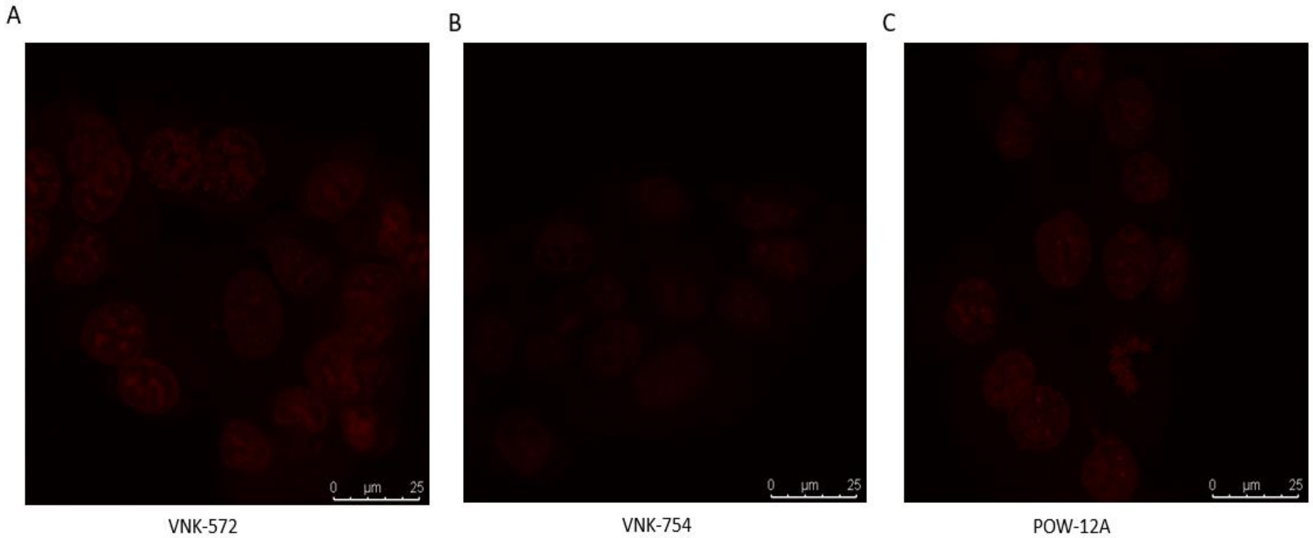

Figure S7A: A. Confocal images of **VNK-572, VNK-754 and POW-12A** nuclear localization in DU145 cells after respective treatments with the complexes alone. The complexes were viewed under a CLSM Leica SP microscope using the wavelength settings (excitation, 405 nm; emission, 570 – 630 nm).

S7B:

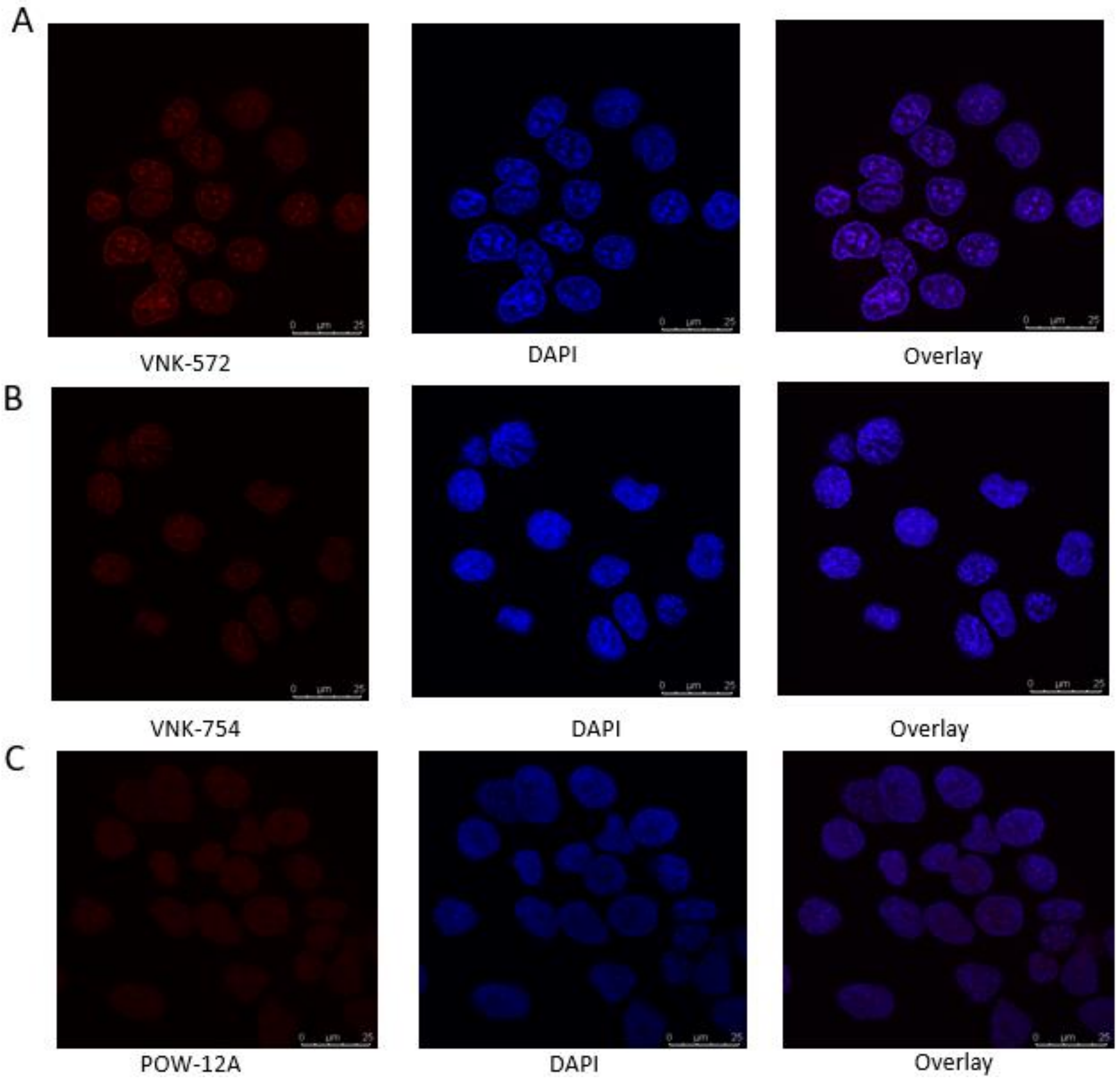

Figure S7B: Fluorescence Confocal images of ruthenium complexes nuclear localization in DU145 cells after incubating with (A) **VNK-572** (50 µM), (B) **VNK-754** (50 µM) and (C) **POW-12A** (50 µM) . Images show fluorescence of the compounds (red), DAPI (blue), and an overlay of each respective complex with DAPI after counter-staining. The complexes were viewed under the CLSM Leica SP microscope using the wavelength settings (excitation, 405 nm; emission, 570 – 630 nm) while DAPI was observed using the following settings (excitation, 405 nm; emission, 430 – 480 nm).

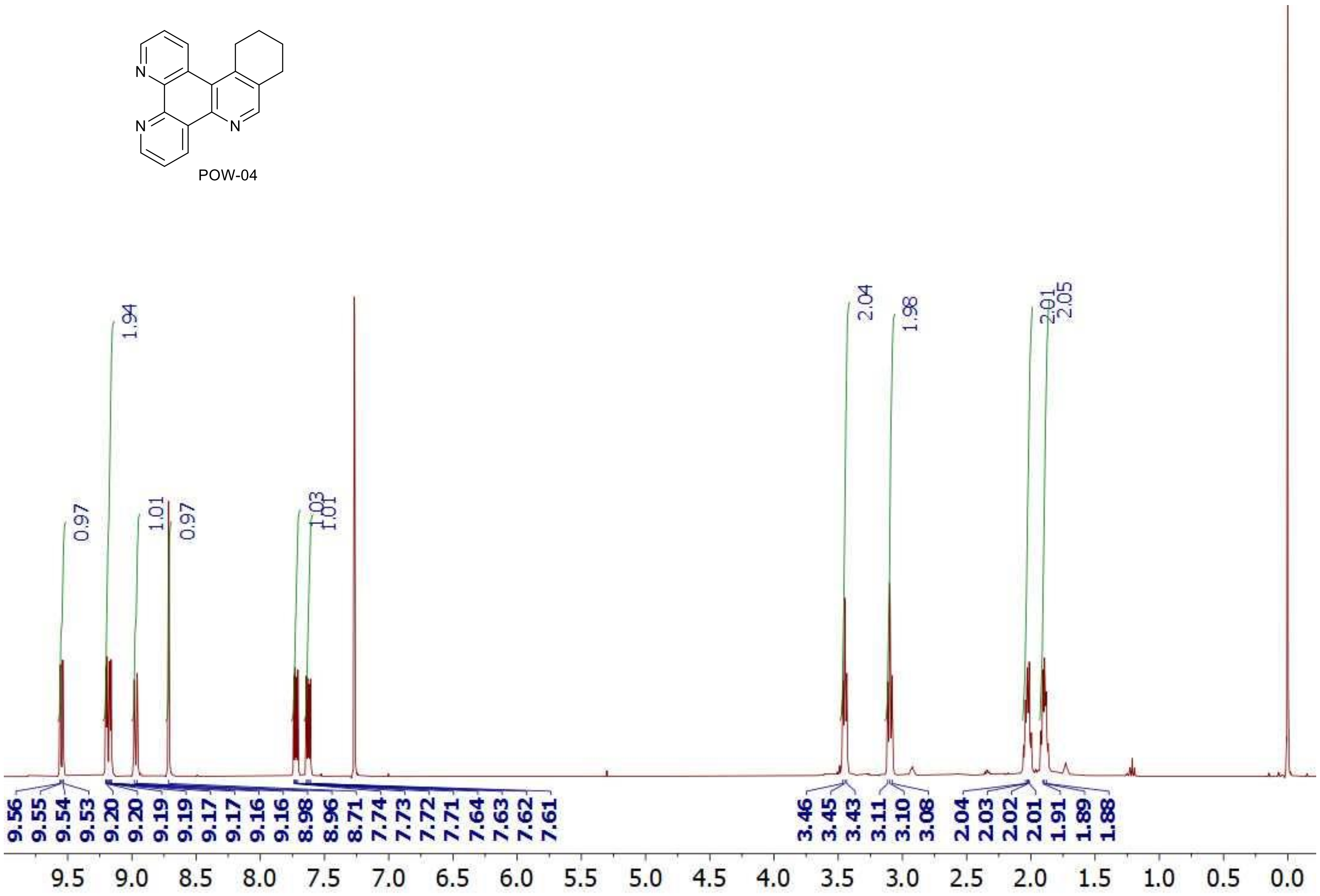

Figure SX. ^{1}H NMR spectrum of POW-04 in chloroform-D.

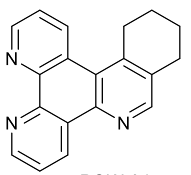

POW-04

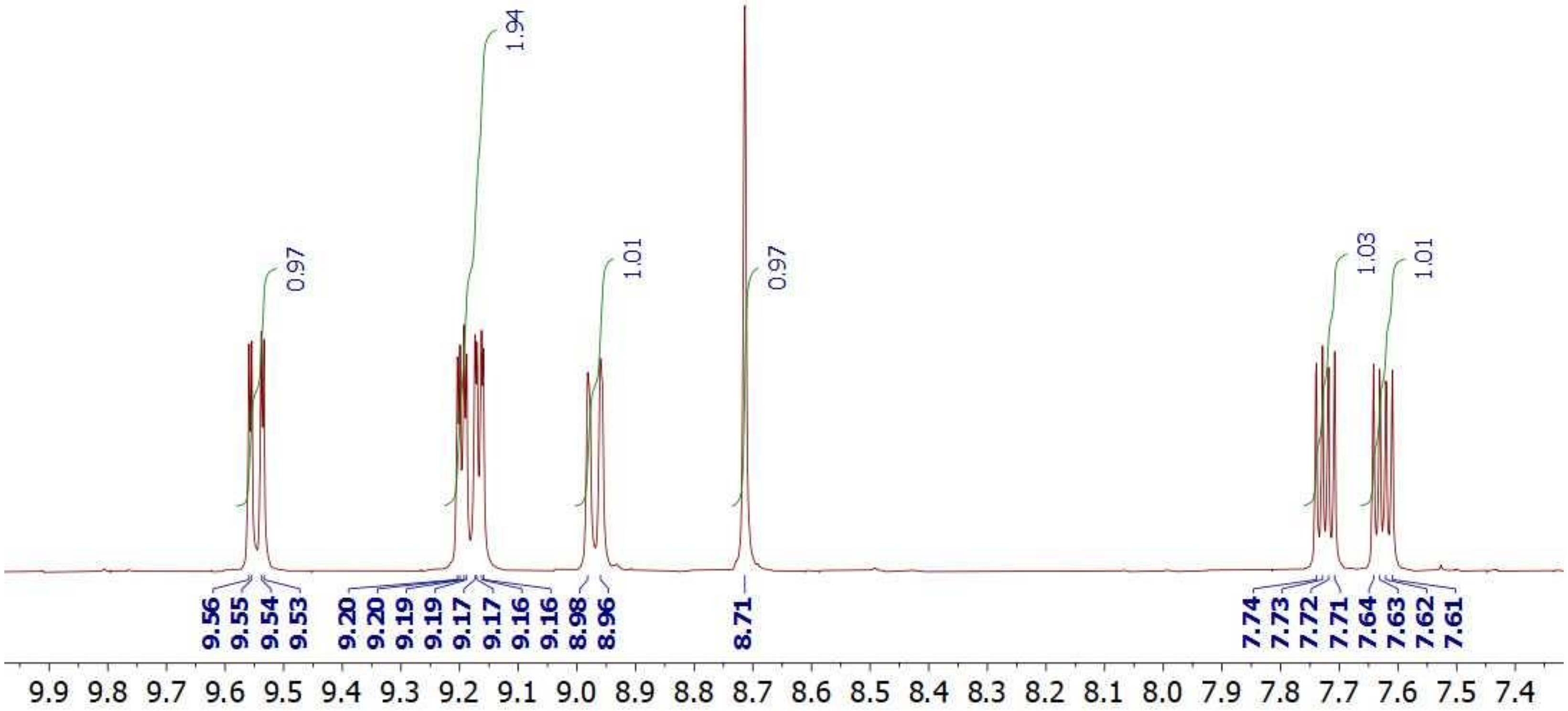

Figure SX. Aromatic region of the ^{1}H NMR spectrum of POW-04 in chloroform-D.

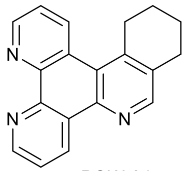

POW-04

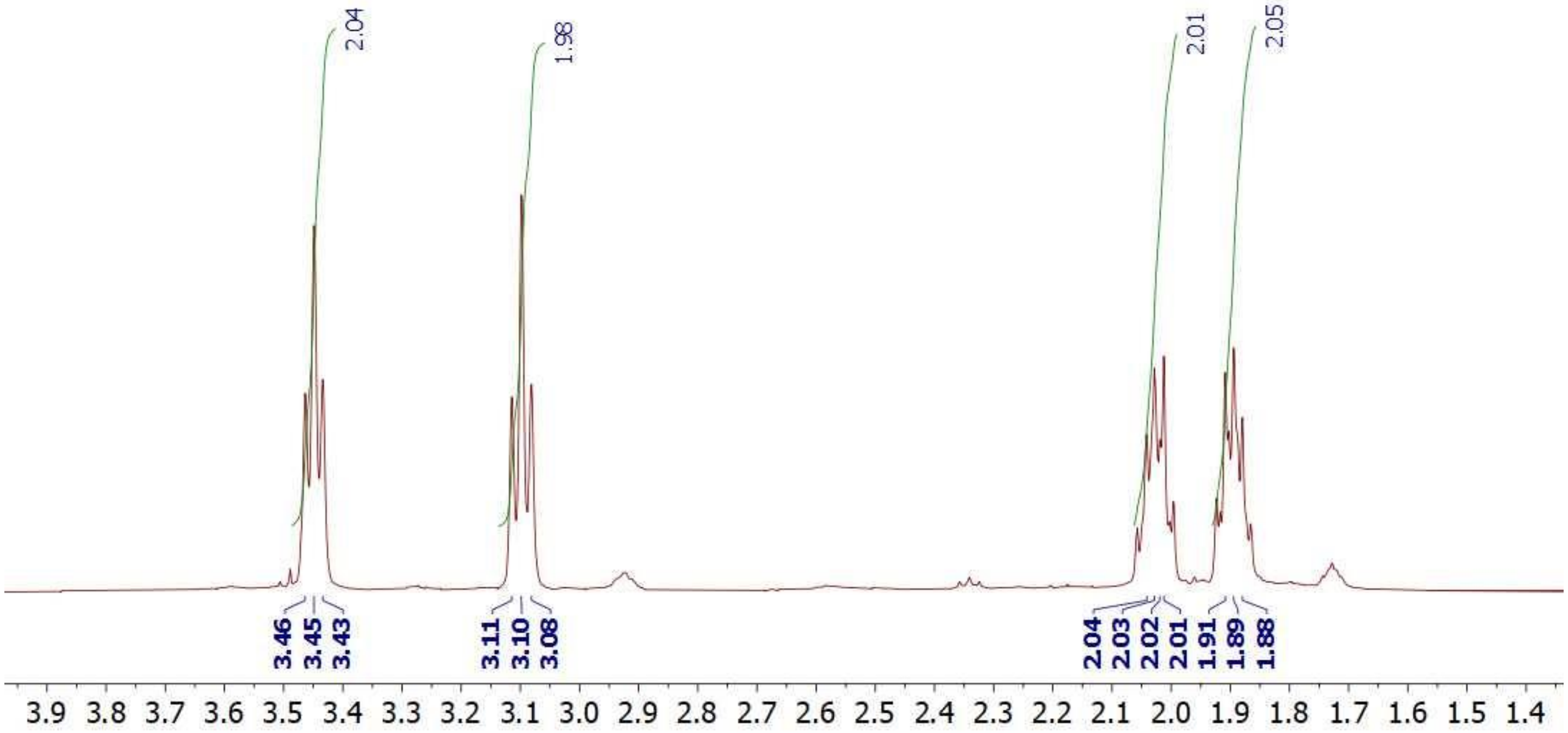

Figure SX. Aliphatic region of the ^{1}H NMR spectrum of POW-04 in chloroform-D.

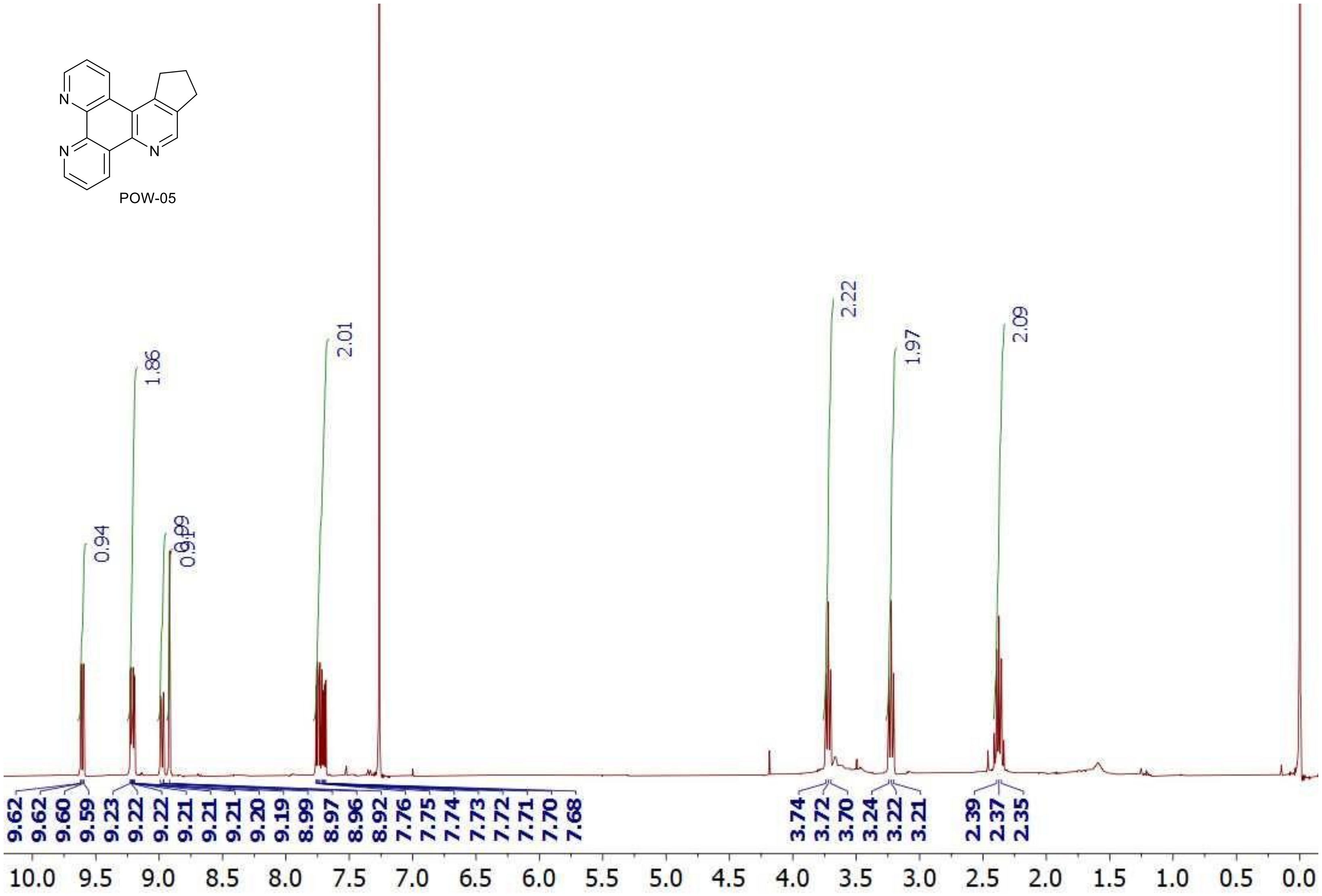

Figure SX. ^{1}H NMR spectrum of POW-05 in chloroform-D.

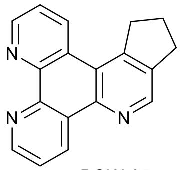

POW-05

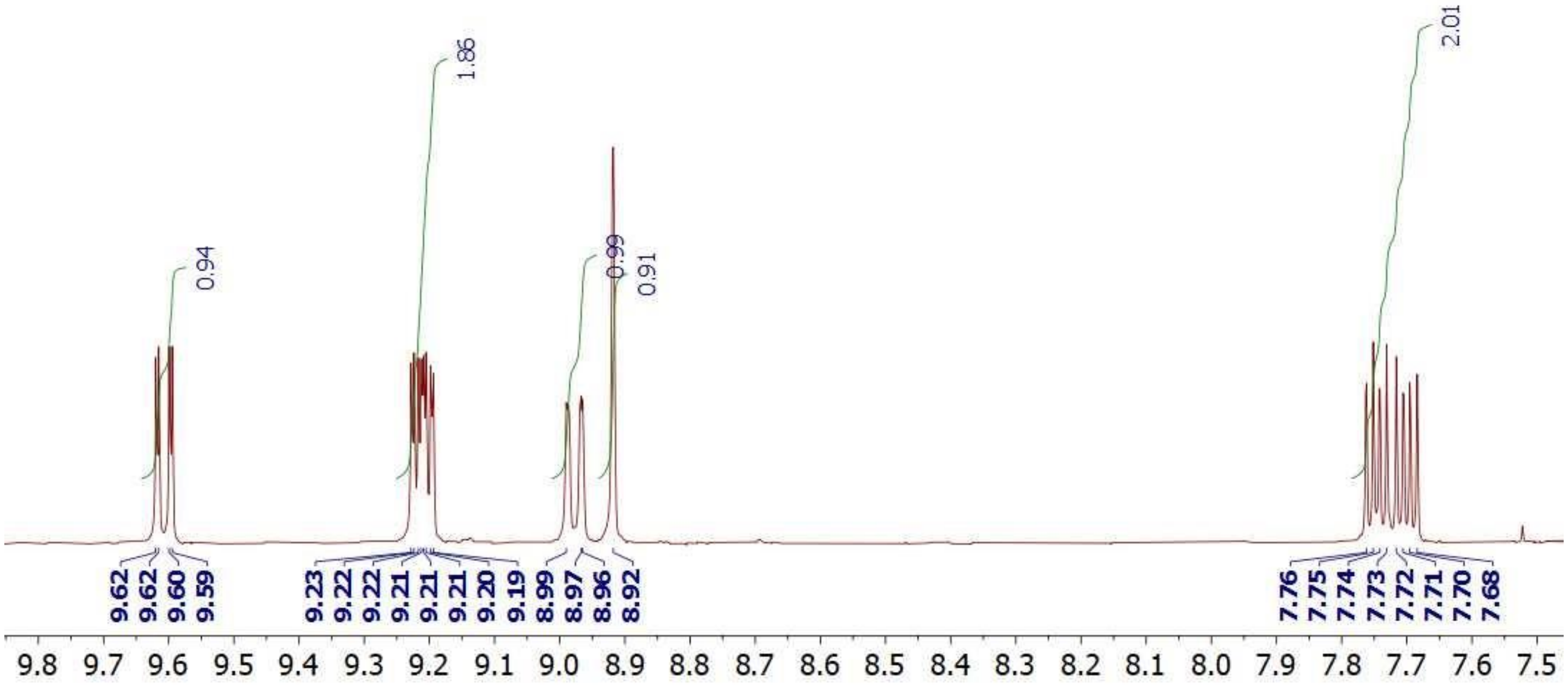

Figure SX. Aromatic region of the ^{1}H NMR spectrum of POW-05 in chloroform-D.

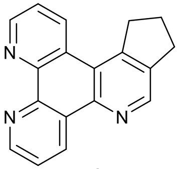

POW-05

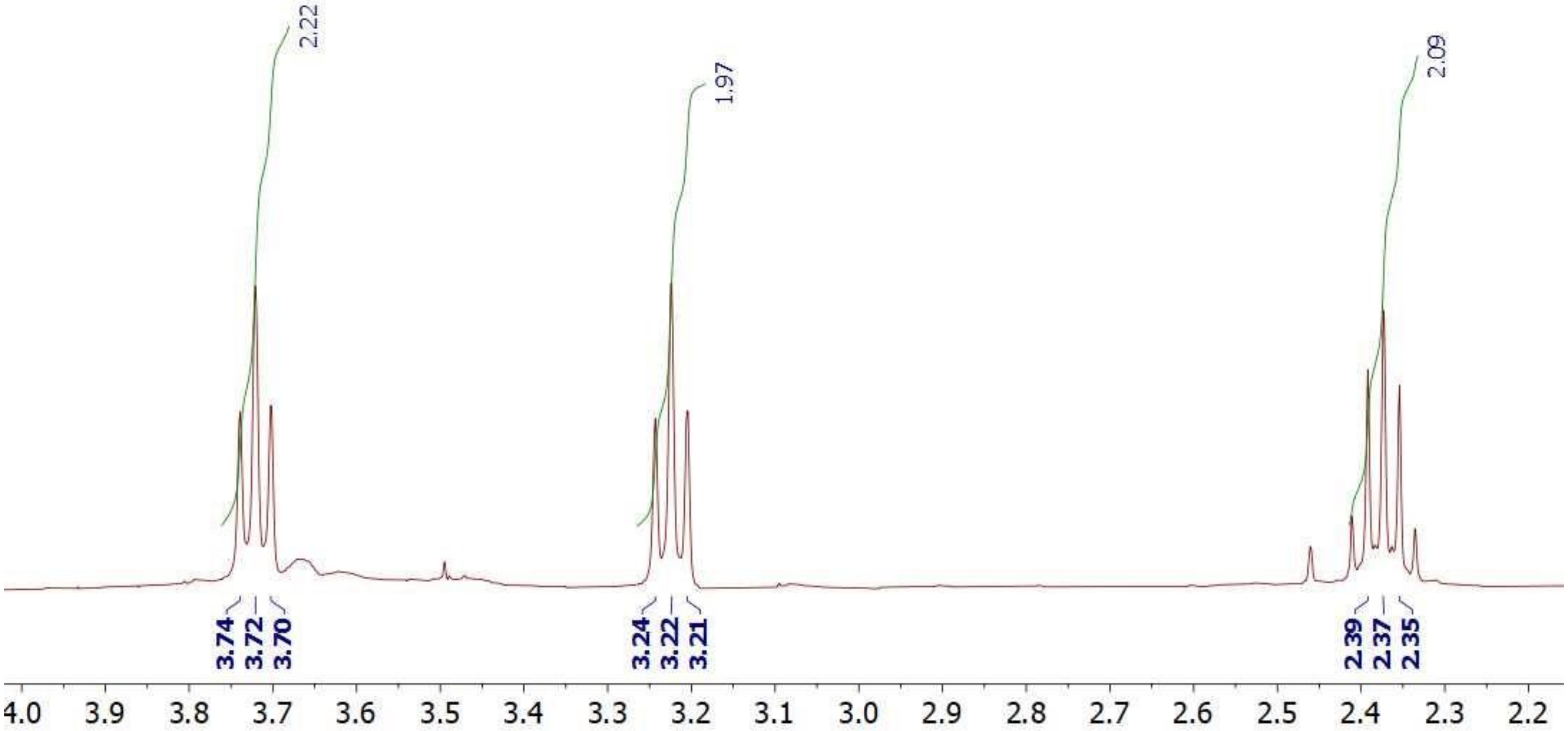

Figure SX. Aliphatic region of the ^{1}H NMR spectrum of POW-05 in chloroform-D.

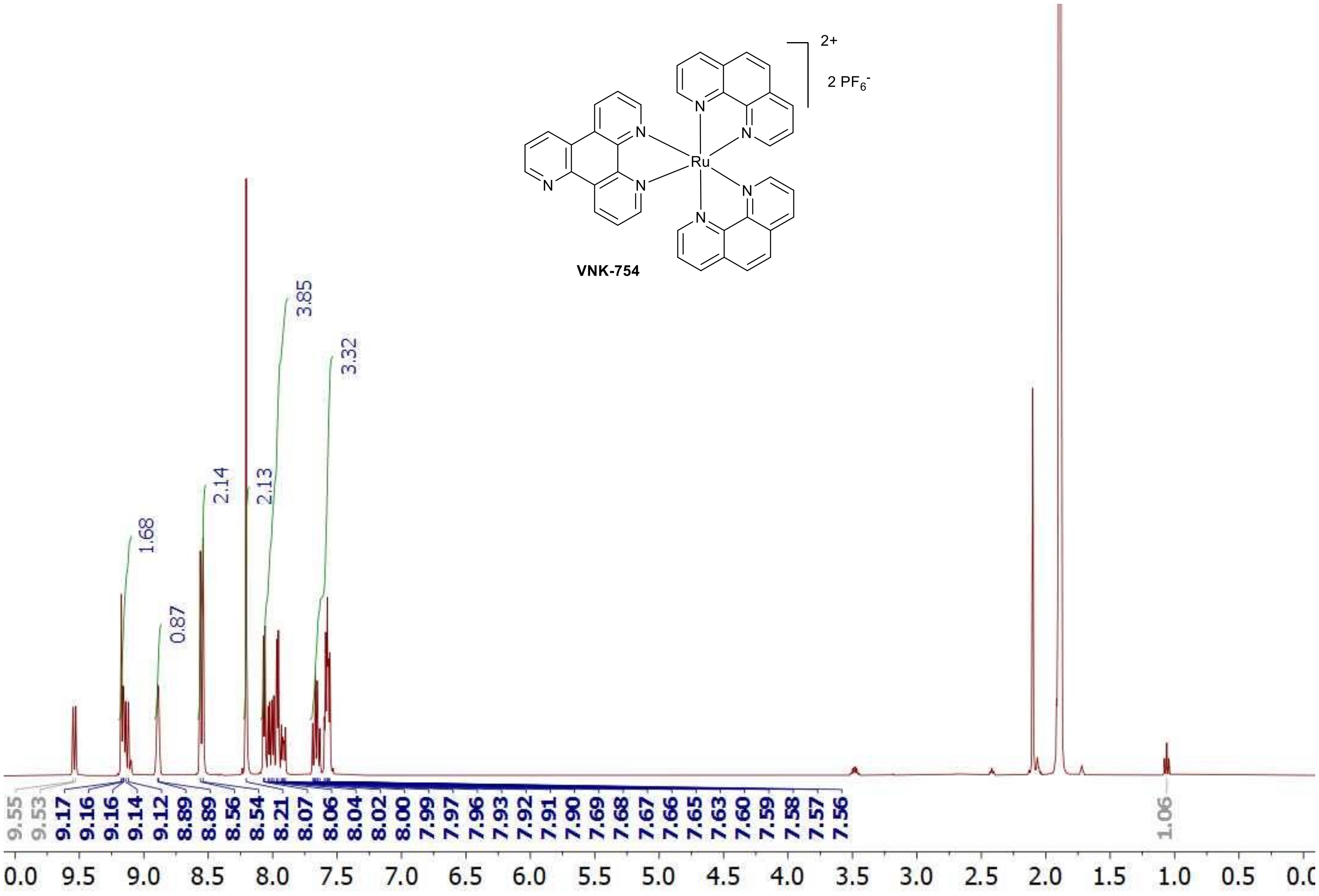

Figure SX. ^{1}H NMR spectrum of VNK-754 in acetonitrile-D3.

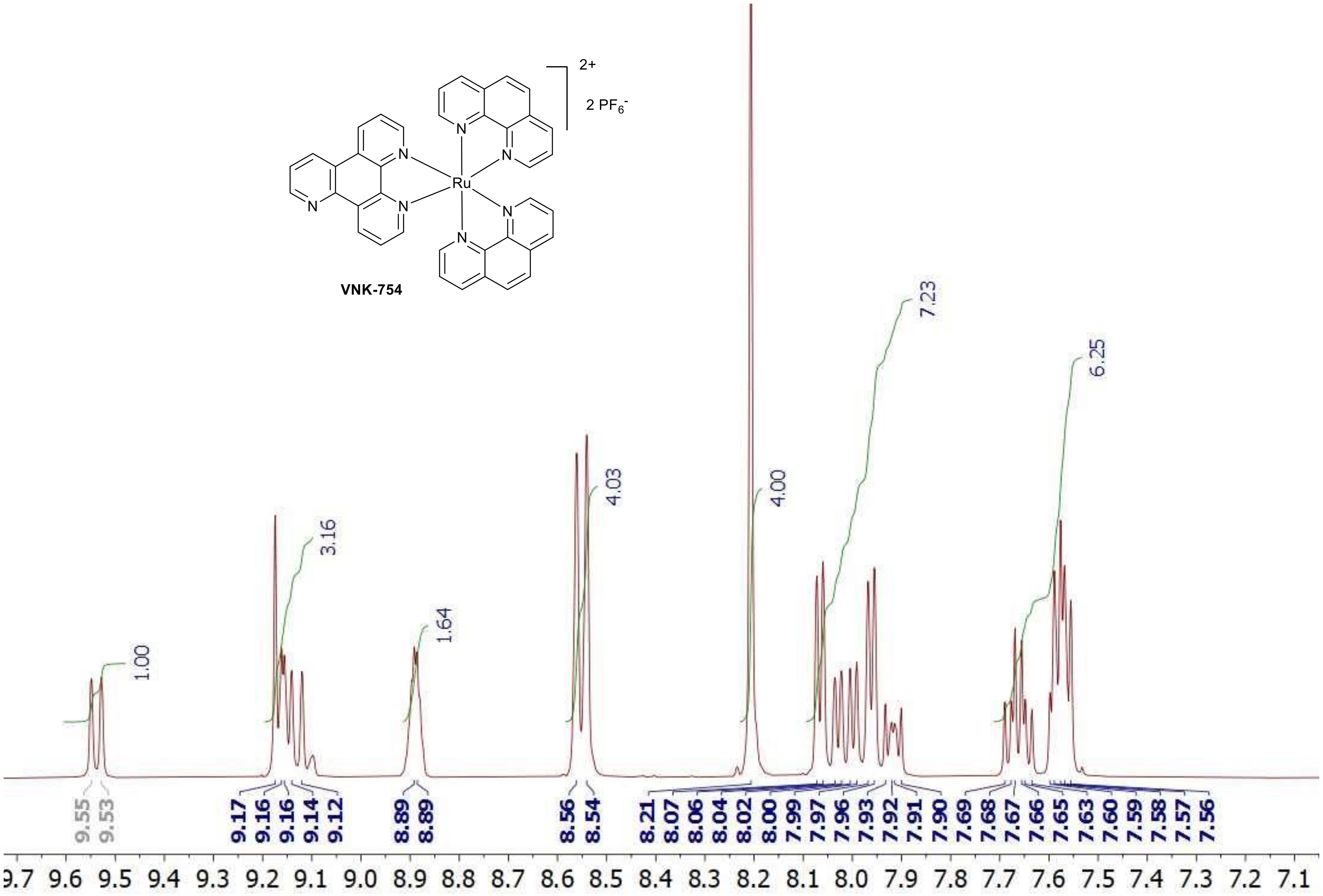

Figure SX. Aromatic region of the ^{1}H NMR spectrum of VNK-754 in acetonitrile-D3.

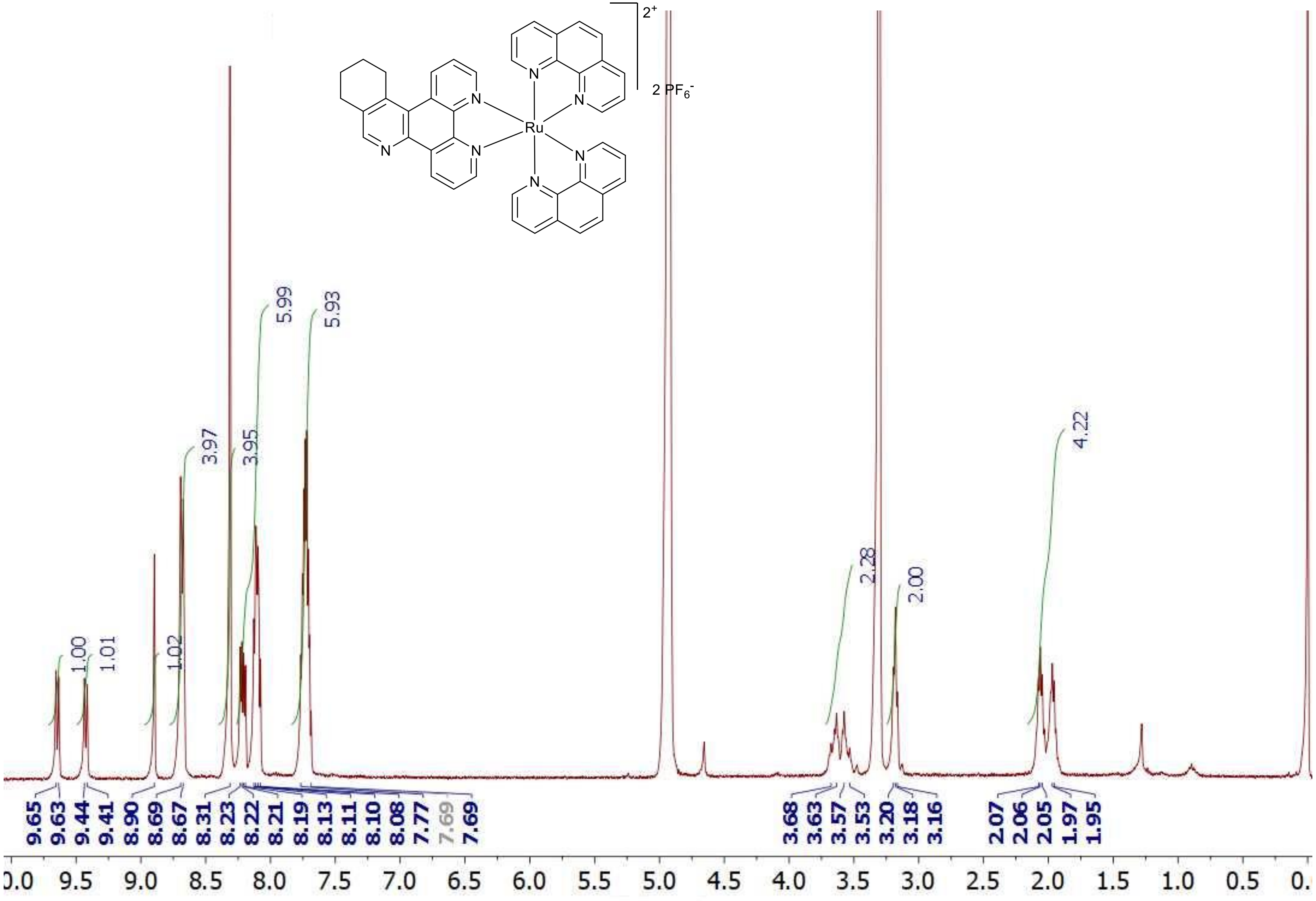

Figure SX. ^{1}H NMR spectrum of VNK-572 in methanol-D4

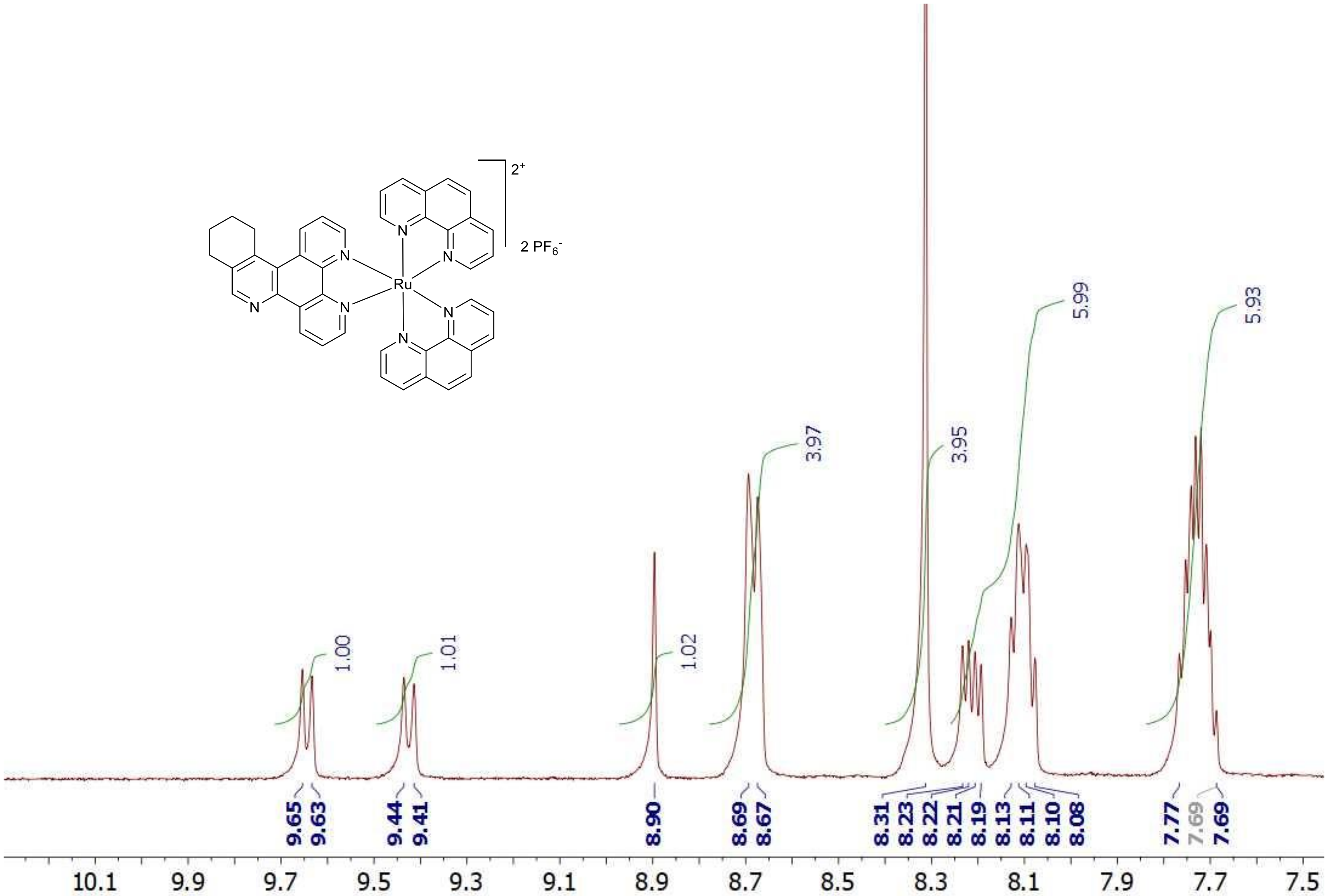

Figure SX. Aromatic region of the ^{1}H NMR spectrum of VNK-572 in methanol-D4

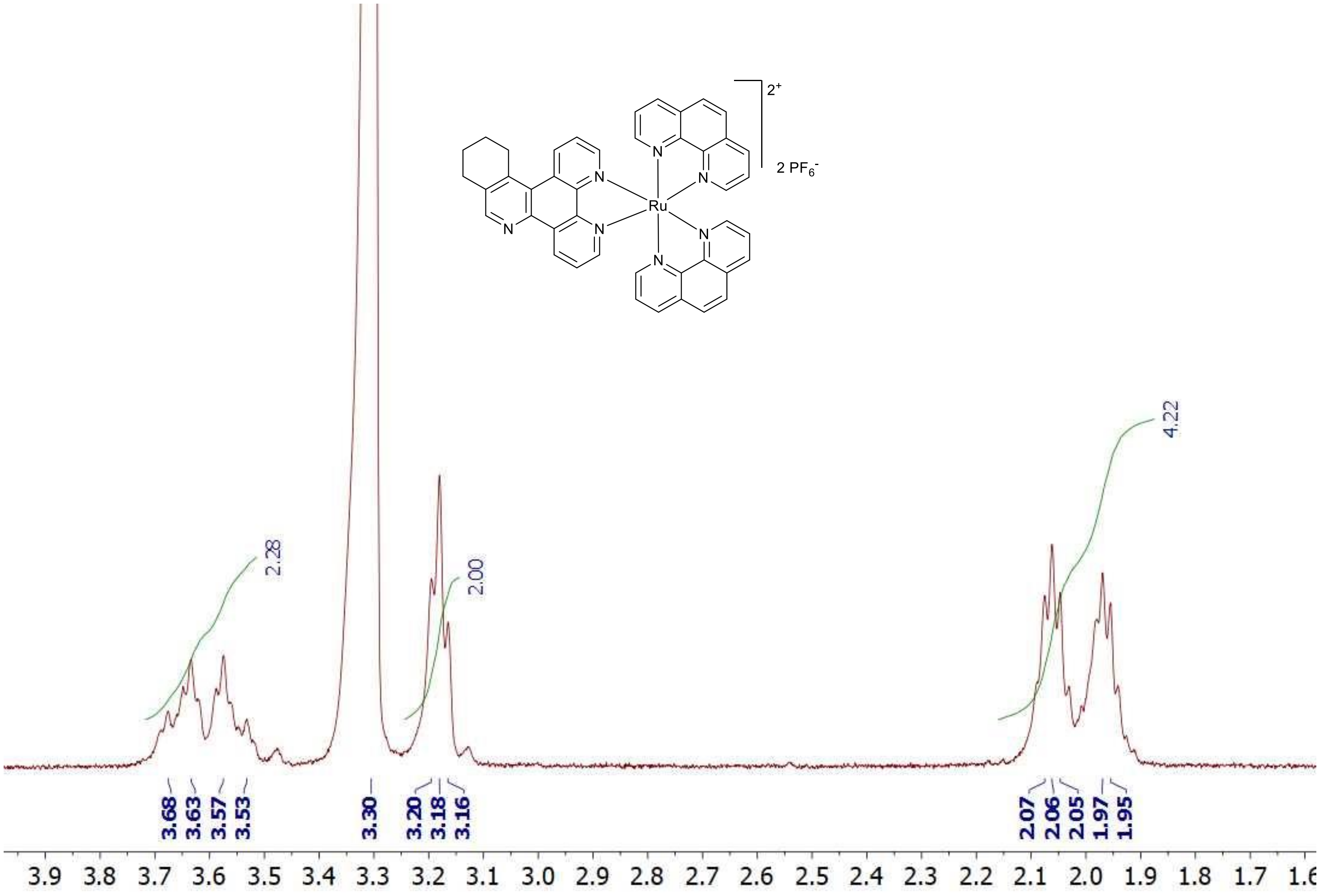

Figure SX. Aliphatic region of the ^{1}H NMR spectrum of VNK-572 in methanol-D4

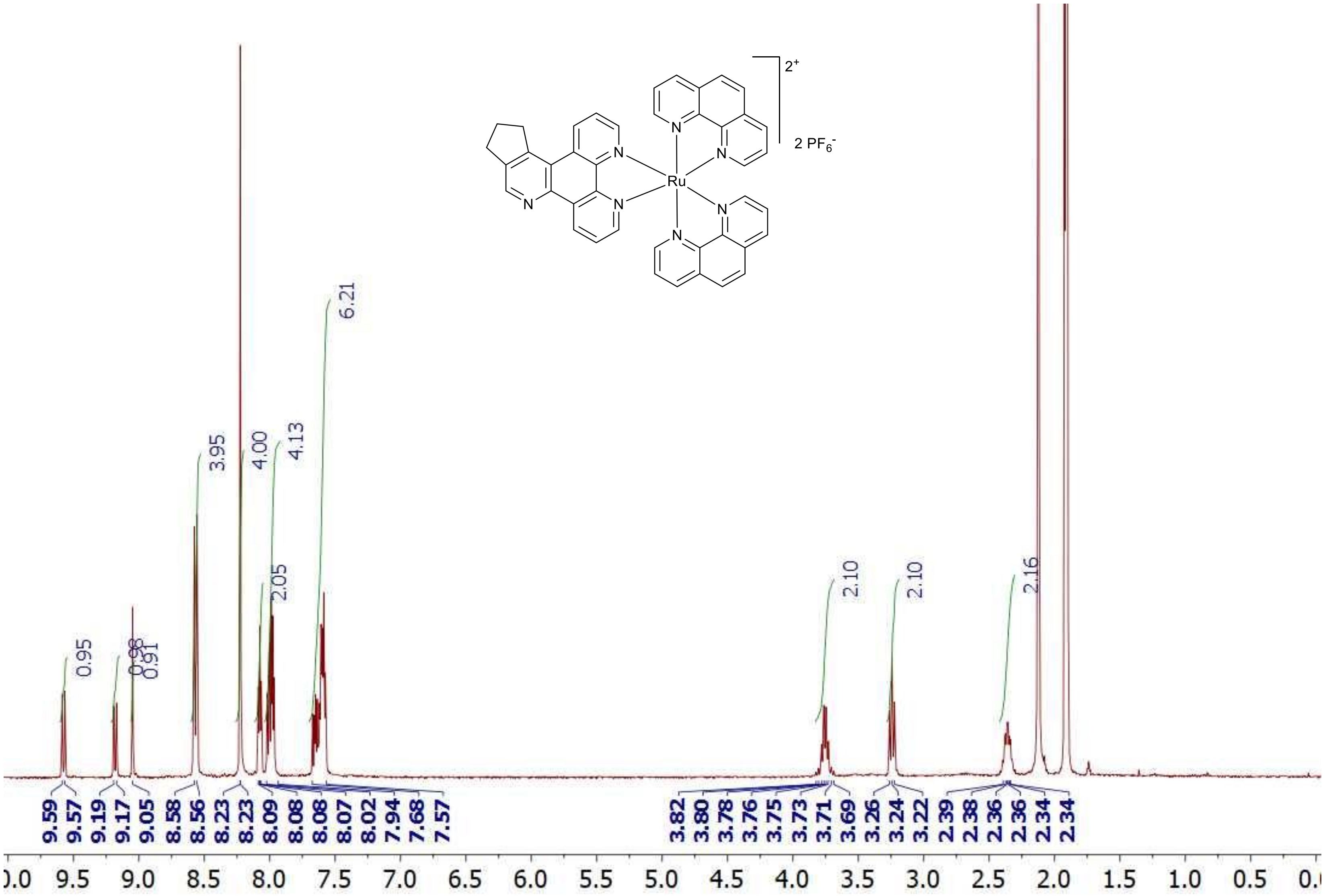

Figure SX. ^{1}H NMR spectrum of VNK-577 in methanol-D4

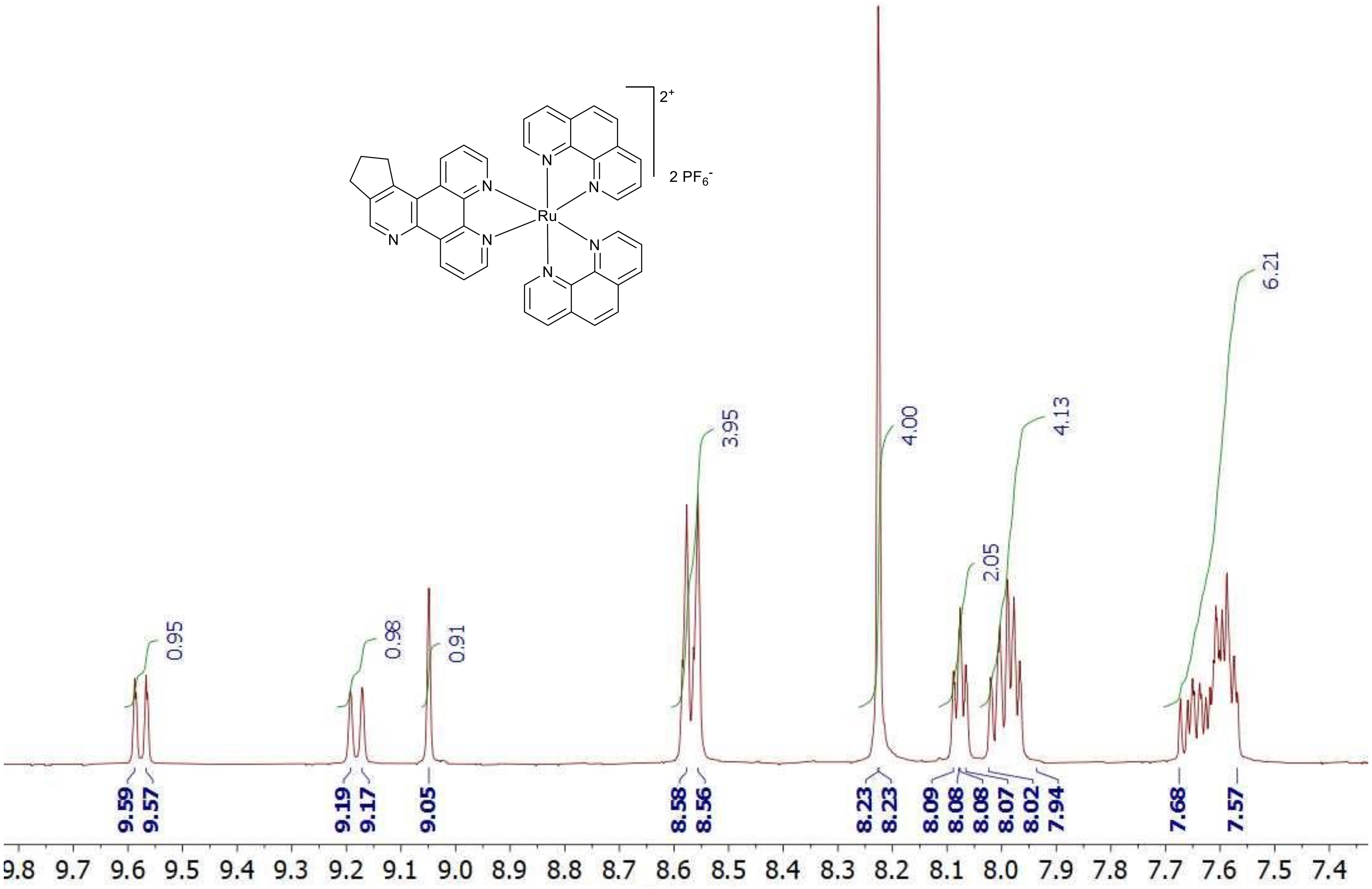

Figure SX. Aromatic region of the ^{1}H NMR spectrum of VNK-577 (POW-12A) in methanol-D4

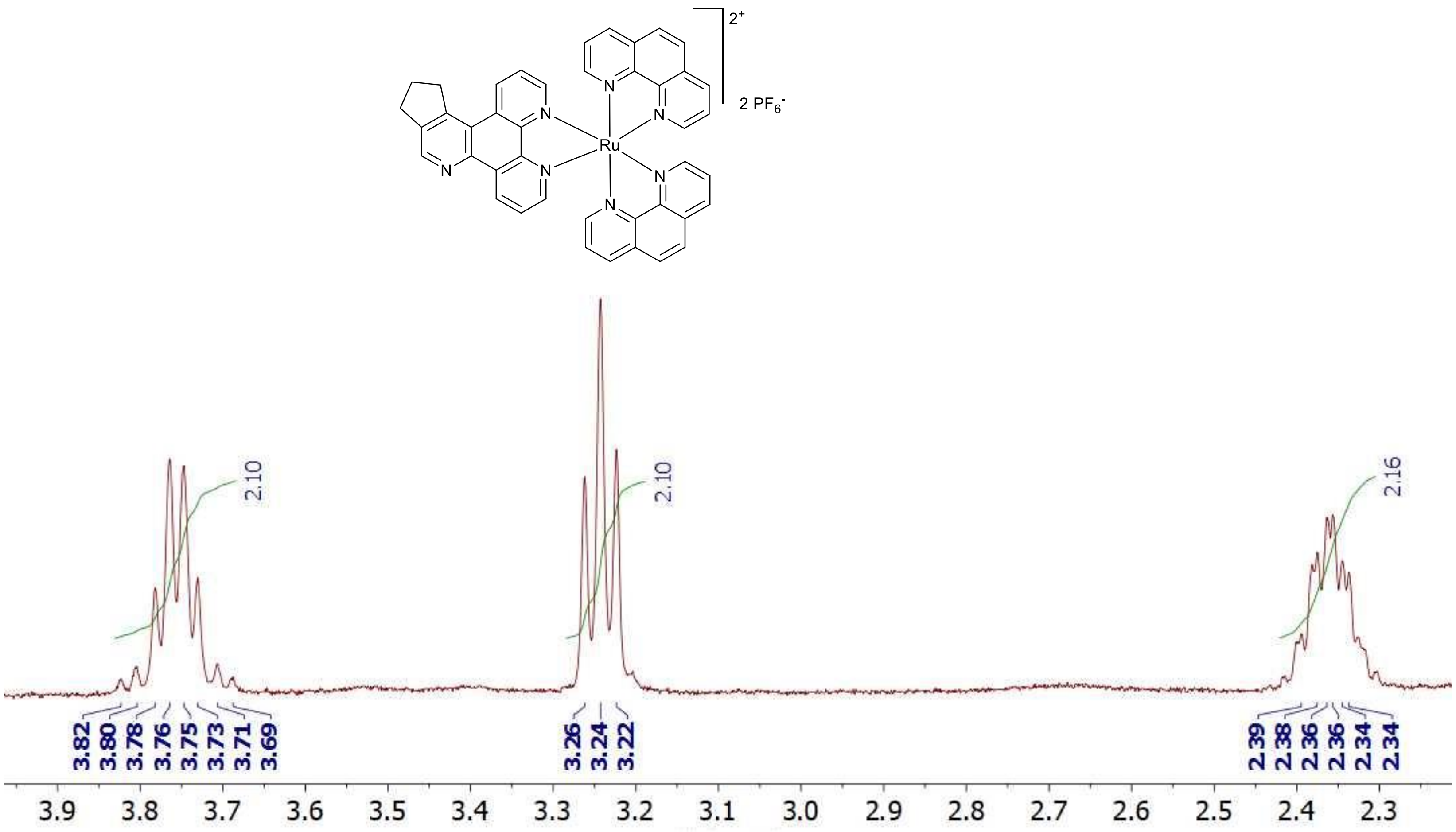

Figure SX. Aliphatic region of the ^{1}H NMR spectrum of VNK-577 (POW-12A) in methanol-D4
